# Biofilm-derived oxylipin 10-HOME–mediated immune response in women with breast implants

**DOI:** 10.1172/JCI165644

**Published:** 2024-02-01

**Authors:** Imran Khan, Robert E. Minto, Christine Kelley-Patteson, Kanhaiya Singh, Lava Timsina, Lily J. Suh, Ethan Rinne, Bruce W. Van Natta, Colby R. Neumann, Ganesh Mohan, Mary Lester, R. Jason VonDerHaar, Rana German, Natascia Marino, Aladdin H. Hassanein, Gayle M. Gordillo, Mark H. Kaplan, Chandan K. Sen, Marshall E. Kadin, Mithun Sinha

**Affiliations:** 1 Division of Plastic Surgery, Department of Surgery, Indiana University School of Medicine, Indianapolis, Indiana, USA.; 2 Department of Chemistry and Chemical Biology, Indiana University–Purdue University Indianapolis, Indianapolis, Indiana, USA.; 3 Meridian Plastic Surgeons, Indianapolis, Indiana, USA.; 4 McGowan Institute for Regenerative Medicine, Department of Surgery, University of Pittsburgh School of Medicine, Pittsburgh, Pennsylvania, USA.; 5 Susan G. Komen Tissue Bank at the IU Simon Comprehensive Cancer Center, Department of Medicine, and; 6 Division of Hematology & Oncology, Department of Medicine, Indiana University School of Medicine, Indianapolis, Indiana, USA.; 7 McGowan Institute for Regenerative Medicine, Department of Plastic Surgery, University of Pittsburgh School of Medicine, Pittsburgh, Pennsylvania, USA.; 8 Department of Microbiology and Immunology, Indiana University School of Medicine, Indianapolis, Indiana, USA.; 9 Department of Dermatology, Roger Williams Medical Center, Boston University School of Medicine, Providence, Rhode Island, USA.; 10 Department of Pathology, University of Virginia, Charlottesville, Virginia, USA.

**Keywords:** Immunology, Inflammation, Adipose tissue, Bacterial infections, Th1 response

## Abstract

This study investigates a mechanistic link of bacterial biofilm–mediated host-pathogen interaction leading to immunological complications associated with breast implant illness (BII). Over 10 million women worldwide have breast implants. In recent years, women have described a constellation of immunological symptoms believed to be related to their breast implants. We report that periprosthetic breast tissue of participants with symptoms associated with BII had increased abundance of biofilm and biofilm-derived oxylipin 10-HOME compared with participants with implants who are without symptoms (non-BII) and participants without implants. *S. epidermidis* biofilm was observed to be higher in the BII group compared with the non-BII group and the normal tissue group. Oxylipin 10-HOME was found to be immunogenically capable of polarizing naive CD4^+^ T cells with a resulting Th1 subtype in vitro and in vivo. Consistently, an abundance of CD4^+^Th1 subtype was observed in the periprosthetic breast tissue and blood of people in the BII group. Mice injected with 10-HOME also had increased Th1 subtype in their blood, akin to patients with BII, and demonstrated fatigue-like symptoms. The identification of an oxylipin-mediated mechanism of immune activation induced by local bacterial biofilm provides insight into the possible pathogenesis of the implant-associated immune symptoms of BII.

## Abstract 2

<p>Immunogenic lipid metabolites induced by a host-biofilm interaction is associated with breast implant illness.</p>

## Graphical abstract

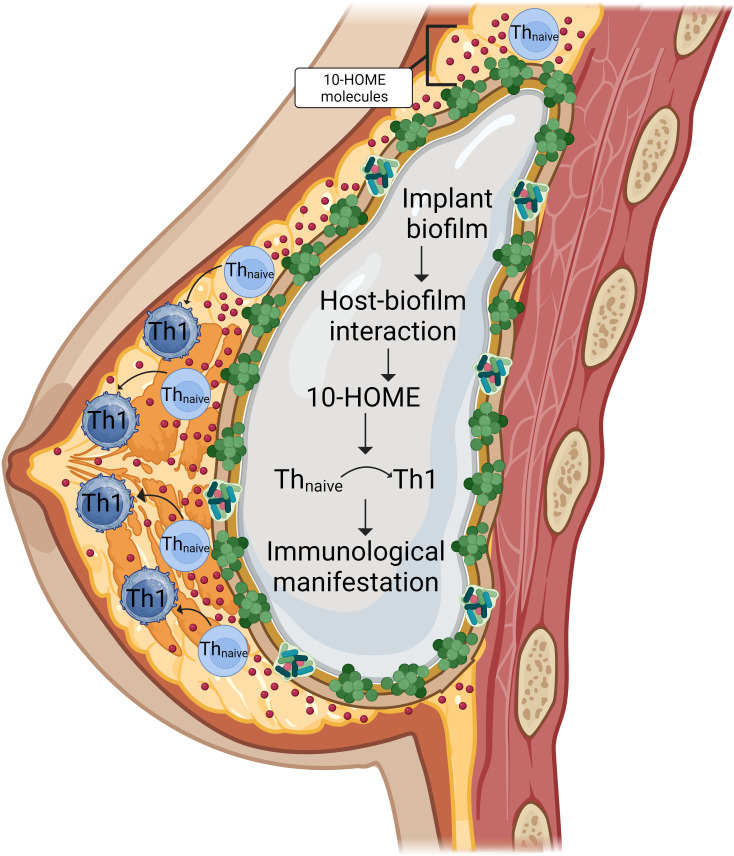

## Introduction

Breast implants were first introduced in 1962. Nearly 60 years later, their safety has continued to be controversial in the medical field, including a period of FDA-mandated restrictions on the use of silicone gel breast implants in the US in the 1990s (1, 2). There are 3.3 million breast cancer survivors in the US (3), many of whom have had implant-based breast reconstruction. Nearly 300,000 women have breast implant surgeries every year in the US (4). A subset of women with breast implants have reported a myriad of nonspecific systemic symptoms (5, 6). The symptoms described include fever, myalgias, chronic fatigue, arthralgias, and other manifestations often associated with autoimmune illnesses (7–15). This constellation of symptoms related to implants has been named breast implant illness (BII) or systemic symptoms associated with breast implants (SSBI).

The number of patients who opt for breast implant explantation due to complications including BII is over 30,000 annually (4). Thus, BII is a growing concern to both patients and surgeons alike, with more than 10 million women worldwide currently having breast implants (7). Despite the increased concern regarding BII, existing scientific literature does not show a definite link between breast implants and autoimmune or connective tissue diseases. Several studies have reported an association of autoimmune symptoms with breast implants (8, 10–14). Symptoms have been documented to begin after placement of the implant and are often relieved by explantation (14, 16, 17). This has led patients and physicians to suspect that breast implants are the likely cause of the observed symptoms (14, 16, 17). However, studies have found silicone gel breast implants to be safe (18). Relevant to this apparent contradiction is the fact that these symptoms have been reported in people with other types of implants, such as orthopedic arthroplasty, which is typically comprised of titanium (14, 19–23). This suggests that the underlying cause of these conditions may be associated with factors other than the implant material. Due to the rising concerns with breast implants for breast implant–associated anaplastic large cell lymphoma (BIA-ALCL) and BII, the US FDA placed a black box warning on breast implants (24). Therefore, it is important to decipher the underlying molecular mechanism(s) associated with BII for a better understanding of all implant-related illnesses with systemic immunological manifestations.

Immune cells have been studied to evaluate the biocompatibility of breast implant surfaces (25–27). Earlier studies showed the presence of activated CD4^+^ T cells in the periprosthetic tissue of capsule/silicone implant from the recovered biopsies (28–30). T lymphocytes that were found at the implant site had been suggested to be due to the prolonged action of *Staphylococcus* antigens, which resulted in stimulation of lymphocytes leading to clonal expansion of activated T cells (31–33). Most reports on infection and the T cell axis were focused on antigen-specific T cell responses (34, 35). Bystander activation of CD4^+^ T cells and the role of antigen unrelated CD4^+^ T cells in various infections might lead to the development of autoimmune diseases, but the mechanisms involved have not been identified (36, 37). Bacterial products, such as oxidized lipids or lipopolysaccharides (LPS), bind to the surface of naive T cells and result in IFN-α/β secretion, which further contributes to the proliferation and expansion of heterologous polyclonal T cells (38–40).

To our knowledge, this is the first study to investigate in depth the possible role of bacterial biofilm as one of the factors in the pathogenesis of BII via a mechanistic pathway and through a patient-based study. The biofilm-derived molecule oxylipin (E)-10-hydroxy-8-octadecenoic acid (10-HOME) could be a potential therapeutic target to intervene in implant-associated immunological complications.

## Results

### Bacterial biofilm in implants is associated with BII.

**Figure 1:**
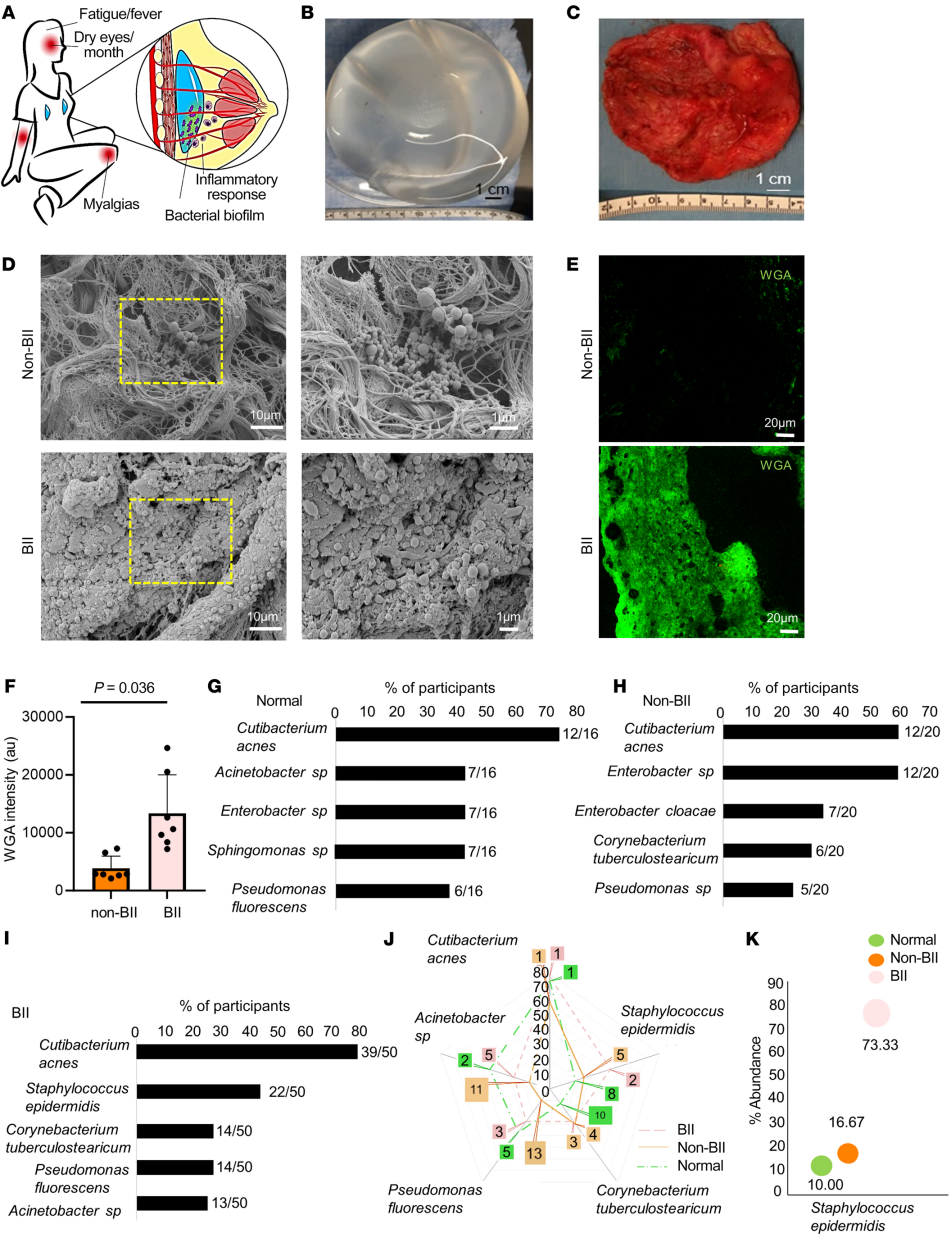
Bacterial biofilm in implants is associated with BII. (**A**) Schematic presentation of the bacterial biofilm association with BII. (**B**) Breast implant isolated from a participant. (**C**) Capsule associated with breast implant of the participant shown in panel **B**. (**D**) Increased abundance of bacterial biofilm from the implant-associated capsule of BII compared with non-BII implant-associated capsules as determined through scanning electron microscopy. Zoomed insets of region of interest (ROI) are shown by the dotted yellow square. *n* = 10 (non-BII), *n* = 25 (BII) participants. (**E**) Increased abundance of bacterial biofilm as measured through WGA assay in the capsules of BII participants compared with the non-BII capsules. (**F**) Quantification of biofilm aggregates using WGA staining. Data presented as mean ± SEM, *n* = 7 (non-BII), *n* = 7 (BII) participants. *t* test was used for analysis of BII versus non-BII (*P* = 0.0036). (**G**–**I**) 16S rRNA NGS-based bacterial typing from the breast tissues of participants with **G**, normal; **H**, non-BII; and **I**, BII tissues. Top 5 bacterial species in each group represented. Fraction of participant samples associated with a bacterial species is provided in parenthesis *n* = 16 (normal), *n* = 20 (non-BII), *n* = 50 (BII). (**J**) Spider-plot depicting the ranking of the top 5 bacterial infection types in the BII group compared to their ranking in the non-BII and normal groups. The intersection of the group lines with the spikes of the spider-plot indicates the proportion of these infections by groups. The call out numbers are the ranks for each of these infections within each group. *n* = 16 (normal), *n* = 20 (non-BII), *n* = 50 (BII). (**K**) Increased abundance of biofilm forming *Staphylococcus epidermidis* in implant-associated breast tissues of participants with BII. Bubble plot indicating the percentage of patients with *Staphylococcus epidermidis* provided above the individual bars. The bubble percentage indicates the likelihood of a participant with *S*. *epidermidis* biofilm in each of the 3 groups. *n* = 16 (normal), *n* = 20 (non-BII), *n* = 50 (BII) participants.

The study included 178 individuals divided in 3 cohorts. A total of 86 patients with BII manifestations were analyzed. Participants were diagnosed with BII using clinical parameters outlined in previous studies (7–13) (Figure 1A). As a part of the diagnosis, the patients were required to complete a questionnaire (Supplemental Table 1; supplemental material available online with this article; https://doi.org/10.1172/JCI165644DS1). The questionnaire screened for the commonly reported symptoms associated with BII (7–13). Implants, associated capsules, periprosthetic breast tissue, and peripheral blood were collected from the participants (Figure 1, B and C), as described in the Methods section. The mean age of the patients with BII was 48.2 years with mean duration of implant insertion of 12.89 years. Two groups were considered as controls. Control group I (non-BII, *n* = 55) included patients with breast implants without BII symptoms but who underwent explantation of the breast implant. The mean age of non-BII patients was 53.3 years, with the mean duration of implant insertion of 13.2 years. Control group II (normal tissue, *n* = 37) comprised women without an implant, whose breast tissue was removed as a clinically indicated surgical procedure such as mastopexy or breast reduction. The mean age of the people in control group II was 42.17 years. The demographics of the participants have been provided in Supplemental Table 2.

Bacterial biofilm was detected in implant-associated capsules through scanning electron microscopy (Figure 1D and Supplemental Figure 1A). Though biofilm was detected in the capsules of implants from participants who qualified as BII and non-BII, the abundance of biofilm was significantly higher in the capsules of individuals in the BII group, as observed through wheat germ agglutin (WGA) assay(*P* = 0.0036) (Figure 1, E and F). The microbiological culture analyses resulted in limited or no growth of bacterial colonies (Supplemental Table 3). It has been reported that bacterial biofilms are difficult to detect through colony-forming assays due to their subdued metabolism (41, 42). Hence, in cases of bacterial biofilm, next generation sequencing (NGS) using the variable region of bacterial 16S rRNA gene is employed to type bacteria and determine their abundance (43). Diverse types of biofilm-forming bacteria were observed associated with normal tissues, non-BII, and BII tissues (Figure 1, G–I) through NGS of the 16S rRNA variable region. Most of the species identified were opportunistic bacteria associated with normal skin flora capable of forming biofilms (Figure 1, G–I). Comparative ranked analyses with the normal, non-BII, and BII groups revealed an increased abundance of *Staphylococcus epidermidis* in BII (Figure 1, J and K). The other bacteria found in increased abundance in the breast tissues of the 3 cohorts was *Cutibacterium acnes* (previously known as *Propionibacterium acnes*). Bivariate analysis using cross-tabulation was performed between presence of biofilm and the study groups. Using the 2-sample test of proportions with *z* tests, *S*. *epidermidis* colonization was observed to be higher in the BII group (73.33%) compared with the non-BII group (16.67%, *P* = 0.018) and the normal group (10%, *P* = 0.036). The BII group was 2.4 times more likely to have *S*. *epidermidis* colonization compared with the non-BII group (odds ratio = 2.4). Similarly, when comparing with the normal group, the BII group was 3.4 times more likely to have *S*. *epidermidis*. Both the spider and the bubble plot indicate that *S*. *epidermidis* was found in increased abundance in the BII group compared with the normal or non-BII groups (Figure 1, J and K). Within the BII group, we ranked the bacterial types using proportion and obtained the top 5 colonizations (*C*. *acnes, S*. *epidermidis, Corynebacterium tuberculostearicum, Pseudomonas fluorescens, and Acinetobacter sp.*) (Figure 1I). We used these bacteria to examine their ranking across the groups (Supplemental Figure 1B). Compared with the BII group, the proportion of each of these 5 bacteria colonizations were about 46% to 86% lower in the other 2 groups. Of note, *C*. *acnes* was consistently the highest ranked bacterial type across groups, though the same was not true for *S*. *epidermidis,* which was more predominant in the BII cohort (Figure 1J). Increased abundance of *C*. *tuberculostearicum* was also found in periprosthetic tissue of BII samples compared with normal tissue (Supplemental Figure 1B). However, the study did not observe a significant difference in abundance of *C*. *tuberculostearicum* between the BII and the non-BII cohort.

### Increased abundance of biofilm derived 10-HOME in participants with BII.

**Figure 2:**
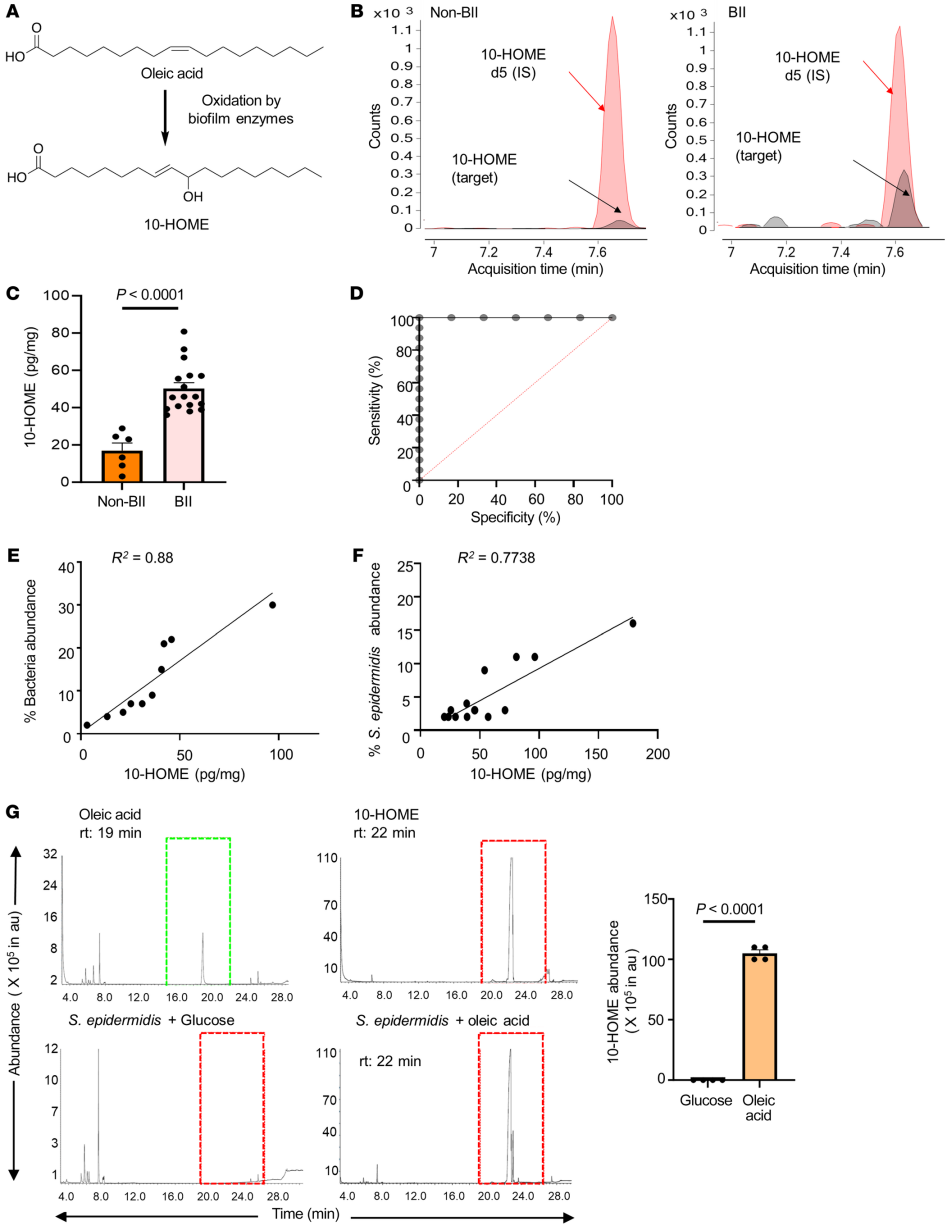
Increased abundance of biofilm-derived 10-HOME in participants with BII. (**A**) Schematic of formation of 10-HOME from oleic acid. (**B**–**D**). Increased abundance of 10-HOME in implant-associated tissue of participants with BII. (**B**) Chromatograms of 10-HOME from non-BII and BII tissues using LC-MS/MS targeted analyses. (**C**) Data presented as mean ± SEM, *n* = 6 (non-BII), *n* = 17 (BII), *t* test was used to determine BII versus non-BII (*P* < 0.0001). (**D**) Receiver operating characteristic (ROC) curve analysis to determine specificity and sensitivity of 10-HOME detection. (**E**) Increased abundance of bacteria associated with 10-HOME detected from the implant-associated tissue of participants with BII. (**F**) Increased abundance of *Staphylococcus epidermidis* associated with 10-HOME detected from the implant-associated tissue of participants with BII. (**G**) Synthesis of 10-HOME by *S*. *epidermidis* in vitro upon using oleic acid as carbon source. Gas chromatography-mass spectrometry analyses for detection of 10-HOME derivatized both as trimethylsilyl ethers and methyl estersoleic acid standard, 10-HOME standard, *S*. *epidermidis* with glucose as carbon source, *S*. *epidermidis* with oleic acid as carbon source, quantification of 10-HOME abundance. *n* = 4. *t* test was used to determine glucose versus oleic acid (*P* < 0.0001).

Previous reports identified oxylipins as molecules mediating the interaction between host and bacteria (44–46). The oxylipin 10-HOME is formed by the bacterial oxidation of oleic acid (47) (Figure 2A). Oxylipin 10-HOME has been reported to inhibit flagellum-driven swimming and swarming motilities and stimulate the formation of bacterial biofilms in vitro (48). The oxylipin 10-HOME was synthesized in the laboratory in natural isotopic abundance (light) isotope and deuterated (heavy) isotope forms. The synthesized 10-HOME was validated through high-resolution mass spectrometry, thin-layer chromatography, LCMS and NMR spectroscopy (Supplemental Figures 2–9). We explored the role of 10-HOME in the association between BII-related symptoms and bacterial biofilm.

By liquid chromatography-mass spectrometry (LC-MS/MS), we observed elevated levels of 10-HOME in implant-associated periprosthetic tissue of BII compared with non-BII samples, *P* < 0.0001 (Figure 2, B–D, and Supplemental Figures 10 and 11). In the age-adjusted bootstrapped analysis, BII periprosthetic tissue had 28.13 units higher levels of 10-HOME relative to samples from individuals in the non-BII group. A positive correlation was observed between bacterial abundance and 10-HOME concentration in periprosthetic tissue of individuals with BII (Figure 2E). For every unit increase in 10-HOME levels, the percentage bacterial abundance increased by 0.34 units (*P* = 0.022). Similar correlation was observed in participants with BII with *Staphylococcus epidermidis* (Figure 2F). To determine if *S*. *epidermidis* was capable of synthesizing 10-HOME, it was cultured in vitro in basic M9 salt media with oleic acid as a source of carbon. Formation of 10-HOME was detected using gas chromatography-mass spectrometry and LC-MS/MS (Figure 2G and Supplemental Figure 1, C–E). In addition to *S*. *epidermidis*, we tested 7 bacterial species (from 3 cohorts, Figure 1, G–I), 4 species didn’t produce 10-HOME or produced it at very low or undetectable levels through LC-MS/MS. Three species (including *S*. *epidermidis*) produced 10-HOME (Supplemental Figure 1F). The other 2 species, *Pseudomonas fluorescens* and *Acinetobacter sp*., were also present in non BII and normal breast microflora and had a lower abundance (Figure 1, G–I). Oxylipins have been reported to cause immune activation via alteration in expression of transcripts in immunological pathways (49). We subsequently explored the global transcriptome in the BII cohort.

### Comparative transcriptomic and molecular pathway analyses of participants with BII.

**Figure 3:**
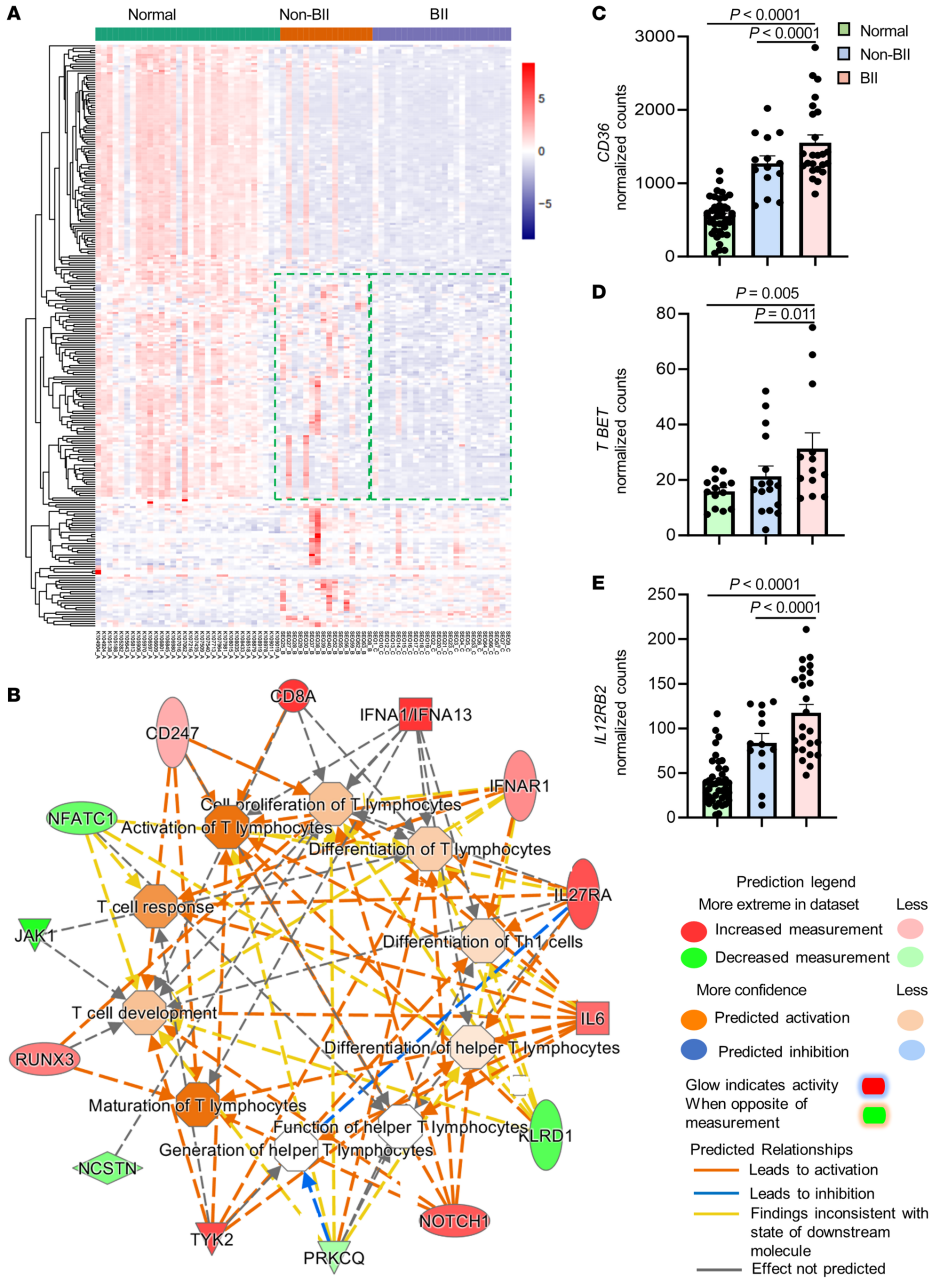
Comparative transcriptomic and molecular pathway analyses of participants with BII. (**A**) Hierarchical clustering of genes with at least 2-fold change and controlled by FDR of 0.05 breast tissue from participants in the normal (*n* = 34), non-BII (*n* = 16), and BII (*n* = 24) groups. (**B**) Gene interaction networks for functions related to differentiation of T-lymphocytes, differentiation of Th1 cells, cell proliferation of T-lymphocytes, maturation of T-lymphocytes, generation of T-helper lymphocytes, differentiation of helper T-lymphocytes, T cell response, T cell development, and quantity of T-lymphocytes in BII specimens. Represented functional networks relevant to the set of imported genes generated by the canonical pathway function relevant to Th1 pathway. The list was selected from the hierarchical cluster of Th1 genes; upregulated genes are shown in red and downregulated in green. The intensity of red and green corresponds to an increase and decrease, respectively, in log_2_ fold change. (**C**) Comparison of normalized RNA-Seq counts for *CD36* between samples from normal, non-BII, and BII groups. In an age-adjusted nonparametric regression model, compared with participants in the normal group, normalized number of *CD36* counts was approximately 1,116 units higher in the BII group (*P* < 0.0001) and 650 units higher in the non-BII group (*P* < 0.0001). (**D**) Comparison of normalized RNA-Seq counts for Th1 gene *T-BET* *(TBX21)* between normal, non-BII, and BII samples. In an age-adjusted nonparametric regression model, compared with participants in the normal group, the normalized number of *T-BET* counts was approximately 18 units higher in the BII group (*P* = 0.005) and 11 units higher in the non-BII group (*P* = 0.011). (**E**) Comparison of normalized RNA-Seq counts for Th1 gene *IL12RB2* between normal, non-BII, and BII samples. In an age-adjusted nonparametric regression model, compared with participants in the normal group, the normalized number of *IL12RB2* counts was approximately 84 units higher in the BII group (*P* < 0.0001) and 45 units higher in the non-BII group (*P* < 0.0001).

To explore the mechanisms underlying BII, we performed bulk RNA-Seq with a depth of 30 million reads on periprosthetic tissue from participants in the BII and non-BII groups (Data available through Gene Expression Omnibus no. GSE178425) and compared it to the RNA-Seq database (GSE164641) of normal breast tissue (an anatomically similar location to periprosthetic breast tissues). Findings from normal breast tissue (*n* = 34), non-BII (*n* = 16), and BII (*n* = 24) tissues revealed differential expression of 16,428 transcripts between normal breast tissue versus periprosthetic tissue (non-BII and BII) (Figure 3A). This implied that placement of the implant had a profound effect on the alteration of the local (periprosthetic breast tissue) transcriptome (Figure 3A). Upon comparison between the BII versus non-BII cohorts, 2,878 genes were differentially expressed (Figure 3A and Supplemental Figure 12). We observed altered gene expression related to adaptive T cell response in participants in the BII compared with the non-BII and normal tissue groups (Figure 3, B–E, and Supplemental Figure 12). Molecular network analysis also suggested the involvement of T cell pathways (Figure 3B). Canonical pathways identified through ingenuity pathway analysis (IPA) revealed that the majority of the differentially expressed genes associated with transcripts belong to Th1 pathway (Supplemental Figure 12). Increased *CD36* (fatty acyl translocase) (Figure 3C) and Th1-responsive genes including the Th1 specific transcription factor, *TBET (TBX21),* were observed in the BII cohort (Figure 3, D and E). Th1 cells are associated with an autoimmune response in multiple illnesses, including rheumatoid arthritis. CD36 is a fatty acyl translocase; its level is upregulated when uptake of fatty acids (normal or oxidized) is required.

### Abundance of CD4^+^ Th1 cells in implant-associated tissue of participants with BII.

**Figure 4:**
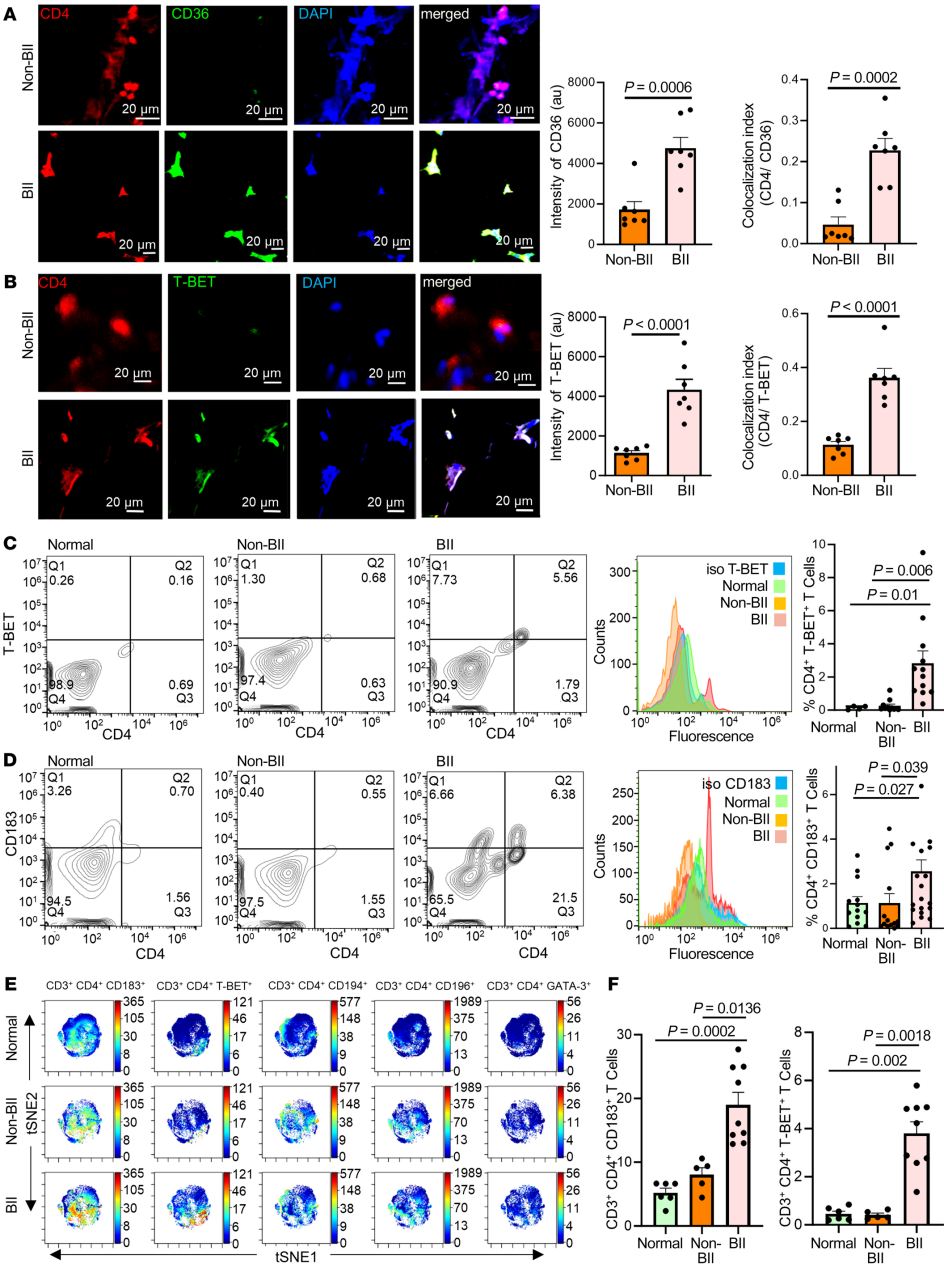
Abundance of CD4^+^ Th1 cells in implant-associated tissue of participants with BII. (**A**) Increased expression of CD36 in breast tissue associated with participants with BII, immunostained with anti-CD4 (red), anti-CD36 (green), and DAPI (blue). Data presented as mean ± SEM, (*n* = 7). Scale bar: 20 μm. A *t* test was used to determine BII versus non-BII intensity (*P* = 0.0006), colocalization (*P* = 0.0002). (**B**) Increased expression of T-BET in breast tissue associated with participants with BII, immunostained with anti-CD4 (red), anti-T-BET (green), and DAPI (blue). Data presented as mean ± SEM, (*n* = 7). Scale bar: 20 μm. A *t* test was used to determine BII versus non-BII Intensity (*P* < 0.0001) and colocalization (*P* < 0.0001). (**C**) Flow cytometry with anti-CD4 (FITC) and anti-TBET (PE). Representative plots: normal, BII, histogram with isotype control for T-BET. Data presented as mean ± SEM, *n* = 4 (normal), *n* = 11 (non-BII), and *n* = 12 (BII). Bivariate Kruskal-Wallis with posthoc Benjamini-Hochberg adjusted pairwise comparison analysis was used to compare BII versus normal (*P* = 0.01) and BII versus non-BII (*P* = 0.006). (**D**) Elevated Th1 subtype in the peripheral blood of participants with BII. Flow cytometry analyses peripheral blood of participants stained with anti-CD4 (FITC) and anti-CD183 (PE). Representative flow plots: normal, non-BII, BII, histogram with isotype control for CD183. Data presented as mean ± SEM, *n* = 13 (normal), *n* = 14 (non-BII), *n* = 20 (BII). Bivariate Kruskal-Wallis with posthoc Benjamini-Hochberg adjusted pairwise comparison analysis was used to determine BII versus normal (*P* = 0.027) and BII versus non-BII (*P* = 0.039). (**E**) Representative viSNE plots for CD3^+^ CD4^+^ T cells. Color depicts the intensity of the marker labeled on arcsinh scales from blue (low) to red (high). The analyses indicated elevated Th1 subtype in the peripheral blood of participants with BII. (**F**) Quantification of median marker expression using CytoBank software for panel **E**, CD183 and T-BET, *n* = 6 (normal), *n* = 5 (non-BII), and *n* = 9 (BII). Bivariate Kruskal-Wallis with posthoc Benjamini-Hochberg adjusted pairwise comparison analysis were used for analyses. For CD183, BII versus normal (*P* = 0.0002); BII versus non-BII (*P* = 0.0136); for T-BET, BII versus normal (*P* = 0.002); BII versus non-BII (*P* = 0.0018). The quantification of the remainder of viSNE plots is in Supplemental Figure 15.

In agreement with the RNA-Seq data, periprosthetic breast tissue associated with the implant of participants with BII showed an increase of CD4^+^CD36^+^ cells (Figure 4A). The CD4^+^ T cells associated with participants with BII were T-BET^+^ compared with those of the non-BII and normal breast tissue groups, as observed through IHC (Figure 4B). The T-BET transcription factor is critical in Th1 subtype determination. Because BII is reported as a systemic immune manifestation, peripheral blood of participants in the BII and non-BII groups was analyzed for CD4^+^ T cells (Th1, Th2, Th9, and Th22) using flow cytometry and (Th1, Th2, Th9, Th17, Th22, and Treg) using mass cytometry time of flight (CyTOF). An increase of Th1 cells was observed in participants with BII as measured through flow cytometry using T-BET (Figure 4C and Supplemental Figure 13) and CD183/CXCR3 (Th1 cell markers) (Figure 4D and Supplemental Figure 13), compared with participants in the non-BII and normal groups. No significant difference was observed in other Th subtypes, including Th2 (CD194 and GATA3), Th9, or Th22 (CD196) between people in the BII and non-BII groups (Supplemental Figure 13 and Supplemental Figure 15, A–C). The following human cell lines were used as positive controls for validation of surface antigens: Mac2A for CD183 (50), Mac2B for CD194 (50) and TLBR1 for CD196 (51) (Supplemental Figure 14, A–C). The increase of the Th1 cytokine IFN-γ (Supplemental Figure 13, D and F) in breast tissue and serum of people with BII and relatively unchanged levels of Th2 cytokine IL-10 (Supplemental Figure 13, E and G) further supports specific Th1 activation.

Assessment through CyToF showed that the T cells isolated from the peripheral blood of individuals with BII exhibited an increase of Th1 phenotype (Figure 4, E and F, Supplemental Figure 15, A–C, and Supplemental Figure 16). CyTOF analyses did not reveal any significant difference in Th2, Th9, Th22, Th17 and Treg populations between the 3 cohorts (Supplemental Figure 15, D–H and Supplemental Figure 16). However, these results do not definitively establish that 10-HOME led to CD4^+^Th1 cell induction or that 10-HOME can lead to CD36 upregulation. Thus, we studied the effect of 10-HOME on human primary naive CD4^+^ T cells.

### Oxylipin 10-HOME polarizes naive CD4^+^ T cells to Th1 subtype in vitro.

**Figure 5:**
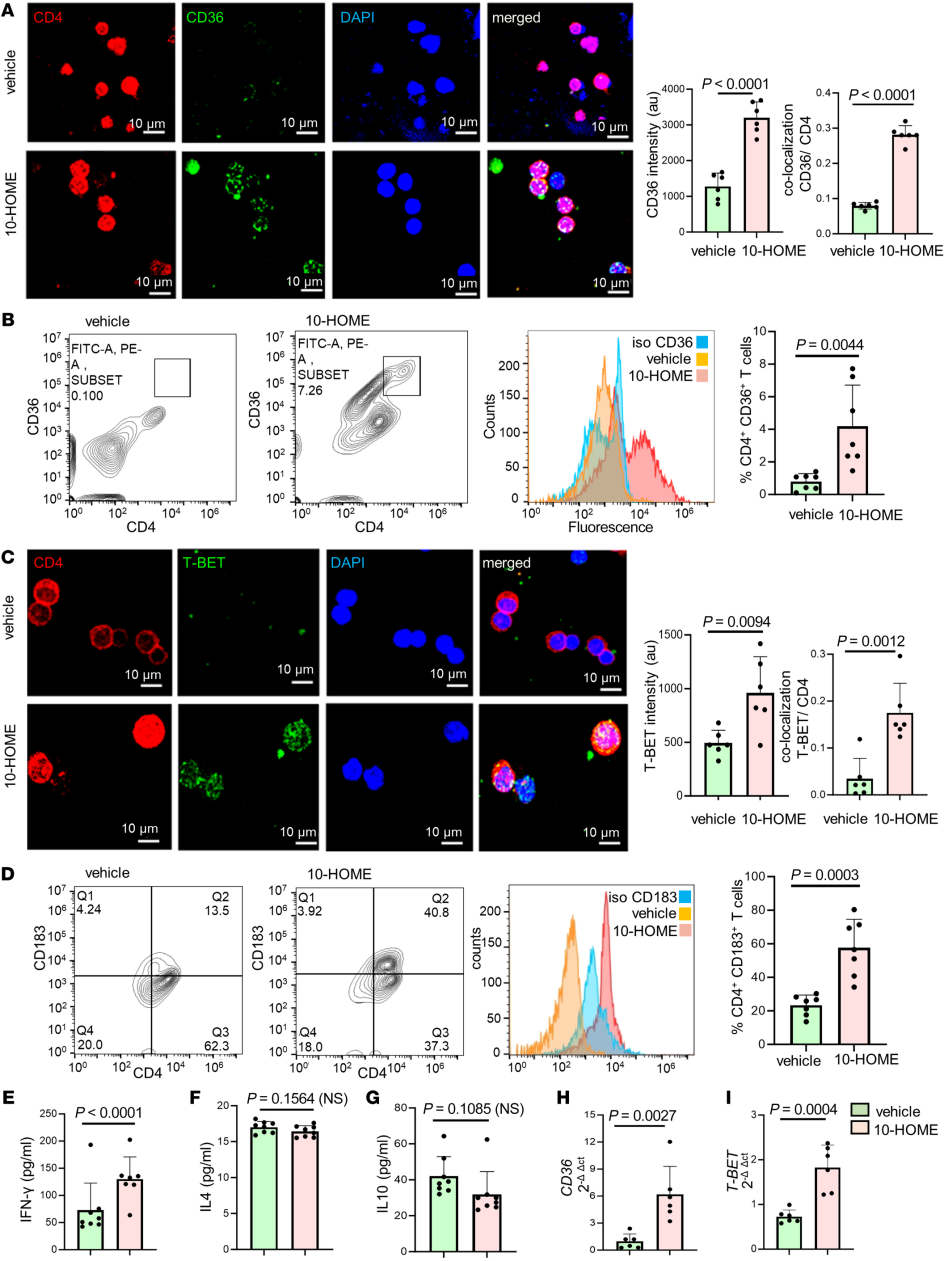
Oxylipin 10-HOME polarizes naive CD4^+^ T cells to Th1 subtype in vitro. (**A**) Increased expression of CD36 in naive CD4^+^ T cells treated with 10-HOME immunostained with anti-CD4 (red), anti-CD36 (green), and DAPI (blue). We quantified the fluorescent intensity of CD36. Colocalization of CD4 and CD36 depicted increased abundance of CD4^+^ CD36^+^ cells. Data presented as mean ± SD, (*n* = 6). Scale bar: 10 μm. A *t* test was used to determine vehicle versus 10-HOME, intensity (*P* < 0.0001), colocalization (*P* < 0.0001). (**B**) We found elevated CD36 in the 10-HOME treated naive T cells. Flow cytometry analyses of treated cells stained with anti-CD4 (FITC) and anti-CD36 (APC). Representative plots: vehicle, 10-HOME-treated cells, histograms with isotype control for CD36. Data presented as mean ± SD, (*n* = 7). *t* test was used to determine vehicle versus 10-HOME(*P* = 0.0044). (**C**) Increased expression of T-BET in the 10-HOME–treated naive CD4^+^ T cells immunostained with anti-CD4 (red), anti-TBET (green), and DAPI (blue). We quantified the fluorescent intensity of TBET. Colocalization of CD4 and TBET depicted increased abundance of CD4^+^ TBET^+^ cells. Data presented as mean ± SD, (*n* = 6). Scale bar: 10 μm. A *t* test was used to determine vehicle versus 10-HOME intensity (*P* = 0.0094) and colocalization(*P* = 0.0012). (**D**) Elevated Th1 subtype (CD183^+^) in the 10-HOME–treated naive CD4^+^ T cells. Flow cytometry analyses with anti-CD4 (FITC) and anti-CD183 (PE). Representative plots: vehicle-treated, 10-HOME–treated cells, and histograms of cells with isotype control for CD183. Data presented as mean ± SD, (*n* = 7). *t* test was used to determine vehicle versus 10-HOME Intensity (*P* = 0.0094) and colocalization (*P* = 0.0003). (**E**) Increased expression of IFN-γ in the 10-HOME–treated naive CD4^+^ T cells as measured through ELISA. Data presented as mean ± SD (*n* = 7). *t* test was used to determine vehicle versus 10-HOME Intensity (*P* = 0.0094), colocalization(*P* < 0.0001). (**F** and **G**) There was no significant change in IL4 and IL10 following 10-HOME treatment of naive CD4^+^ T cells. Data presented as mean ± SD, (*n* = 8). *t* test was used to determine vehicle versus 10-HOME, 1L4 (*P* = 0.1564), IL10 (*P* = 0.1085). (**H** and **I**) Increased expression of (**H**) *CD36* and (**I**) *T-BET* in 10-HOME–treated naive T cells. Data presented as mean ± SD, (*n* = 6). *t* test was used to determine vehicle versus 10-HOME *CD36* (*P* = 0.0027) and *T-BET* (*P* = 0.0004).

In order to study the effect of 10-HOME on T cells, naive CD4^+^ T cells, which were isolated from healthy human peripheral blood mononuclear cells, were treated with 100 μM 10-HOME for 48 hours. Naive CD4^+^ T cells are not antigen challenged and thus are not committed to any specific subtype. Increased CD36 expression was observed in the 10-HOME–treated CD4^+^ T cells through immunocytochemistry (Figure 5A), flow cytometry (Figure 5B), and quantitative real time PCR (Figure 5H) indicative of the 10-HOME–mediated induction of CD36. Polarization to the Th1 subtype occurred in the presence of 10-HOME, as observed through immunocytochemistry (Figure 5C), flow cytometry (Figure 5D), and quantitative real time PCR (Figure 5I). The CD4^+^ cells exhibited increased expression of T-BET (a transcription factor activated during the polarization of naive T cells to the Th1 subtype) (Figure 5, C and I, and Supplemental Figure 17A), CD183/CXCR3 (CD4^+^ Th1 cell marker) (Figure 5D), and Th1-secreted proinflammatory cytokine IFN-γ through ELISA (Figure 5E). The CD183^+^ Th1 cells exhibited increased abundance of CD36 marker (Supplemental Figure 17B). The other subtypes of CD4^+^ T cells (Th2, Th9, and Th22) assayed did not exhibit any statistically significant increase in population in post–10-HOME treatment on naive CD4^+^ T cells. Th2 cells were assayed using surface marker CD194/CCR4 (Supplemental Figure 17C), transcription factor GATA3 (Supplemental Figure 17D), and antiinflammatory cytokines IL-4 and IL-10 (Figure 5, F and G). Th9 and Th22 cells were assayed using surface marker CD196/CCR6 (Supplemental Figure 17E). CD4 T cells were exposed to the media obtained from *S*. *epidermidis*–treated cells. Upon analyzing the supernatant collected from the coculture, we observed polarization to the Th1 subset (increased abundance of CD183^+^ cells). However, using the bacterial supernatant increased the population size of CD194^+^ cells, which corresponded to the Th2 subtype. No change in CD196 (Th9 and Th22 markers) levels were observed. Bacterial supernatant is heterogenous, unlike 10-HOME only. The other factors present in the supernatant may be triggering the polarization of CD194 expressing along with CD183 expressing T cells (Supplemental Figure 18).

### Elevated CD4^+^ Th1 and fatigue-like symptoms in mice administered with 10-HOME.

**Figure 6:**
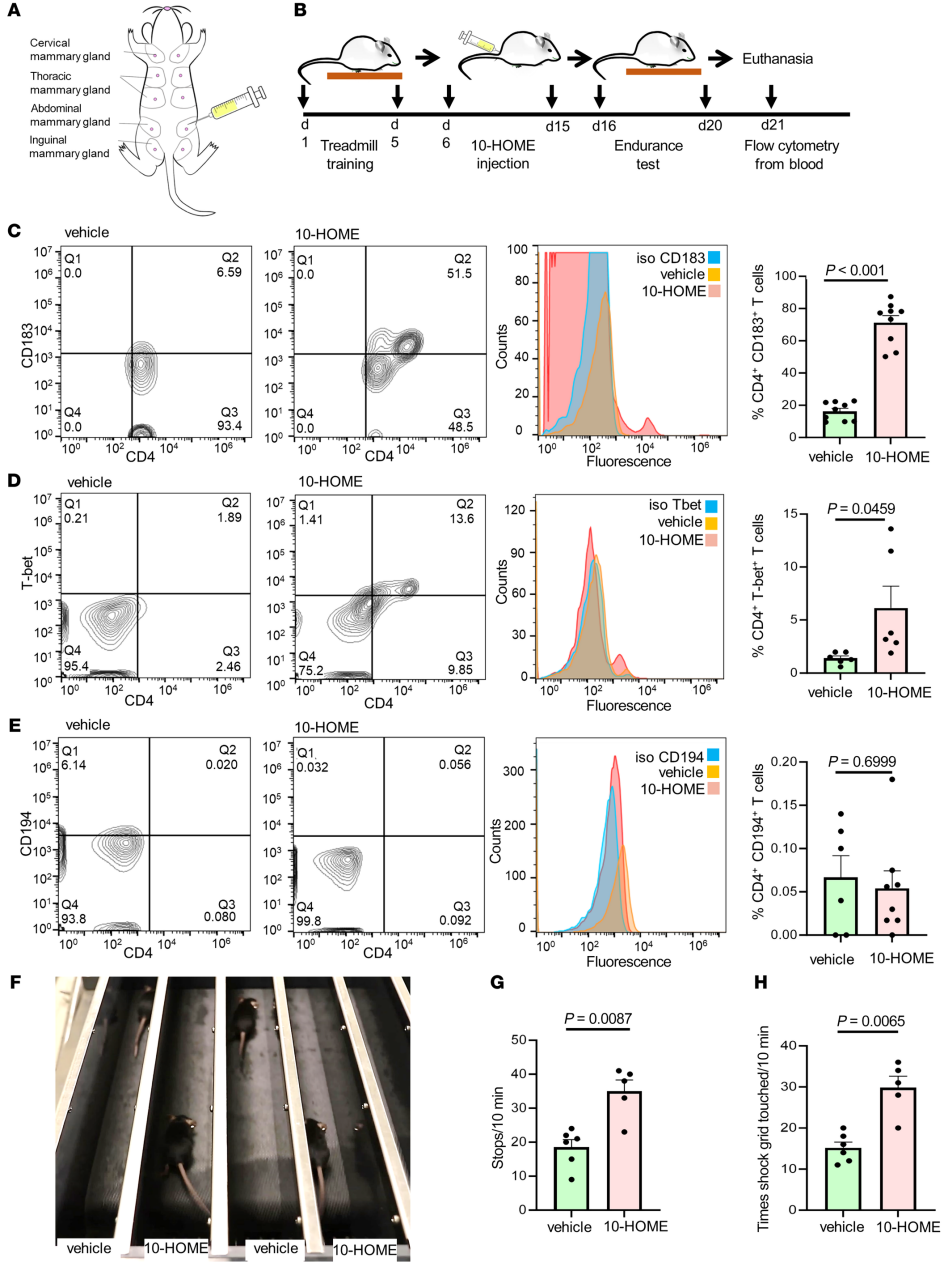
Elevated CD4^+^ Th1 and fatigue-like symptoms in mice administered with 10-HOME. (**A**) Schematic representation of injection of 10-HOME in the abdominal mammary fat pad of mice. (**B**) Timeline of 10-HOME administration in mice. (**C**) Elevated CD4^+^ Th1 subtype in the blood of mice injected with 10-HOME. Flow cytometry analyses of blood of mice stained with anti-CD4 (FITC) and anti-CD183 (PE). Representative flow plots: vehicle-treated, 10-HOME-treated, and histograms with isotype control for CD183. Data presented as mean ± SEM, vehicle (*n* = 9) and 10-HOME (*n* = 9) –treated mice. Wilcoxon-Rank test analysis was used to determine vehicle versus 10-HOME (*P* < 0.001). (**D**) Elevated CD4^+^ Th1 subtype in the blood of mice injected with 10-HOME. Flow cytometry analyses of blood of mice stained with anti-CD4 (FITC) and anti-Tbet (PE). Representative flow plots: vehicle-treated and 10-HOME–treated histograms with isotype control for T-bet. Data presented as mean ± SEM, vehicle (*n* = 6) and 10-HOME (*n* = 6) mice. *t* test was used to determine vehicle versus 10-HOME (*P* = 0.0459). (**E**) Unaltered CD4^+^ Th2 subtype in the blood of mice injected with 10-HOME. Flow cytometry analyses of blood of mice stained with anti-CD4 (FITC) and anti-CD194 (PE). Representative flow plots: vehicle-treated and 10-HOME–treated histograms with isotype control for CD194. Data presented as mean ± SEM, vehicle (*n* = 6) and 10-HOME (*n* = 8) –treated mice. *t* test was used to determine vehicle versus 10-HOME (*P* = 0.6999). (**F**) Representative image of murine endurance test after 10-HOME administration. Video provided as Supplemental Video 1. (**G**) Increased stops exhibited by 10-HOME administered mice compared with those treated with vehicle. Data presented as mean ± SEM, vehicle (*n* = 6) and 10-HOME (*n* = 5) –treated mice. Mann-Whitney *U* test with a Bonferroni correction was performed (*P* = 0.0087). (**H**) Increased aversive stimulation (shock grid touching) exhibited by mice administered 10-HOME compared with those treated with vehicle. Data presented as mean ± SEM, vehicle (*n* = 6) and 10-HOME (*n* = 5) –treated mice. Mann-Whitney *U* test with a Bonferroni correction was performed (*P* = 0.0065).

To evaluate if 10-HOME can induce Th1 cells in vivo, we administered 10-HOME into the abdominal mammary fat pad of C57BL/6J mice (Figure 6A). The timeline of in vivo murine model has been provided (Figure 6B). Two concentrations, a higher (6.5 mg/kg body weight for 10 days, based on existing oxylipin reports [ref. 52]) and a lower concentration (0.5 mg/kg for 30 days) of 10-HOME was administered at times specified into the abdominal mammary fat pad of C57BL/6J mice. An increased abundance of CD4^+^ Th1 (CD183^+^) cells was found in the murine blood in both protocols following administration of 10-HOME (Figure 6C and Supplemental Figures 19 and 20). In line with the observation, an increase of Tbet^+^ CD4^+^ cells were observed in the murine cohort administered with 10-HOME (Figure 6D and Supplemental Figures 19 and 20). Other subtypes of CD4^+^ T cells (Th2) (Figure 6E, Supplemental Figure 19A, and Supplemental Figure 20) and Th9 and Th22 (Supplemental Figure 19B and Supplemental Figure 20) were not statistically different. Similar administration of 10-HOME in ZsGreen mice with *Tbet/Tbx21* promoter (Tbet-ZsGreen reporter) led to increase of ZsGreen^+^ cells (Supplemental Figure 19, C and D). This implied activation of Th1-responsive *Tbet/Tbx21* promoter in presence of 10-HOME. To simulate the fatigue-like symptoms of women with BII, we assessed exercise tolerance of mice through endurance test using murine treadmill. Fatigue was quantified by 2 parameters: the number of times stopped and the number of instances that aversive stimulation (contact with shock grid) was required. Animals with 10-HOME exhibited increased stops (Figure 6, F and G, and Supplemental Video 1) and contact with shock grid (Figure 6H).

### CD4^+^ T cells in reaction with 10-HOME polarize macrophages to M1 phenotype.

**Figure 7:**
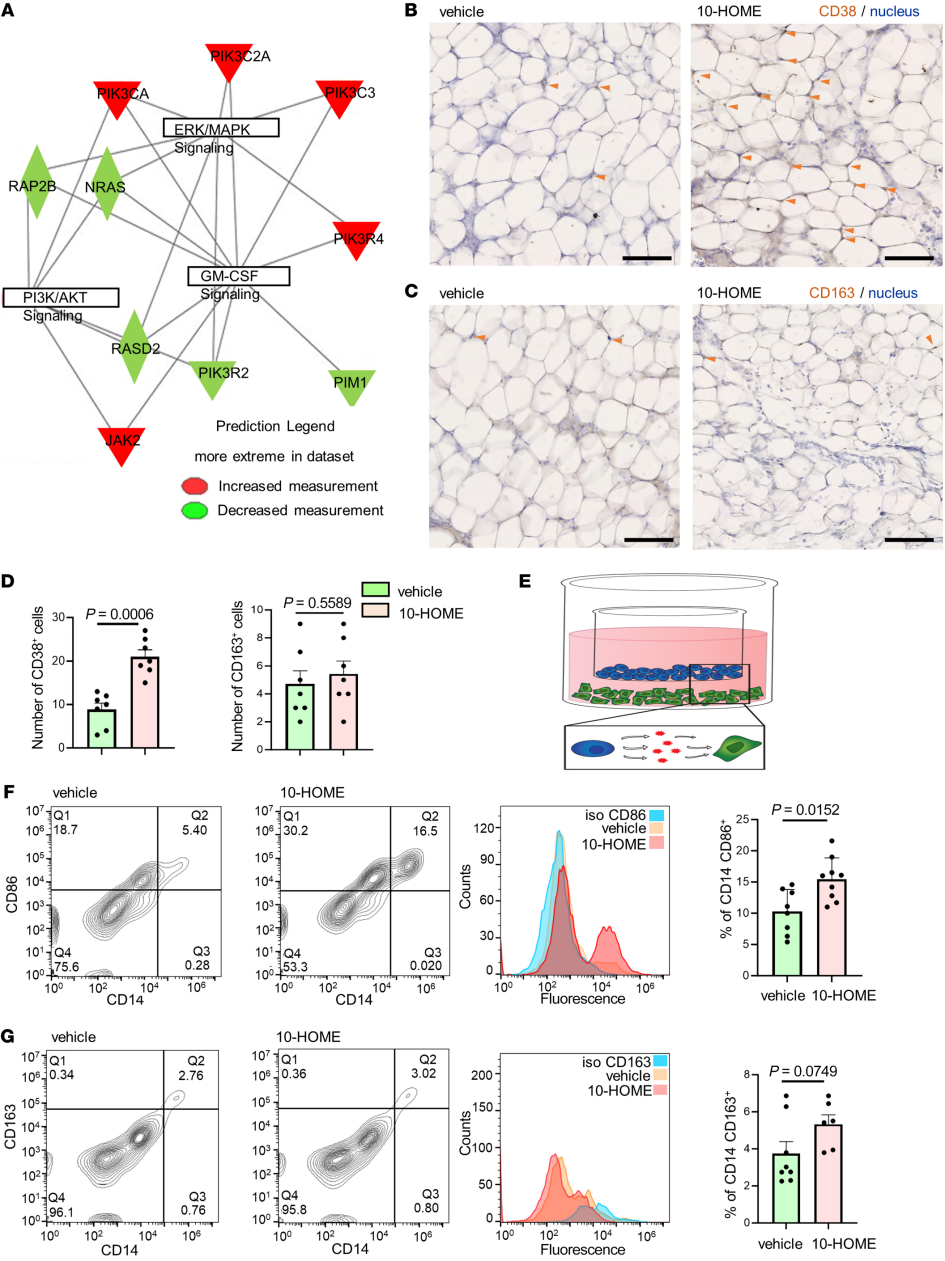
CD4^+^ T cells in reaction with 10-HOME polarize macrophages to M1 phenotype. (**A**) Gene interaction networks from human bulk RNA-Seq. Upregulated genes in red and downregulated are in green, in log_2_ fold change. Table exhibited in Supplemental Figure 21A. (**B**) Increased expression of CD38 (M1 macrophage marker) in 10-HOME–treated mice compared with vehicle. Murine mammary fat pads stained with anti-CD38 antibody (DAB) and nucleus (hematoxylin). The cells in adipose tissues are bordered by adipose cells. Cells coexpressing DAB and hematoxylin were considered for analysis (arrows). Data presented as mean ± SEM, vehicle (*n* = 7) and 10-HOME (*n* = 7) mice. Scale bar: 100μm. Enlarged image in Supplemental Figure 22. (**C**) No significant difference of CD163 (M2 macrophage marker) in 10-HOME treated mice. Murine mammary fat pads stained with anti-CD38 antibody (DAB). Cells co-expressing DAB and hematoxylin were considered for analysis (arrows). Data presented as mean ± SEM, vehicle (*n* = 7) and 10-HOME (*n* = 7) mice. Scale bar: 100μm. Enlarged image in Supplemental Figure 23. (**D**) Quantification of **B**–**C**. Total number of CD38^+^ and CD163^+^ cells were calculated. Data presented as mean ± SEM, (*n* = 7). Mann Whitney test was used to determine vehicle versus 10-HOME CD38 (*P* = 0.0006) and CD163 (*P* = 0.558). (**E**) Schematic representation of macrophage/T cell transwell assay. PBMC-derived naive T cells treated with 10-HOME (blue), PBMC-derived M0 macrophages (green). (**F**) Increased polarization to M1 phenotype (CD86-human M1 marker) in the transwell coculture of PBMC-derived M0 macrophages incubated with10-HOME–treated naive CD4^+^ T cells. Flow cytometry analyses with anti-CD86 (FITC) and anti-CD14 (PE). Representative plots: vehicle treated and 10-HOME treated, histograms of cells with isotype control for CD86. Data presented as mean ± SD, (*n* = 8). Mann Whitney test was used for comparison of vehicle versus 10-HOME (*P* = 0.0152). (**G**) Unaltered M2 phenotype(CD163). Flow cytometry analyses with anti-CD163 (APC) and anti-CD14 (PE). Representative plots, vehicle treated and 10-HOME-treated, histograms with isotype control for CD163. Data presented as mean ± SD, (*n* = 8). Mann Whitney test was used to determine vehicle versus 10-HOME (*P* = 0.0749).

There is a dynamic interaction of macrophages and T cells during antigen presentation (53, 54). Through an assessment of periprosthetic tissue by bulk RNA-Seq analyses, there was an increase of genes associated with M1 macrophage phenotype in tissues from the BII cohort compared with non-BII and normal tissues (Figure 7A and Supplemental Figure 21A). Assessment of the abdominal mammary fat pad from mice administered with 10-HOME showed an increased abundance of resident macrophages of M1 phenotype (Figure 7, B–D, and Supplemental Figures 22 and 23). In adipose tissue, adipose cells filled with lipid occupy most of the tissue. Other cells are present in borders of adipose cells. Cells costained with DAB (antibody) and hematoxylin (nucleus) were used for analyses of macrophage phenotype (Supplemental Methods). Other than macrophages, the 10-HOME–treated animal’s adipose tissue exhibited increased infiltration of cells, as observed through presence of more hematoxylin-positive nuclei that were not antibody stained. This was expected and in line with data presented for infiltration of T cells in the adipose tissue. To determine if the observation of M1 macrophage polarization is epiphenomenon or influenced by T cells in reaction to 10-HOME, we performed a trans-well assay. T cells pretreated with 10-HOME polarized PBMC–derived M0 macrophages to the M1 phenotype, suggesting a direct effect of T cells (Figure 7, E–G, and Supplemental Figure 21B).

## Discussion

Bacterial biofilms have been thought to cause gastric cancer (55), colon cancer (56), chronic inflammation, and BIA-ALCL (57, 58). The factors that involve interplay between host and pathogen are influenced by the microenvironmental niche where the bacteria reside (42, 59–61). Breast implants provided a conducive surface for the adherence and growth of bacterial biofilms (62). Many bacteria belonging to the normal microflora of the body have been reported to form bacterial biofilms (63). The observation in this study of increased abundance of bacterial biofilm comprising *Staphylococcus epidermidis* through NGS in implant-associated tissue from patients with BII relative to that of controls was thus critical in understanding of a potential etiology of BII. It is to be noted that while this study was being performed, anecdotal evidence of *S*. *epidermidis* with BII was reported by Lee et al. (64). *S*. *epidermidis* has been reported to be one of the main reasons for postsurgical implant failure and infection (31).

Implant material bioengineering, including that of breast implants, has substantially improved over time (65). There is a general perception of breast implant-associated complications like BIA-ALCL to be linked with textured implants (66). However, this study and others have recognized that BII-related immunological complications manifest irrespective of implant type (11, 64). This may be linked to the collagen capsule around the implant, which serves as the substratum for biofilm adherence and growth. Both smooth and textured implants form this capsule. The current study was not able to conclude any bias toward a specific type of implant.

The practice of capsulectomy for individuals diagnosed with BII in addition to implant removal further lends credence to the biofilm hypothesis (67). A recent report of 248 participants described better outcomes postcapsulectomy in patients with BII and reported an abundance of *Staphylococcus* *sp*. in their breast tissue (68).

Studies by us and others have reported the ability of bacteria to co-opt host lipids to form pathogenic biofilm (61, 69). The oxidation of fatty acids is one of the main biochemical reactions in the synthesis of lipid mediators. The oxygenation of unsaturated fatty acids leads to the formation of oxylipins. Although fatty acids are mostly found as triglycerides, the action of bacterial lipases result in the availability of free fatty acids. These fatty acids can then be oxidized by bacterial dioxygenases (DOX) and lipoxygenases (LOX) to form oxylipins. When oleic acid is used as a bacterial substrate, it is oxidized to 10-HOME. Notably, the adipose tissue found in the breast is rich in oleic acid containing lipids (70). The oxylipin 10-HOME has been reported to promote establishment of bacterial biofilms in vitro (48). The increased abundance of 10-HOME detected in our study associated with breast tissues of participants in the BII group thus suggests that breast microflora may interact with breast lipids, thus promoting the formation of bacterial biofilms. Oxylipins are also known to be immuno-modulatory. It has been reported that 12,13-DiHOME derived from oxidation of linoleic acid led to the reduction of regulatory T cells (Tregs), impeded immune tolerance, and promoted childhood atopy and asthma (49). This study reports that elevated levels of 10-HOME produced by bacterial biofilms led to immune cell activation as observed through in vitro and in vivo murine studies.

Immunological manifestations of BII are systemic. Implant-associated periprosthetic breast tissue from participants with BII revealed an increase of Th1 pathway activation–related transcripts via RNA-Seq. In support of our findings, a similar pathological activation of CD4^+^ Th 1 cells by microbial biofilm has been previously reported (71). CD4^+^ T cells play an important role in the pathogenesis of chronic systemic inflammatory autoimmune diseases such as multiple sclerosis, diabetes, and rheumatoid arthritis (72). Previous studies have shown *S*. *epidermidis* skewed T cell response toward a balance that allowed a stalemate between the host and the pathogen, in which the infection can become chronic (73–75). Oxylipins, such as prostaglandins and leukotrienes, had been extensively studied for their immunomodulatory effects (76). Prostaglandin E2 (PGE2) had been shown to promote the differentiation of naive CD4 T cells into Tregs and skew the immune response toward immunosuppression (77). On the other hand, leukotrienes have been associated with promoting inflammation and Th2-type responses (78, 79). Other oxylipins, such as lipoxins and resolvins, have been found to exert antiinflammatory and proresolving effects (80, 81). The effects of oxylipins on T cell skewing can be complex and context dependent. The presence of other cytokines, cell types, and the specific microenvironment could influence the outcome of T cell responses to oxylipin.

Oxidized lipids have been associated with pain and inflammatory conditions (82). Pain reported as arthralgia and myalgia is common in BII. We identified an increased presence of CD4^+^ Th1 cells in the breast tissue and peripheral blood of individuals with BII. Findings of this study demonstrate that 10-HOME could polarize naive CD4^+^ T cells toward Th1 subtype in vitro and in vivo. An increased abundance of the transcription factor Tbet, which is required for Th1 polarization, was identified after 10-HOME treatment. The polarization to the Th1 subtype was also supported by the observation of increased expression of proinflammatory cytokine IFN-γ, secreted by Th1 cells. Systematic analyses using mass cytometry revealed an increased abundance of Th1 subtype. The study did not show significant polarization toward Th2, Th9, Th17, Th22, and T-reg cell subtypes.

To correlate the association of 10-HOME with activation of CD4^+^ T cells in vivo, mice were administered with 2 different concentrations of 10-HOME in their mammary fat pad. An increase of CD4^+^ Th1 cells in peripheral circulation in 10-HOME–administered mice was observed in both cases. These observations help to explain the increased abundance of CD4^+^ Th1 cells in the participants in the BII group. Animals administered with 10-HOME exhibited fatigue-like symptoms as assessed using endurance test.

Taken together, we investigated the biofilm hypothesis of BII through a host-pathogen interaction. Implant-associated complications are poorly understood. These may be multifactorial. This work may be viewed as a first step in laying fundamental molecular mechanistic groundwork toward an understanding of a possible etiology for BII mediated via a host-biofilm interaction. The breast microenvironment led to formation of the biofilm-derived lipid metabolite 10-HOME from host oleic acid. The 10-HOME led to preferential activation of CD4^+^ Th1 cells in vitro and in vivo. The study provides the first evidence of a possible role of biofilm-derived 10-HOME inducing an immunological response in patients with BII. Halting the formation of biofilm-induced 10-HOME molecule could serve as possible therapeutic strategy to relieve patient symptoms in BII. Additionally, in light of the reports of biofilm association with metal implants such as orthopedic arthroplasty, this study provides a possible explanation of similar immunological responses reported in individuals with those implants (19–23). The findings of this study suggest that management of biofilm can help to increase the safety and long-term use of surgical implants. Further research needs to be conducted to elucidate if other biofilm-forming bacterial species are involved in the pathogenesis of BII. Also, the role of oxidized lipid products needs to be researched in a similar context. This study is an important step toward a mechanistic explanation of the multifactorial problem of BII, which is, at present, limited primarily to epidemiological studies with little research on molecular mechanisms; this research could open paths to therapeutic interventions.

## Methods

Methods and Statistical analyses are further detailed in the Supplemental Materials.

### ELISA.

Cell-free supernatants were collected and stored at −80°C. ELISAs for IFN-γ, IL4, and IL10 were performed using DuoSet kits (R&D Systems) per the manufacturer’s protocol.

### Bacterial strains.

*Staphylococcus epidermidis* (Winslow and Winslow), Evans (ATCC 35984), *Pseudomonas fluorescens* (ATCC 135925)*, Acinetobacter sp.* (ATCC 49139)*, Sphingomonas sp.* (ATCC 31461)*, Enterobacter cloacae* (ATCC 13047)*, Cutibacterium acnes* (ATCC 6919)*,* and *Cornyebacterium tuberculostearicum* (ATCC *35692),* were grown on tryptic soy agar plate at 37°C and thereafter were subcultured in M9 media supplemented with oleic acid 1% v/v for LC-MS/MS analyses.

### Quantitative real time PCR.

Breast tissue was pulverized using tissue pulverizer (6770 Freezer/Mill), and total RNA was extracted using miRVana (Thermo Fisher Scientific). cDNA was made using SuperScript III First-Strand Synthesis System (Invitrogen) or SuperScript VILO cDNA Synthesis Kit (Invitrogen). Quantitative or real-time PCR (Sybr Green) approach was used for mRNA quantification. Primer sequences used in this study are provided in Supplemental Table 4.

### 10-HOME administration in mice.

At 9 to 10 weeks of age, female mice were anesthetized with isoflurane. Two protocols (high and low dose) were followed. In the high dose protocol, 5 injections of 10-HOME (6.5 mg/kg body weight) were performed with a 27 G needle for 5 days to the abdominal mammary fat pad of mice. In the low dose protocol, 0.5 mg of 10-HOME/kg body weight was injected for 30 days to the abdominal mammary fat pad of mice. Blood was harvested for subsequent analyses.

### Participants.

Individuals participating in the study included 3 cohorts. The first group consisted of patients diagnosed with symptoms of BII. Two additional groups of individuals were evaluated as controls, patients who were implanted but are without symptoms of BII (non-BII) and nonimplanted breast tissue from participants (normal). Participants were diagnosed for BII using a clinical evaluation process that included a detailed medical history interview, a review of a comprehensive symptom inventory (Supplemental Table 1), and a physical examination as outlined in previous BII studies (7–13). Demographic characteristics of the patients are presented in Supplemental Table 2. All human studies and the participant questionnaire were approved by the Indiana University School of Medicine Institutional Review Board IRB# 2003674175. Declaration of Helsinki protocols was followed, and patients gave their written informed consent.

### Animals.

All animal (mice) experiments were approved by the Indiana University School of Medicine Institutional Animal Care and Use Committee (SoM-IACUC) under protocol 19102 and 22029 — *Murine model of breast implant diseases*. Animals were housed under a 12 hour light–dark cycle with food and water available ad libitum.

### Statistics.

The distribution of the increased abundance of Th subtypes were evaluated for normality using Shapiro-Wilk test and Q-Q plot. Descriptive statistics by groups (BII, non-BII, and normal) were calculated using mean (SD) for normally distributed data and median (interquartile range) for those deviating from normality. A 2-sample test of proportions with 2-tailed *z* tests were used to analyze the hypothesis that the proportion of different type of bacteria in the BII group was significantly different than the proportion of the biofilm infection in the non-BII and normal group. Nonparametric bivariate analyses were performed using the Kruskal-Wallis test followed by Dunn’s test for pairwise comparisons with Benjamini-Hochberg multiple testing adjusted *P* values to minimize the FDR. 2-tailed student *t* tests were used for the analysis of in vitro data, 10-HOME abundance in periprosthetic tissue between BII and non-BII participants. A *P* value less than 0.05 was considered statistically significant. 

### Data availability.

The RNA-Seq data have been deposited in the NCBI Gene Expression Omnibus (accession# GSE178425 for the periprosthetic breast tissue BII and non-BII tissues and GSE164641 for normal breast tissue). Supporting data values for bar and line graphs have been exhibited as supplemental Supporting Data Values file.

## Author contributions

MS, IK, and AHH conceived and designed the work. IK, REM, CKP, KS, LT, LJS, ER, BWV, CRN, GM, ML, RJV, RG, and NM participated in the data acquisition and analyses. MS, REM, IK, CKP, AHH, and MEK wrote the manuscript. MS, REM, CKS, GMG, MHK, and MEK reviewed the manuscript.

## Supplementary Material

Supplemental data

Supplemental video 1

Supporting data values

## Version 1 11/30/2023

In-Press Preview

## Version 2 02/01/2024

Electronic publication
