# Supplementary material for: Biofilm-derived oxylipin 10-HOME–mediated immune response in women with breast implants
Source: J Clin Invest. 2024 Feb 1;134(3):e165644. doi: 10.1172/JCI165644 (PMC10849761; doi:10.1172/JCI165644)
Supplement: Supplemental data [file jci-134-165644-s043.pdf]

Supplementary Material

Biofilm-derived oxylipin 10-HOME-mediated immune response in women with breast implants

Imran Khan[1], Robert E. Minto[2], Christine Kelley-Patteson[3], Kanhaiya Singh[1,4], Lava Timsina[1], Lily J. Suh[1], Ethan Rinne[1], Bruce W. Van Natta[3], Colby R. Neumann[1], Ganesh Mohan[1], Mary Lester[1], R Jason VonDerHaar[1], Rana German[5], Natascia Marino[5,6], Aladdin H. Hassanein[1], Gayle M. Gordillo[1,7], Mark H. Kaplan[8], Chandan K. Sen[1,4], Marshall E. Kadin[9,10], Mithun Sinha[1*]

[1]*Division of Plastic Surgery, Department of Surgery, Indiana University School of Medicine, Indianapolis, Indiana*

[2]*Department of Chemistry and Chemical Biology, Indiana University–Purdue University Indianapolis, Indianapolis, Indiana*

[3] *Meridian Plastic Surgeons, Indianapolis, Indiana*

[4]*McGowan Institute for Regenerative Medicine, Department of Surgery, University of Pittsburgh School of Medicine, Pittsburgh*

[5]*Susan G. Komen Tissue Bank at the IU Simon Comprehensive Cancer Center, Department of Medicine, Indiana University School of Medicine, Indianapolis, Indiana*

[6]*Division of Hematology & Oncology, Department of Medicine, Indiana University School of Medicine, Indianapolis, Indiana*

[7]*McGowan Institute for Regenerative Medicine, Department of Plastic Surgery, University of Pittsburgh School of Medicine, Pittsburgh*

[8]*Department of Microbiology and Immunology, Indiana University School of Medicine, Indianapolis, Indiana*

[9]*Department of Dermatology, Roger Williams Medical Center, Boston University School of Medicine, Providence, Rhode Island*

[10]*Department of Pathology, University of Virginia, Charlottesville, Virginia*

[*] Corresponding Author: Mithun Sinha, PhD; mitsinha@iu.edu

Address: 975 W Walnut St,
Medical Research Library Building, Suite # 444A
Indiana University School of Medicine
Indianapolis, IN 46202.
Tel. 317 278 2713
Fax. 317 278 2708

Supplementary Figures

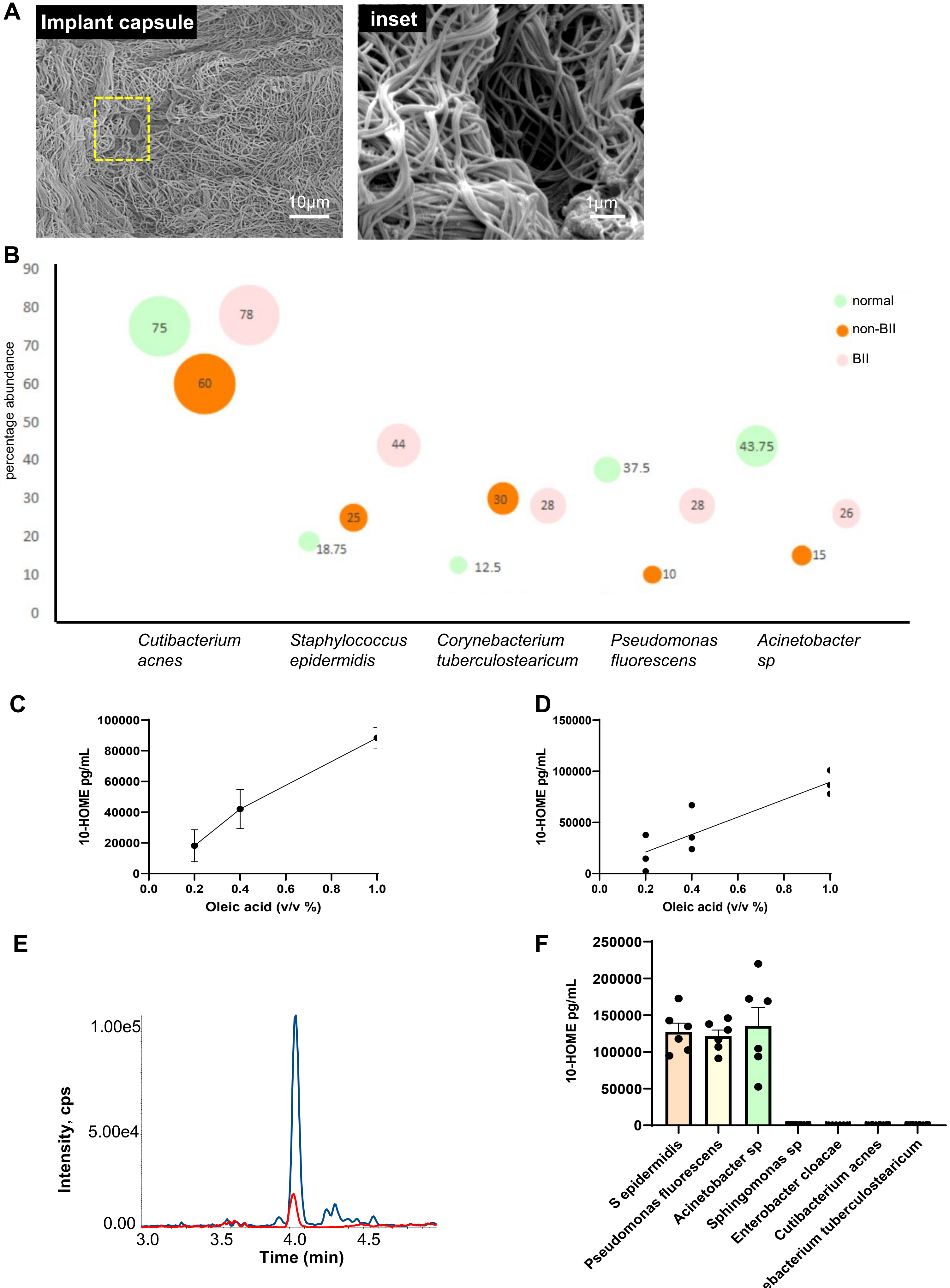

Supplementary Figure S1

Supplementary Fig S1

(A) Scanning electron microscopy and zoomed inset showing the collagen fibers of an implant-associated capsule from a non-BII individual. The individual didn't exhibit biofilm in the capsule.

(B) Bubble plot indicate the ranking of the top 5 bacteria associated with peri-prosthetic tissue with bubble size representing the rank for bacterial abundance in each group. The y-axis is the percentage of the bacterial abundance and the numbers within the bubbles indicate the percentage of each group by infection types and tells us if an individual with a group-type was selected, how likely will she be having a particular type of bacteria. n=16 (normal), n=20 (non-BII), n=50 (BII).

(C-D) Dose-response curve for production of 10-HOME by *S. epidermidis* grown for 12 h using oleic acid as a substrate. (C) Line graph (D) Line graph with individual data points.

(E) Ultra performance liquid chromatography chromatograms for 10-HOME synthetic standards (red), 10-HOME by *S. epidermidis in vitro* (blue).

(F) Production of 10-HOME in the supernatant of bacterial culture- *Staphylococcus epidermidis* (Winslow and Winslow) Evans (ATCC 35984) *Pseudomonas fluorescens* (ATCC 135925), *Acinetobacter sp* (ATCC 49139), *Sphingomonas sp* (ATCC 31461), *Enterobacter cloacae* (ATCC 13047), *Cutibacterium acnes* (ATCC 6919), *Corynebacterium tuberculostearicum* (ATCC 35692)

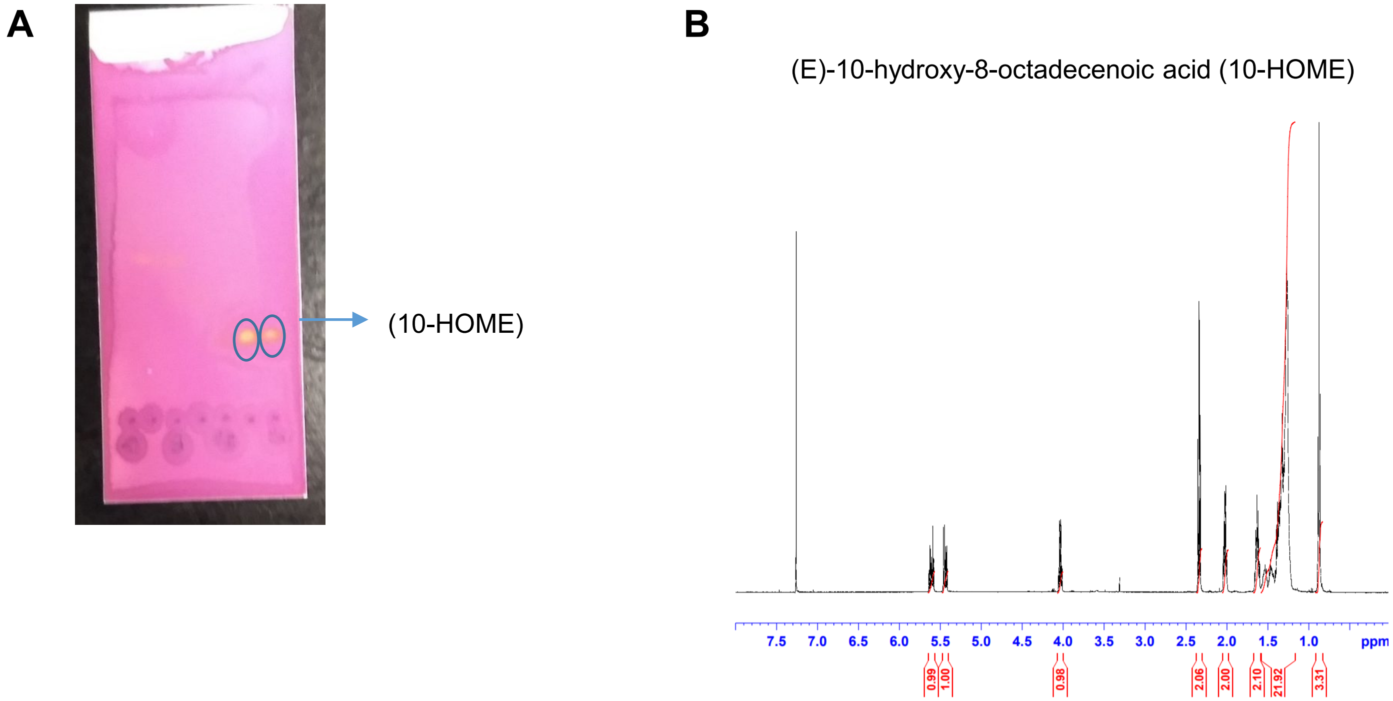

Supplementary Fig S2

(A) The synthesized 10-HOME internal standard was purified by silica-gel flash column chromatography as the methyl ester and analyzed by thin-layer chromatography (TLC). Fractions containing 10-HOME methyl ester (yellow spots were detected through permanganate staining) were collected and validated by gas chromatography-mass spectrometry (GC-MS). The standard was derivatized with *N,O*-bis(trimethylsilyl)trifluoroacetamide – 2% chlorotrimethylsilane (1h, 65°C) prior to analysis. GC-MS parameters: Agilent 7890/5975C system using a VF-23ms column (30m, 250 µm film, 0. 25 mm diameter) and an oven program starting at 60°C, ramp 7°C/min to 150°C, hold at 150°C for 7 min, ramp 10°C/min to 220°C, hold 1 min, ramp 50°C/min to 250°C, and hold 2 min; He flow 1.9 mL min; retention time 22 min. The parent ion and MS fragmentation was consistent with methyl 10-trimethylsilyl-8-octadecenoate. The Me ester was additionally characterized by proton and ^{13}C NMR spectroscopy. Purified 10-HOME methyl ester was then hydrolyzed to provide 10-HOME free acid.

(B) Validation of synthetic 10-HOME free acid standard using proton nuclear magnetic resonance (NMR) spectroscopy. Data was collected in $CDCl_3$ that had been deacidified by passage through a column of neutral alumina. Data was collected on a Bruker Avance II 500-MHz NMR spectrometer using a standard proton NMR pulse sequence. Proton-decoupled ^{13}C NMR data (not shown) further supported the identity and purity of the standard. All data was solvent referenced to δ 7.26 (^{1}H) and δ 77.0 ppm (^{13}C).

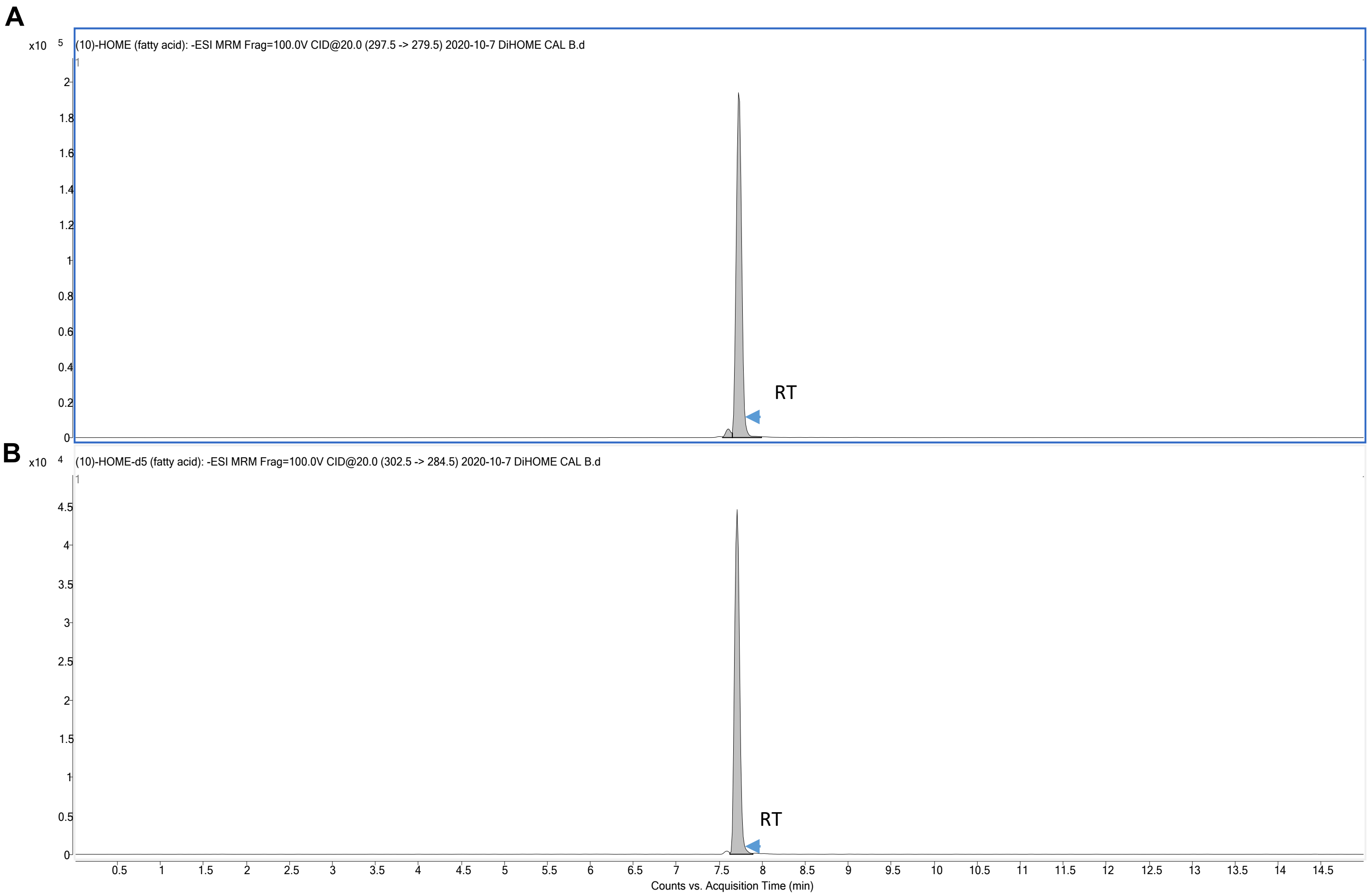

Supplementary Figure S3

(A) Ultra performance liquid chromatography chromatograms and selected mass spectra from 10-HOME synthetic standards. Panel A shows a chromatogram of 10-HOME.

(B) Panel B shows the corresponding chromatogram for 10-HOME-d_5.

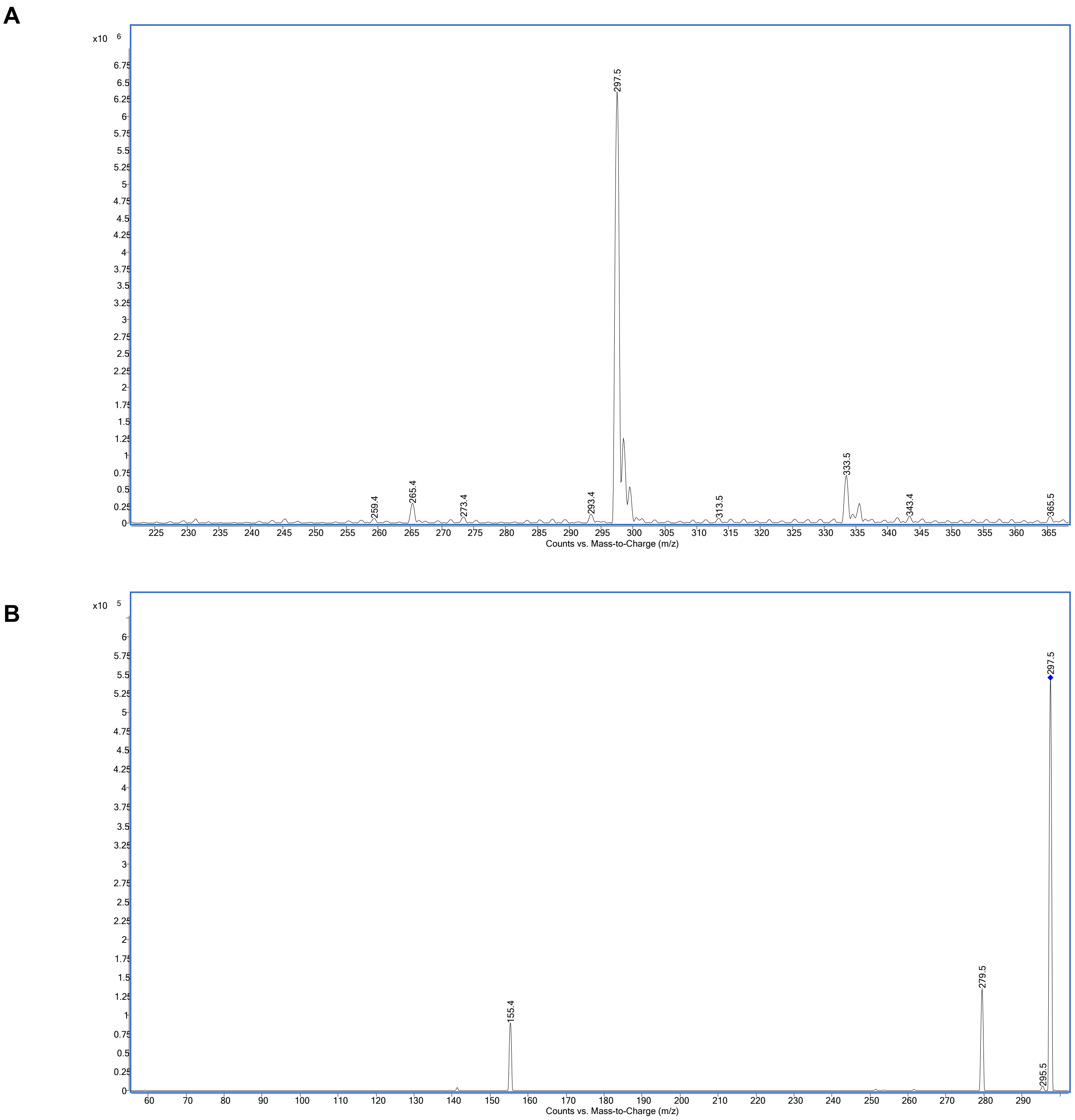

Supplementary Figure S4

(A) Electrospray LC-MS and (**B**) MS/MS product ion spectrum derived from the m/z 297.5 ion for 10-HOME

A

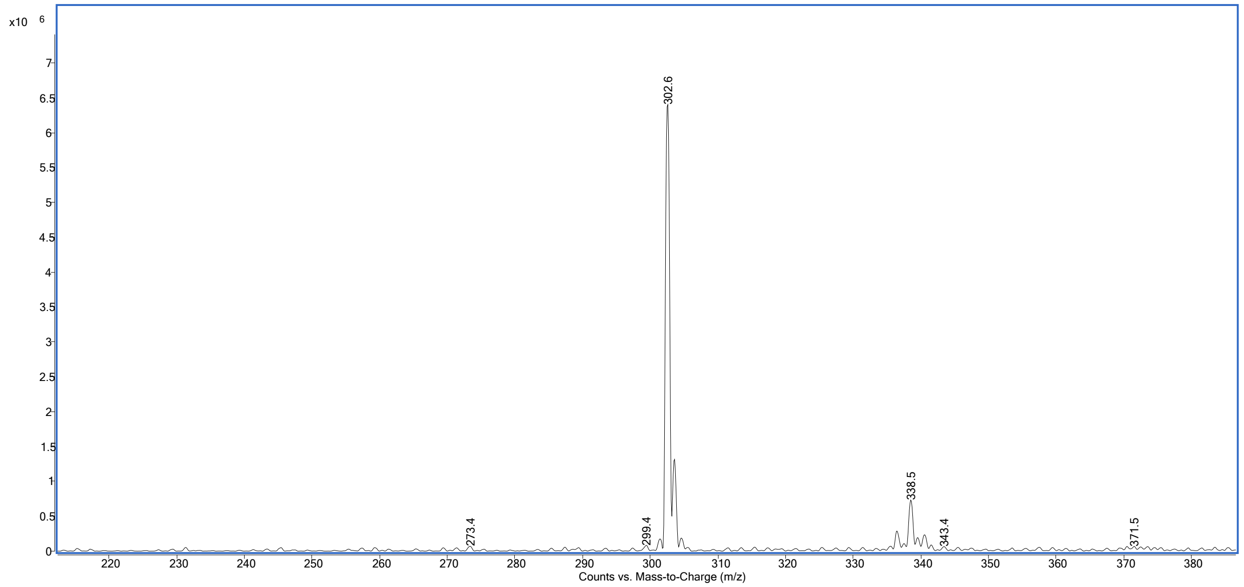

B

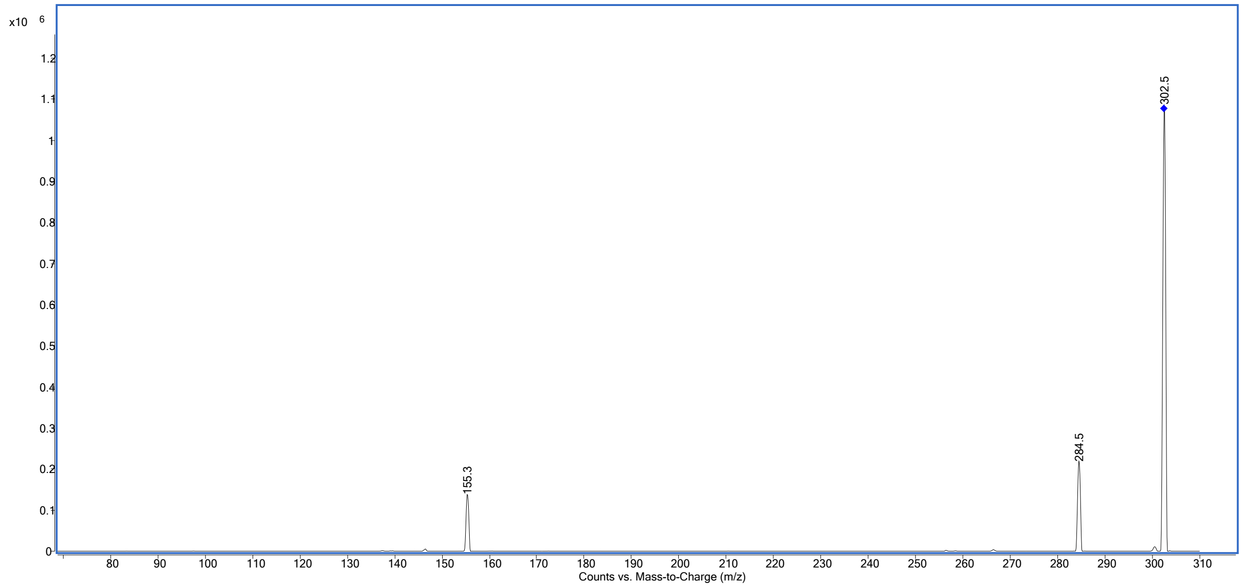

Supplementary Figure S5

(**A**)Electrospray LC-MS and (**B**) MS/MS product ion spectrum derived from the m/z 302.5 ion for 10-HOME-d_5

A

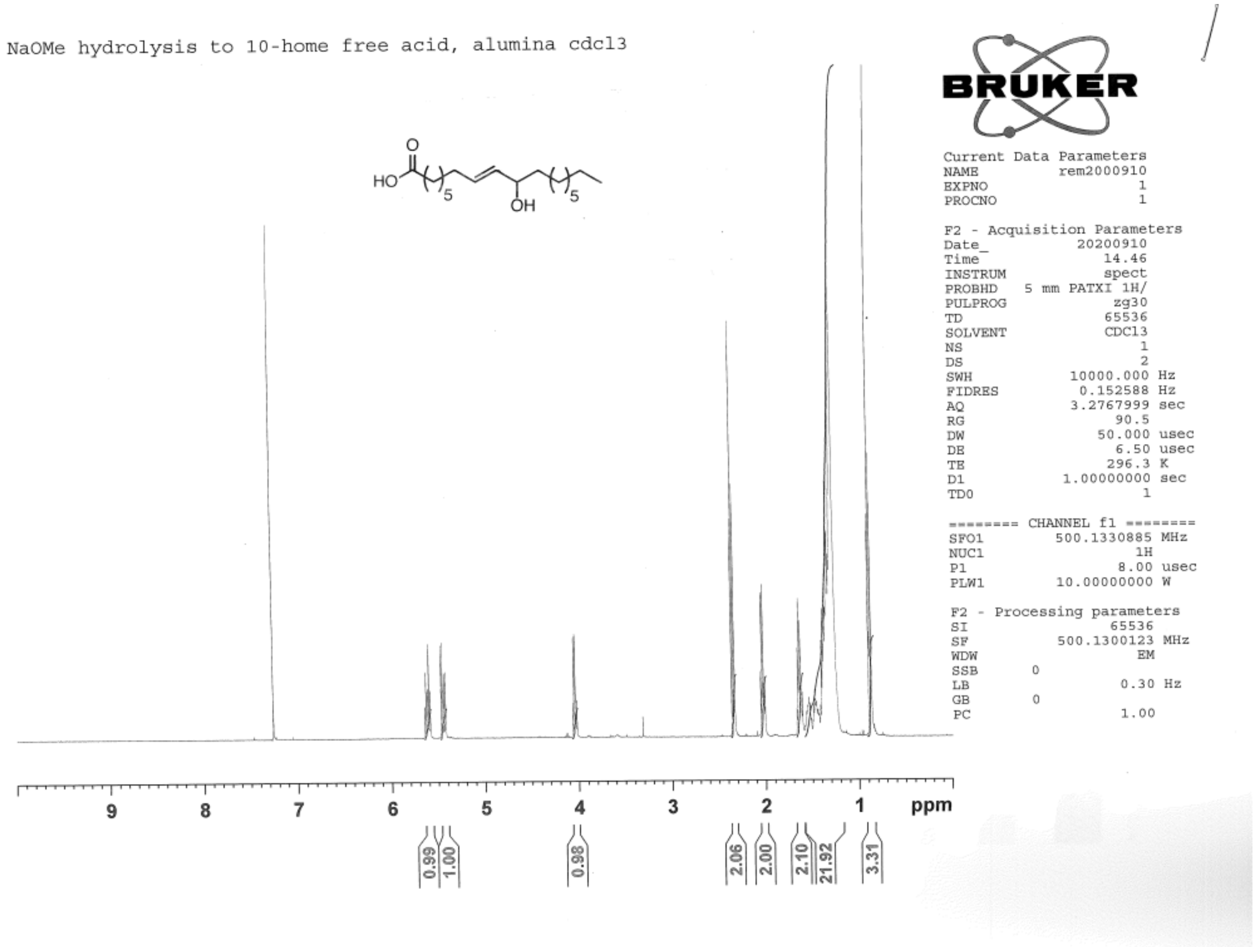

Supplementary Figure S6

(**A**) 500 MHz ^{1}H NMR spectra of 10-HOME in CDCl3. The 10-HOME was synthesized and purified as described in the method section.

A

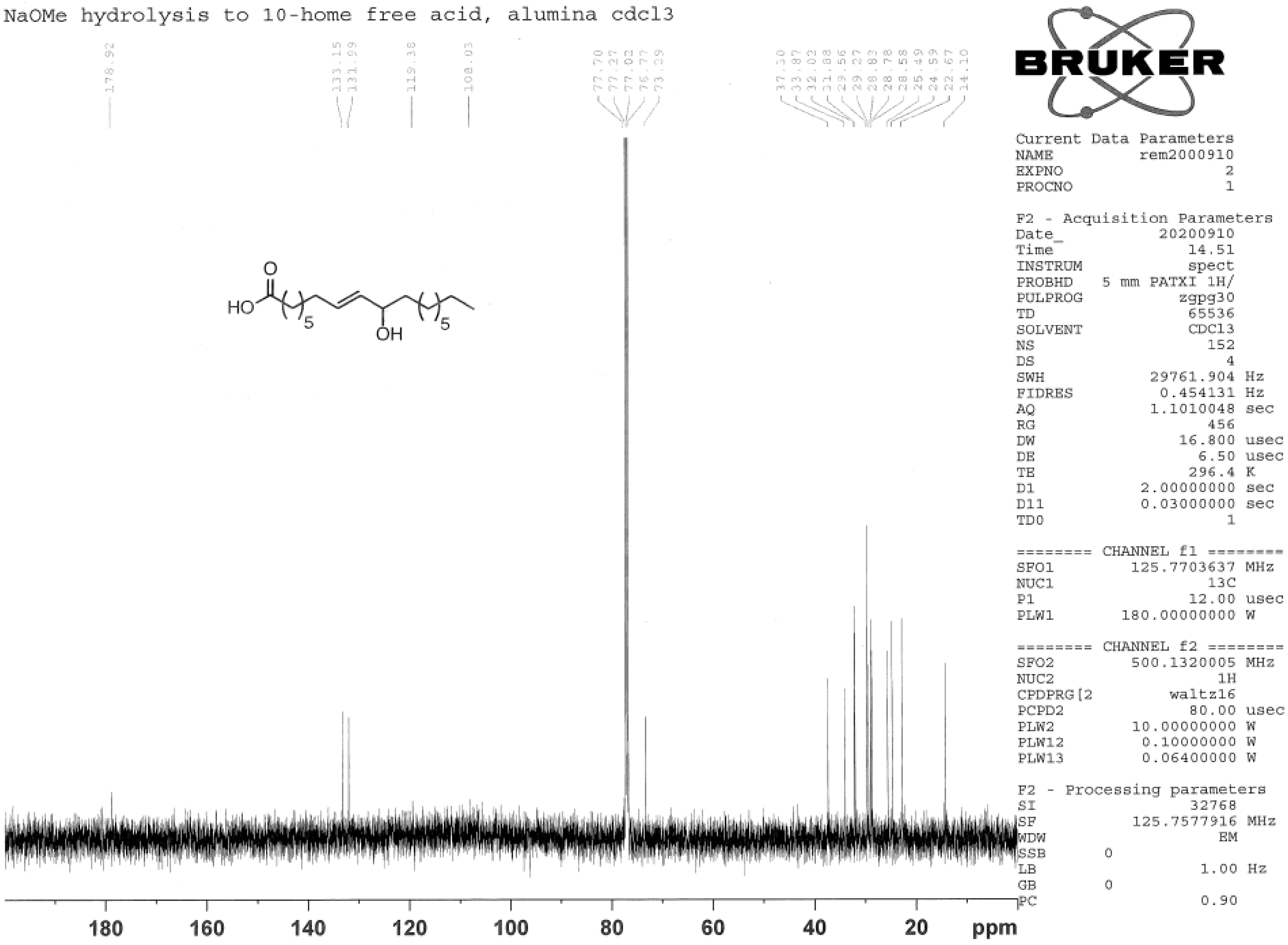

Supplementary Figure S7

(**A**) 500 MHz 13C NMR spectra of 10-HOME in CDCl3. The 10-HOME was synthesized and purified as described in the method section.

.

A

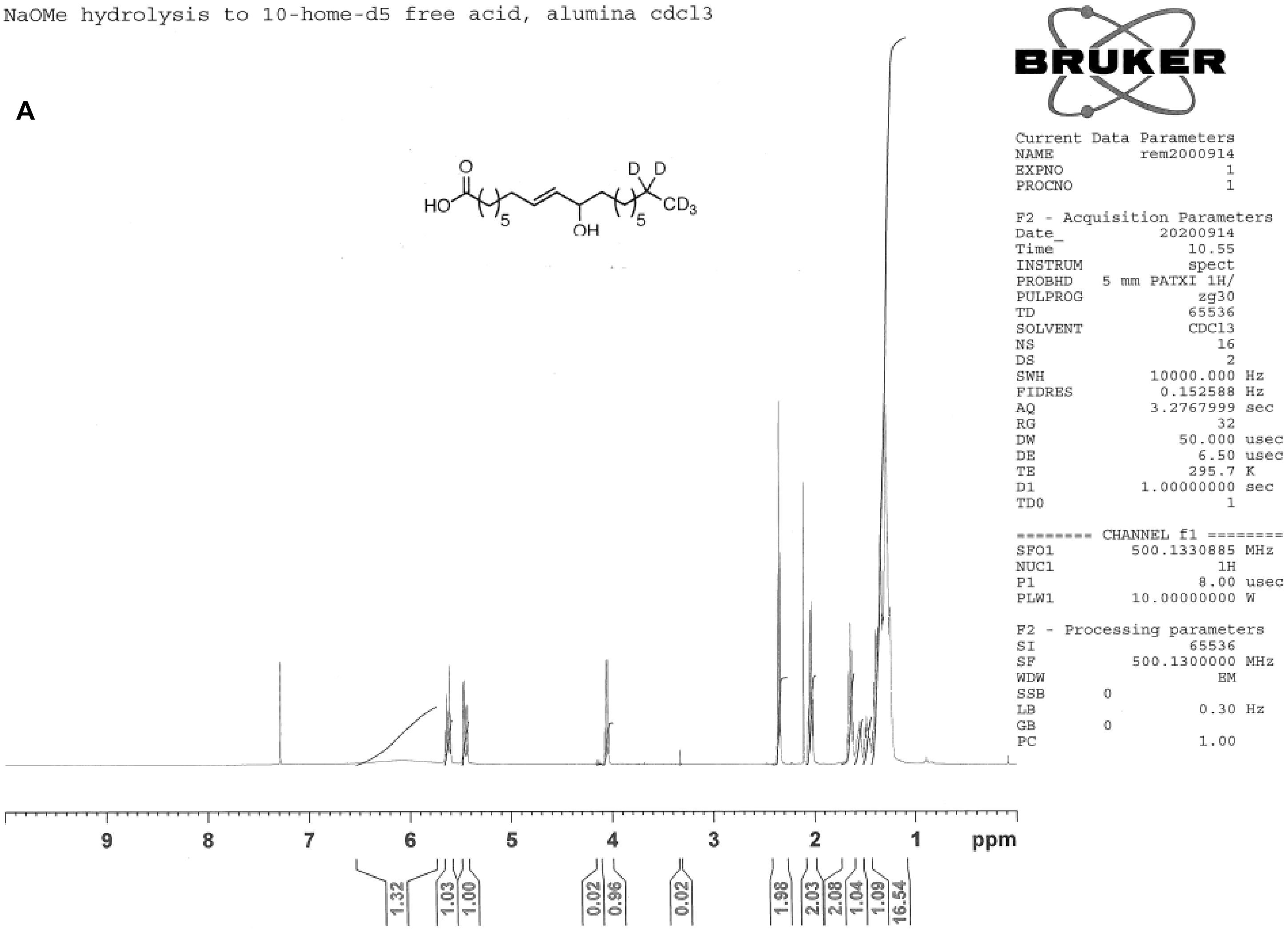

Supplementary Figure S8

(**A**) 500 MHz ^{1}H NMR spectra of 10-HOME-d_5 in $CDCl_3$. The 10-HOME was synthesized and purified as described in the Experimental Section.

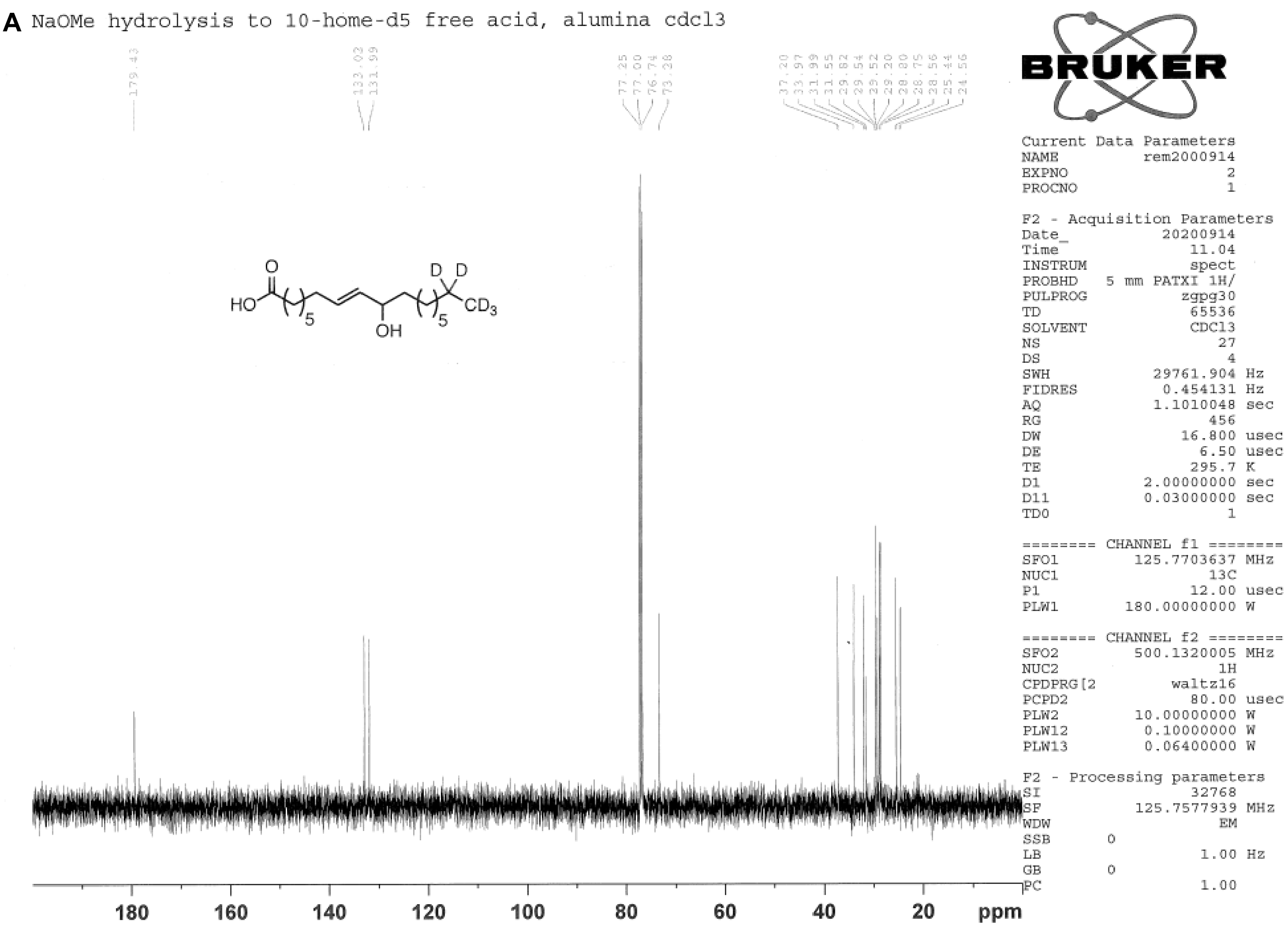

Supplementary Figure S9

(**A**)500 MHz ^{13}C NMR spectra of d5-10-HOME in CDCl3. The 10-HOME was synthesized and purified as described in the experimental section.

A

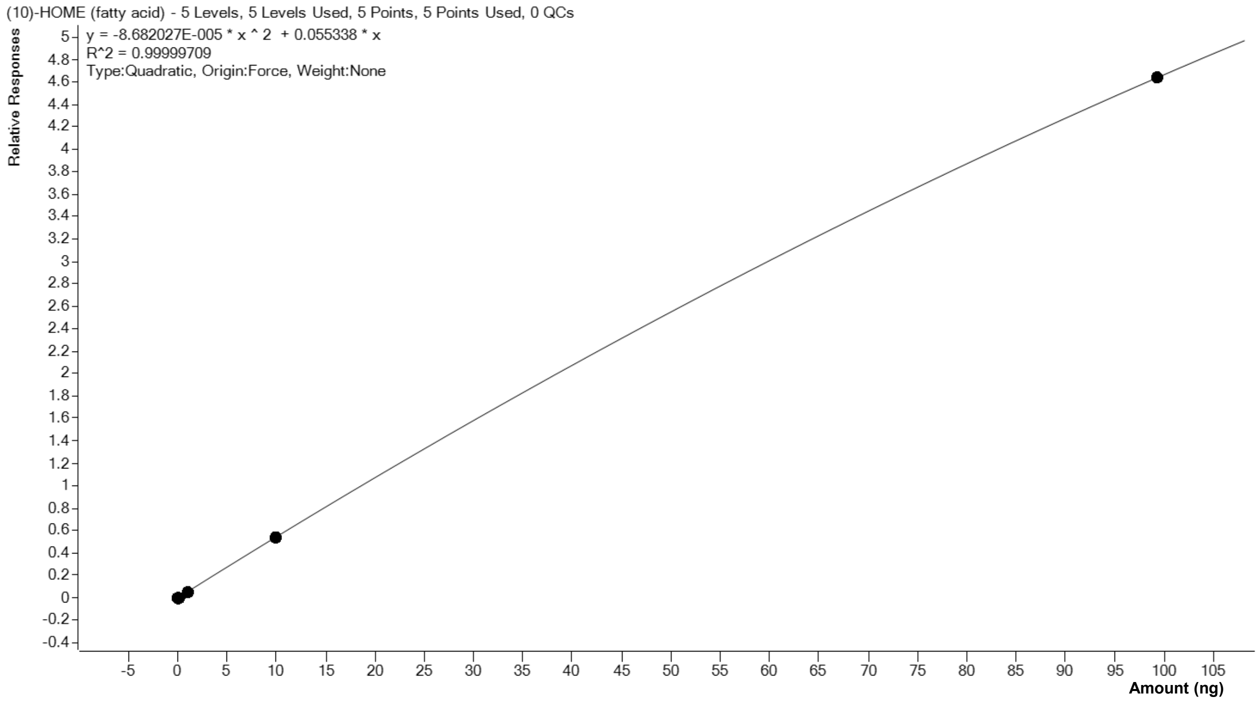

Supplementary Figure S10

(A) Calibration Curve of 10-HOME with 5 points 0.01, 0.1, 1, 10 and 100 ng.

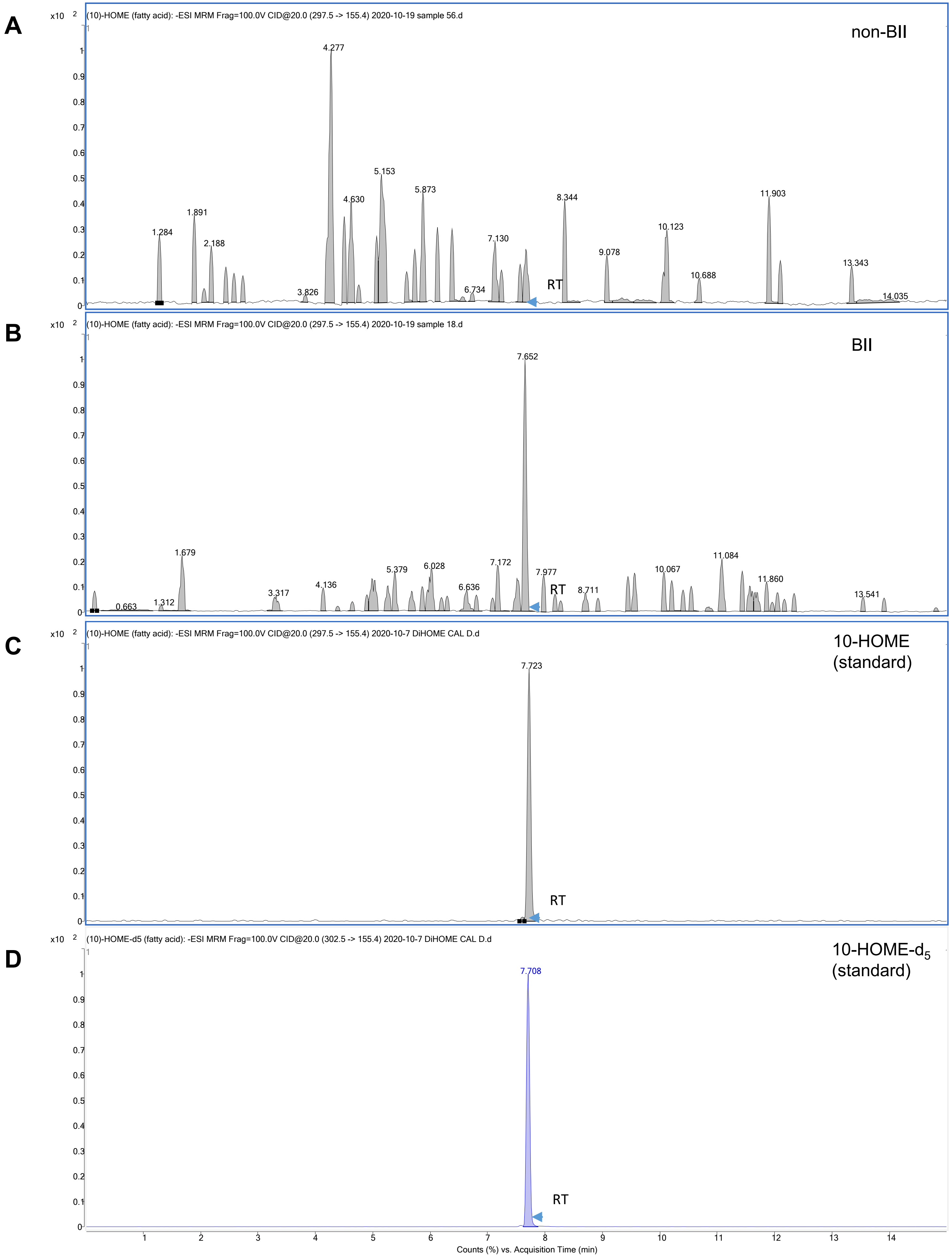

Supplementary Figure S11

Ultra performance liquid chromatography chromatograms of (A) non BII and (B) BII samples along with (C) 10-HOME (D) 10-HOME-d_5 synthetic standards.

A

Symbol	Entrez Gene Name	Experimental Log Ratio	Expected Trend
APH1A	aph-1 homolog A, gamma-secretase subunit	-0.134	Up
ICAM1	intercellular adhesion molecule 1	-0.653	
IFNGR1	interferon gamma receptor 1	0.169	Up
IRF1	interferon regulatory factor 1	-0.798	Down
JAK2	Janus kinase 2	0.304	Up
NFATC1	nuclear factor of activated T cells 1	-0.311	
NOTCH2	notch receptor 2	0.195	Up
PIK3C3	phosphatidylinositol 3-kinase catalytic subunit type 3	0.179	Up
PIK3C2A	phosphatidylinositol-4-phosphate 3-kinase catalytic subunit type 2 alpha	0.241	Up
PIK3CA	phosphatidylinositol-4,5-bisphosphate 3-kinase catalytic subunit alpha	0.294	Up
PIK3R2	phosphoinositide-3-kinase regulatory subunit 2	-0.263	Up
PIK3R4	phosphoinositide-3-kinase regulatory subunit 4	0.105	Up
PSENEN	presenilin enhancer, gamma-secretase subunit	-0.153	Up

C

Symbol	Entrez Gene Name	Experimental Log Ratio	Expected Trend
CD247	CD247 molecule	0.394	Up
CD8A	CD8a molecule	0.979	
HLA-DQB2	major histocompatibility complex, class II, DQ beta 2	0.918	
IFNA1/IFNA13	interferon alpha 1	0.979	Up
IFNAR1	interferon alpha and beta receptor subunit 1	0.552	Up
IL6	interleukin 6	0.711	Up
IL10RB	interleukin 10 receptor subunit beta	-0.969	
IL27RA	interleukin 27 receptor subunit alpha	0.836	Up
JAK1	Janus kinase 1	-0.996	Up
KLRD1	killer cell lectin like receptor D1	-0.757	
NCSTN	nicastrin	-0.572	Up
NFATC1	nuclear factor of activated T cells 1	-0.67	
NOTCH1	notch receptor 1	0.8	Up

B

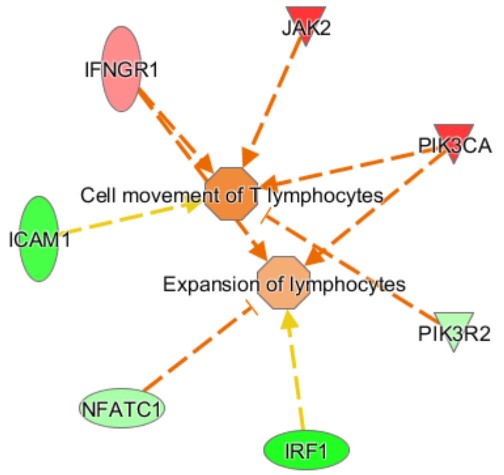

D

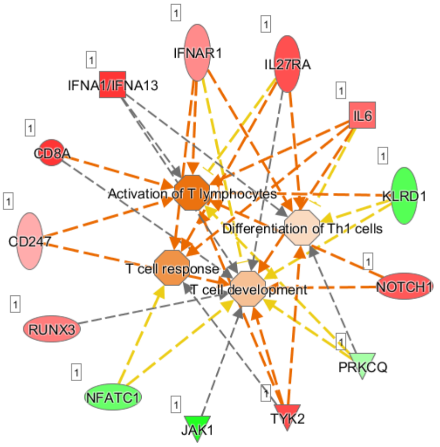

E

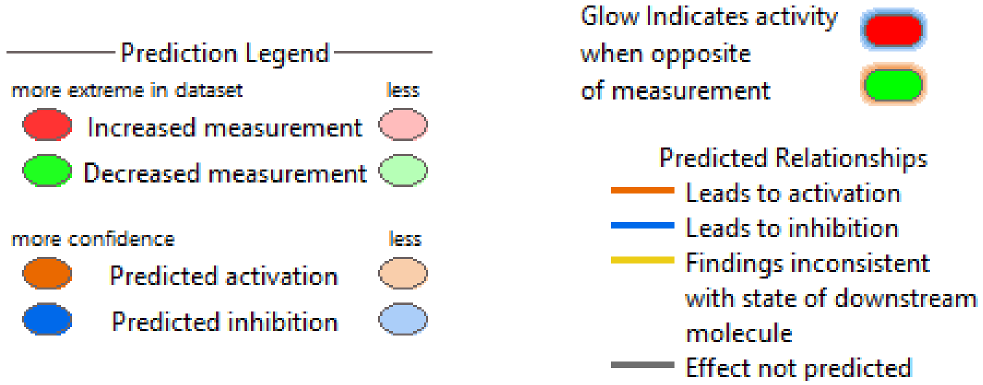

Supplementary Figure S12

Supplementary Fig S12

(**A**) List of the de-regulated gene signature in human Th1 cell population between BII *vs* non-BII from RNA-Seq results.

(**B**) Gene interaction networks for functions related to cell movement of T-lymphocytes, expansion of T-lymphocytes in BII specimens *vs* non-BII. Represented are extracted functional networks relevant to the set of imported genes generated by Canonical Pathway function relevant to Th1 pathway. The list was selected from the hierarchical cluster of Th1 genes, that were upregulated are shown in red and downregulated in green. The intensity of red and green corresponds to an increase and decrease, respectively, in Log2 fold change. Pathway analysis was performed using Ingenuity Pathway Analysis™ (IPA; Qiagen, Inc., USA) software.

(**C**) List of the de-regulated gene signature in human Th1 cell population between BII *vs* normal from RNA-Seq results.

(**D**) Gene interaction networks for functions related to T- cell development, and response, activation of T-lymphocytes, Immune response signaling for differentiation of Th1-lymphocytes in BII specimens. The list was selected from the hierarchical cluster of Th1 genes that were significant when compared to normal tissue. Genes that were upregulated are shown in red and downregulated in green. The intensity of red and green corresponds to an increase and decrease, respectively, in Log2 fold change.

(**E**) Legend indicating color codes for the gene networks depicted in B & D.

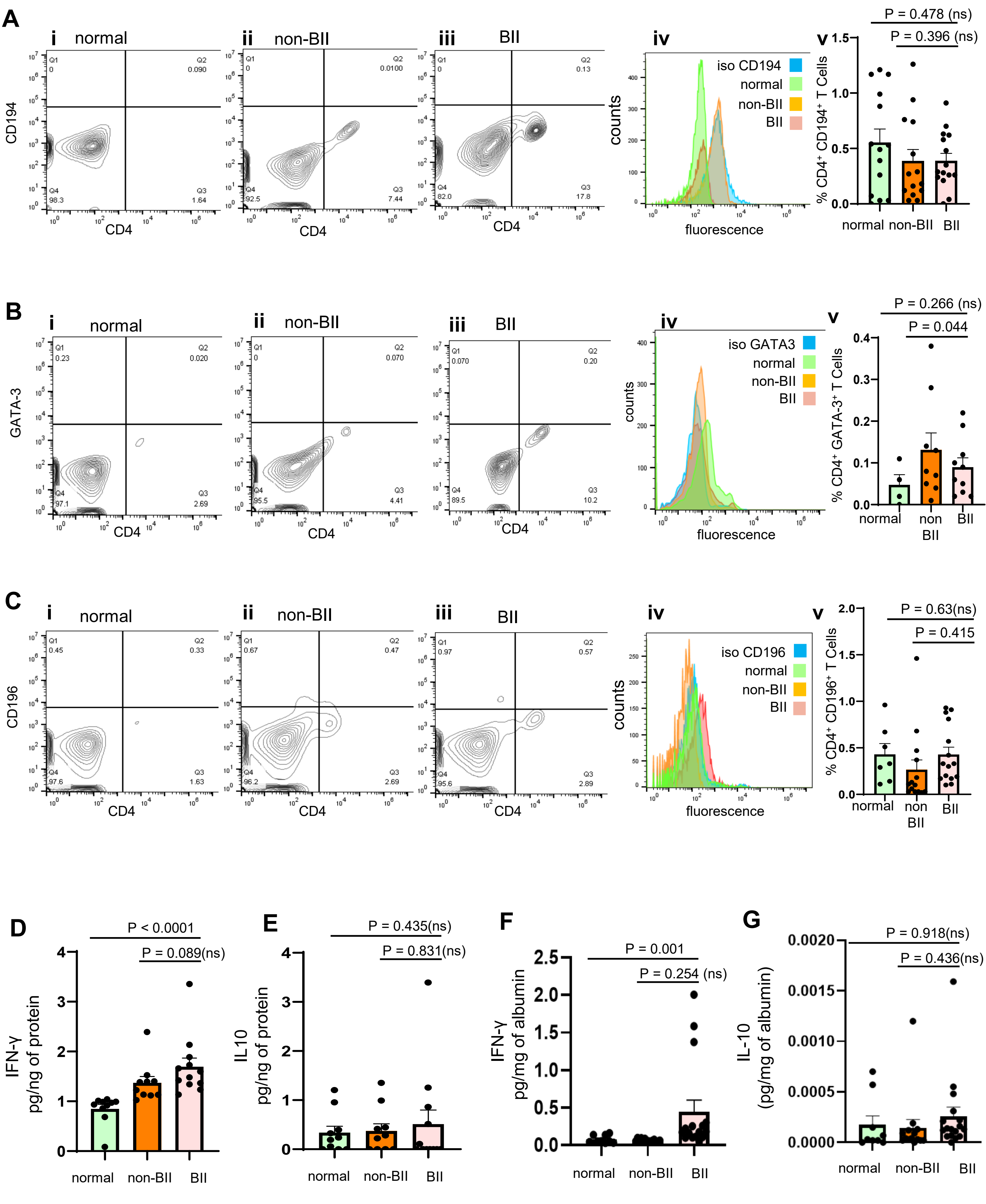

Supplementary Figure S13

Supplementary Fig S13

(A) Flow cytometry analyses of peripheral blood of participants stained with anti-CD4 (FITC) and anti-CD194 (PE). Representative flow plots. (i) normal (ii) non-BII (iii) BII (iv) histogram with isotype control for CD194 (v) % of $CD4^+$ $CD194^+$ T cells. Data presented as mean ± SEM, n= 14 (normal), n=14 (non-BII), n=15 (BII). Bivariate Kruskal-Wallis with post-hoc Benjamini-Hochberg adjusted pairwise comparison analysis BII *vs* normal (P = 0.478); BII *vs* non-BII (P = 0.396).

(B) Flow cytometry analyses of peripheral blood of participants stained with anti-CD4 (FITC) and anti-GATA-3 (PE). Representative flow plots. (i) normal (ii) non-BII (iii) BII (iv) histogram with isotype control for GATA-3 (v) % of $CD4^+$ GATA-3^+ T cells. Data presented as mean ± SEM, n= 4 (normal), n=9 (non-BII), n=10 (BII). Bivariate Kruskal-Wallis with post-hoc Benjamini-Hochberg adjusted pairwise comparison analysis BII *vs* normal (P = 0.266); BII *vs* non-BII (P = 0.044).

(C) Flow cytometry analyses of peripheral blood of participants stained with anti-CD4 (FITC) and anti-CD196 (PE). Representative flow plots. (i) normal (ii) non-BII (iii) BII (iv) histogram with isotype control for CD196 (v) % of $CD4^+$ $CD196^+$ T cells. Data presented as mean ± SEM n= 7 (normal), n=14 (non-BII), n=15(BII). Bivariate Kruskal-Wallis with post-hoc Benjamini-Hochberg adjusted pairwise comparison analysis BII *vs* normal (P = 0.630); BII *vs* non-BII (P = 0.415).

(D) Elevated Th1 cytokine IFN-ϒ (Th1 cytokine) in the periprosthetic breast tissue of BII participants compared to that of normal individuals as measured through ELISA and normalized with serum albumin. Data presented as mean ± SEM, n=10 (normal), n=10 (non-BII), n=12 (BII). Bivariate Kruskal-Wallis with post-hoc Benjamini-Hochberg adjusted pairwise comparison analysis BII *vs* normal (P < 0.0001); BII *vs* non-BII (P = 0.089)

(E) No significant change in the levels of IL10 (Th2 cytokine) in the periprosthetic breast tissue of BII participants compared to that of non-BII and normal participants as measured through ELISA and normalized with serum albumin. Data presented as mean ± SEM, n=10 (normal), n=10 (non-BII), n=12 (BII). Bivariate Kruskal-Wallis with post-hoc Benjamini-Hochberg adjusted pairwise comparison analysis BII *vs* normal (P = 0.435); BII *vs* non-BII (P = 0.831)

(F) Elevated Th1 cytokine IFN-ϒ (Th1 cytokine) in the blood of BII participants compared to that of normal participants as measured through ELISA and normalized with serum albumin. Data presented as mean ± SEM, n=9 (normal), n=14 (non-BII), n=16 (BII). Bivariate Kruskal-Wallis with post-hoc Benjamini-Hochberg adjusted pairwise comparison analysis BII *vs* normal (P = 0.001); BII *vs* non-BII (P = 0.254)

(G) No significant change in the levels of IL10 (Th2 cytokine) in the blood of BII participants compared to that of non-BII and normal participants as measured through ELISA and normalized with serum albumin. Data presented as mean ± SEM, n=9 (normal), n=14 (non-BII), n=17 (BII). Bivariate Kruskal-Wallis with post-hoc Benjamini-Hochberg adjusted pairwise comparison analysis BII *vs* normal (P = 0.918); BII *vs* non-BII (P = 0.436)

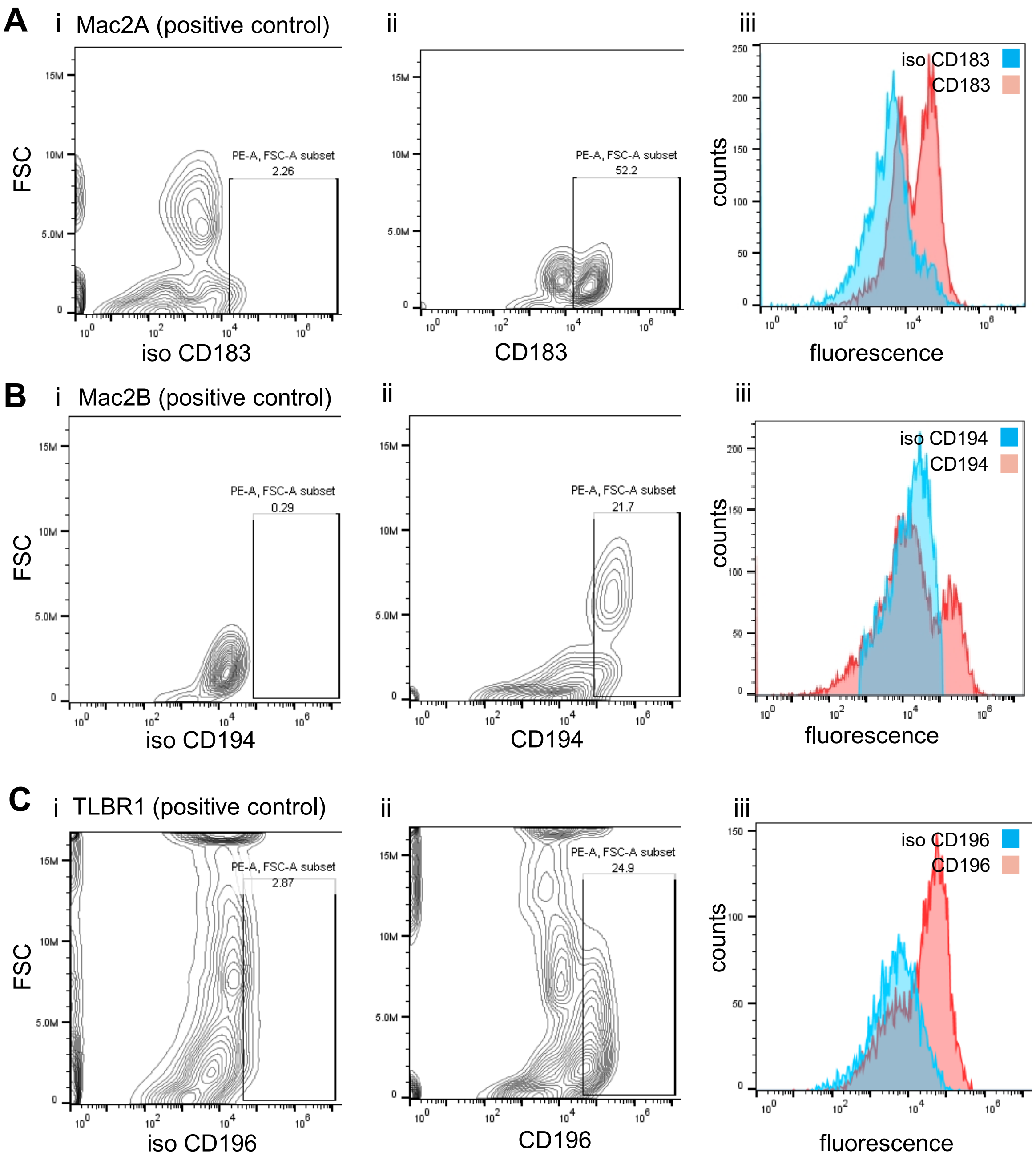

Supplementary Fig S14

(A) Flow cytometry of CD4+ Mac 2A cell line (positive control) stained with isotype control to CD 183 or anti-CD183. Representative flow plots. (i) isotype control (ii) CD183 (iii) histogram with isotype control for CD183.

(B) Flow cytometry of CD4+ Mac 2B cell line (positive control) stained with isotype control to CD 194 or anti-CD194. Representative flow plots. (i) isotype control (ii) CD194 (iii) histogram with isotype control for CD194.

(C) Flow cytometry of CD4+ TLBR1 cell line (positive control) stained with isotype control CD 196 or anti-CD196. Representative flow plots. (i) isotype control (ii) CD196 (iii) histogram with isotype control for CD196.

Supplementary Figure S14

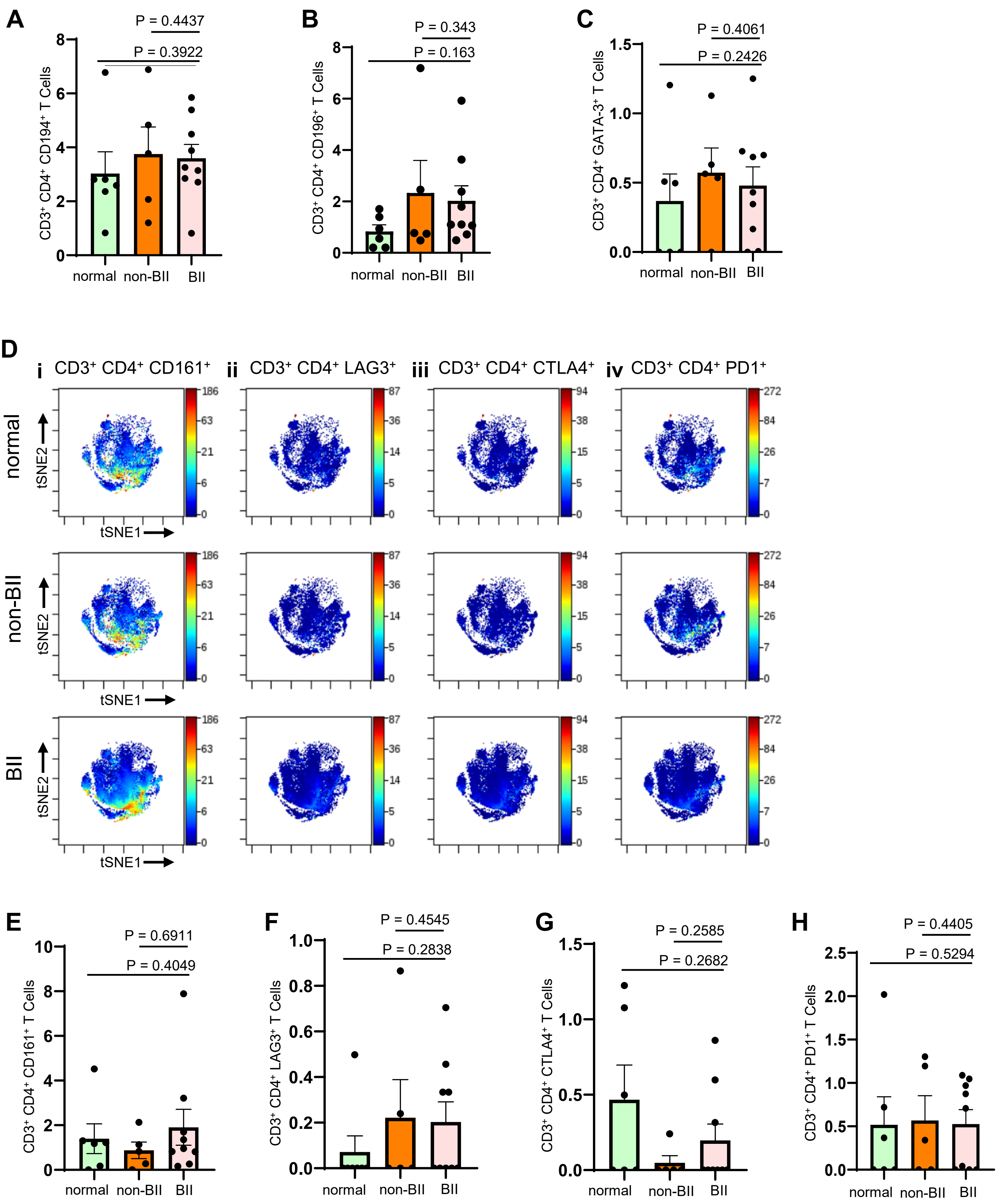

Supplementary Figure S15

Supplementary Fig S15

(**A-C**) Quantification of median marker expression using CytoBank software for **Figure 4E** (iii-v) (**A**) CD194, (**B**) CD196 and (**C**)GATA3 for individual data points for participants. n=6 (normal), n=5 (non-BII), n=9 (BII). Bivariate Kruskal-Wallis with post-hoc Benjamini-Hochberg adjusted pairwise comparison analysis. (**A**) For CD194 BII *vs* normal (P = 0.3922); BII *vs* non-BII (P = 0.4437). (**B**) For CD196 BII *vs* normal (P = 0.163); BII *vs* non-BII (P = 0.343). (**C**) For GATA3 BII *vs* normal (P = 0.2426); BII *vs* non-BII (P = 0.4061).

(D) Representative viSNE plots for $CD3^+$ $CD4^+$ T cells with Th markers depicting tumorigenic subtype (i-v). For each plot, the color depicts the intensity of the marker labeled on arcsinh scales ranging from blue (low) to red (high). The t-SNE analyses indicated no statistical difference in the abundance of CD161, LAG3, CTLA4 and PD1 markers in the peripheral blood of BII participants compared to that of non-BII and normal cohorts.

(**E-H**) Quantification of median marker expression using CytoBank software in panel **D** for (**E**) CD161, (**F**) LAG3, (**G**) CTLA4 and (**H**) PD1 for individual data points for participants. n=6 (normal), n=5 (non-BII), n=9 (BII). Bivariate Kruskal-Wallis with post-hoc Benjamini-Hochberg adjusted pairwise comparison analysis. (**E**) For CD161 BII *vs* normal (P = 0.4049); BII *vs* non-BII (P = 0.6911). (**F**) For LAG3 BII *vs* normal (P = 0.2838); BII *vs* non-BII (P = 0.4545). (**G**) For CTLA4 BII vs normal (P = 0.2682); BII *vs* non-BII (P = 0.2585). (**H**) For PD1 BII *vs* normal (P = 0.5294); BII *vs* non-BII (P = 0.4405).

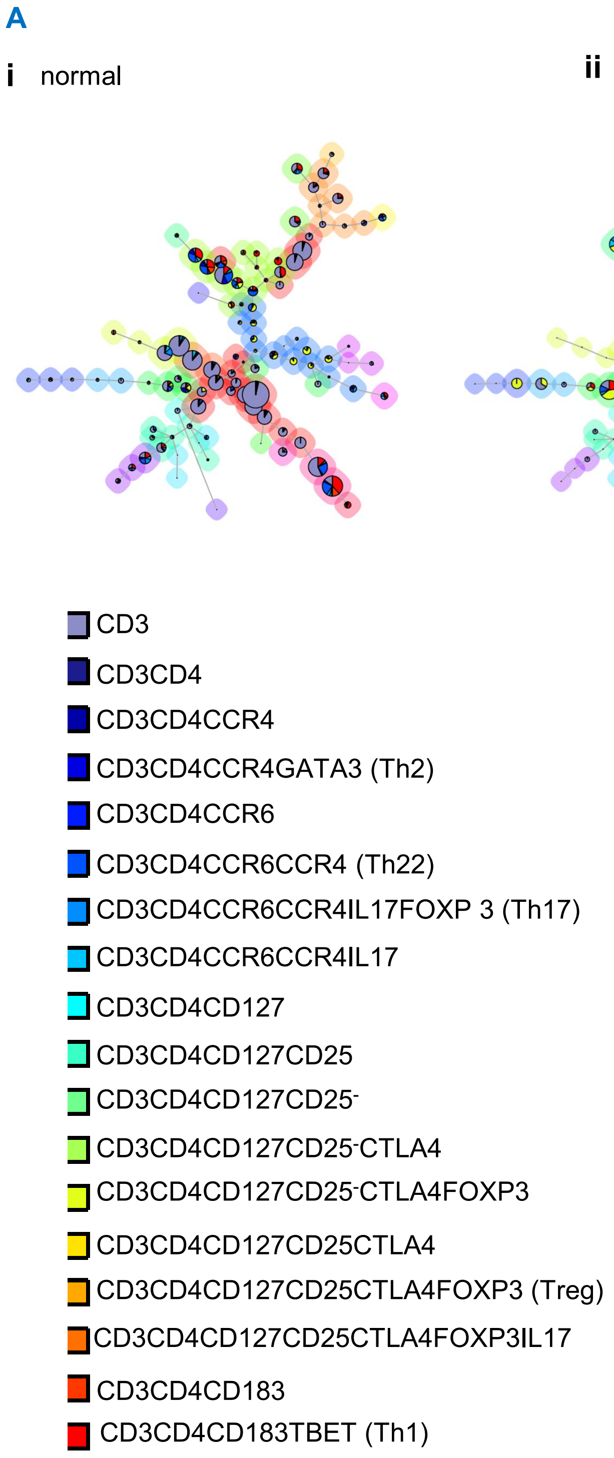

Supplementary Figure S16

(**A**) FlowSOM minimal spanning tree (MST) clustering identifies T -cell subsets. Total T cell CYTOF data from (i)normal, (ii)non BII, (iii) BII were clustered into 8 nodes with subsequent automated meta-clustering into 25 T cells subsets (meta-clusters) using clustering channels and the FlowSOM algorithm. The relationships between the nodes (which are most like each other) are shown by the spanning tree with similar nodes placed close together on the plot. Expression intensity of each clustering marker on the same spanning tree plot is shown in the three diagrams Dark red represents maximum expression, and dark blue represents no expression of the given marker as shown in the colour bar. A coloured halo around the node indicates the meta-cluster to which each node belongs.

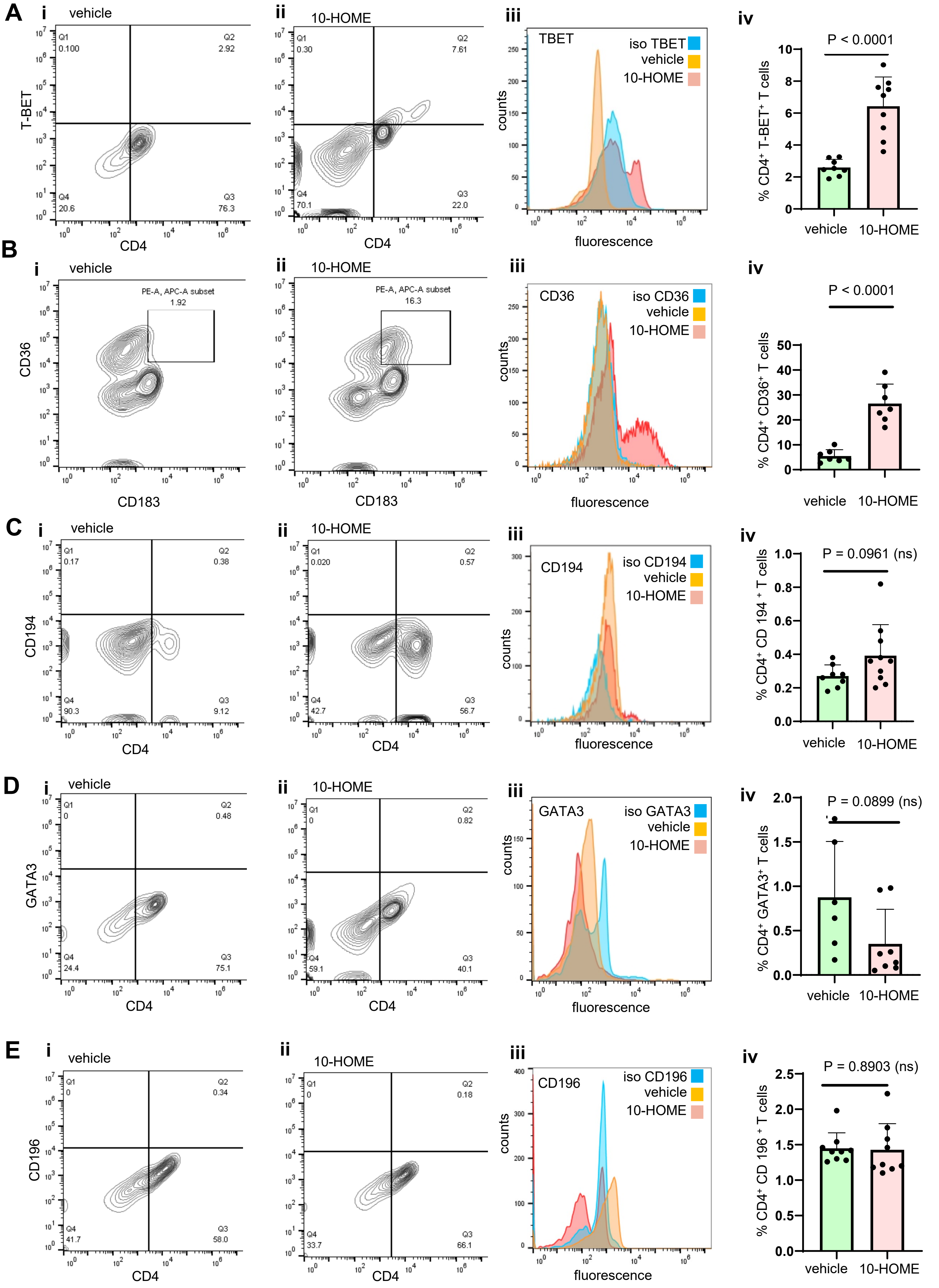

Supplementary Figure S17

Supplementary Fig S17

(A) Elevated TBET in the 10-HOME treated naïve $CD4^+$ T cells. Flow cytometry analyses of treated cells stained with anti-CD4 (FITC) and anti-TBET (PE). Representative flow plots. (i) vehicle-treated (ii) 10-HOME-treated (iii) histogram with isotype control for TBET (iv) % of $CD4^+$ $TBET^+$. Data presented as mean ± SD, (n=8-9).

(B) Elevated CD36 in the 10-HOME treated naïve $CD4^+$ 183^+ T cells. Flow cytometry analyses of treated cells stained with anti-CD36 (APC) and anti-CD183 (PE). Representative flow plots. (i) vehicle-treated (ii) 10-HOME-treated (iii) histogram with isotype control for CD36 (iv) % of $CD4^+$ $CD36^+$. Data presented as mean ± SD, (n=7).

(C) Flow cytometry analyses of treated cells stained with anti-CD4 (FITC) and anti-CD194 (PE). Representative flow plots. (i) vehicle-treated (ii) 10-HOME-treated (iii) histogram with isotype control for CD194 (iv) % of $CD4^+$ $CD194^+$. Data presented as mean ± SD, (n=8-10).

(D) Flow cytometry analyses of treated cells stained with anti-CD4 (FITC) and anti-GATA3 (PE). Representative flow plots. (i) vehicle-treated (ii) 10-HOME-treated (iii) histogram with isotype control for GATA3 (iv) % of $CD4^+$ $GATA3^+$. Data presented as mean ± SD, (n=6-8).

(E) Flow cytometry analyses of treated cells stained with anti-CD4 (FITC) and anti-CD196 (PE). Representative flow plots. (i) vehicle-treated (ii) 10-HOME-treated (iii) histogram with isotype control for CD196 (iv) % of $CD4^+$ $CD196^+$. Data presented as mean ± SD, (n=9).

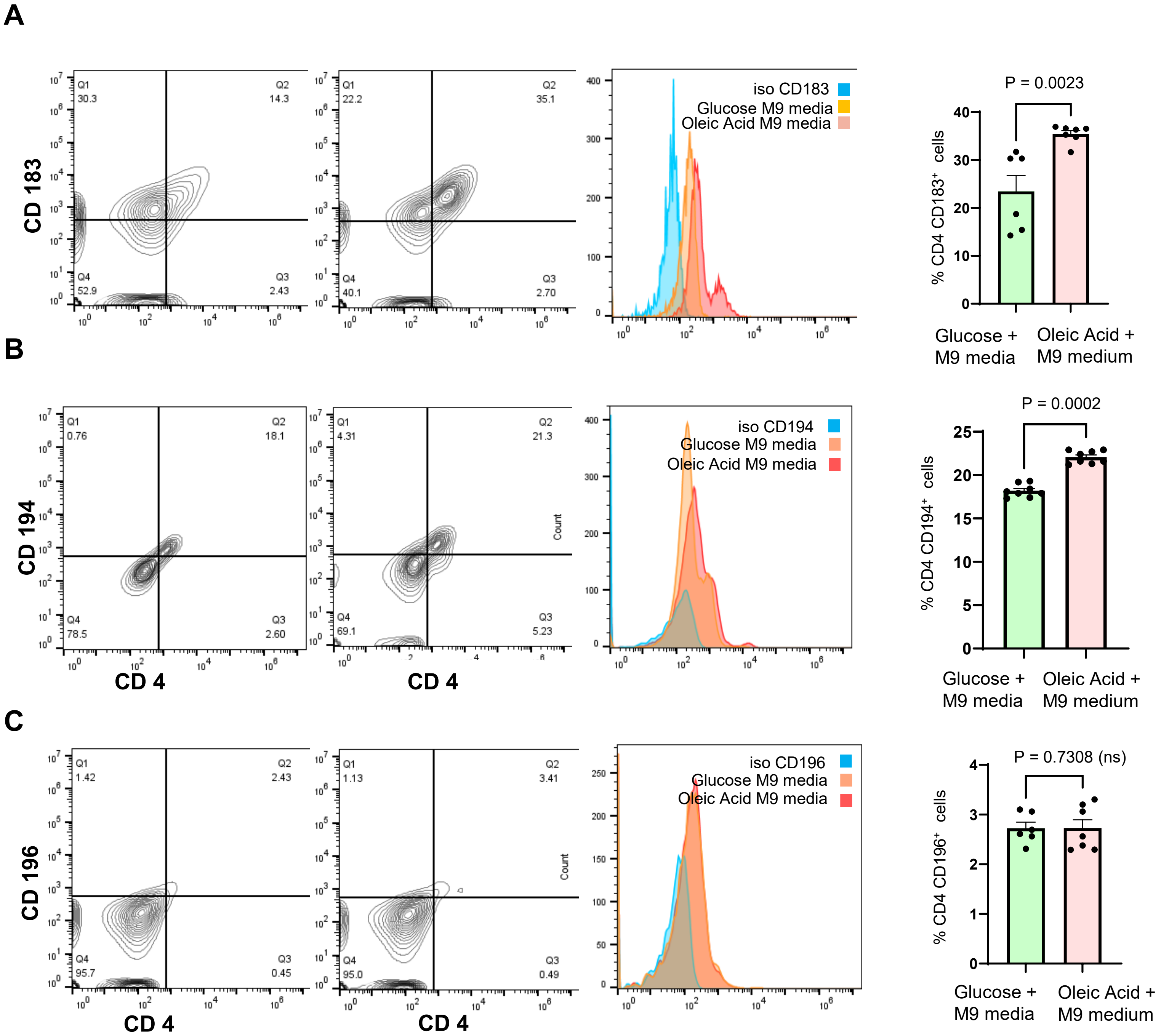

Supplementary Figure S18

(A) Elevated CD183 in the bacterial supernatant treated naïve $CD4^+$ T cells. Flow cytometry analyses of treated cells stained with anti-CD4 (FITC) and anti-CD183 (PE). Representative flow plots. (i) vehicle-treated (ii) 10-HOME-treated (iii) histogram with isotype control for CD183 (iv) % of $CD4^+$ $CD183^+$. Data presented as mean ± SD, (n=6-7).

(B) Flow cytometry analyses of treated cells stained with anti-CD4 (FITC) and anti-CD194 (PE). Representative flow plots. (i) vehicle-treated (ii) 10-HOME-treated (iii) histogram with isotype control for CD194 (iv) % of $CD4^+$ $CD194^+$. Data presented as mean ± SD, (n=8-10).

(C) Flow cytometry analyses of treated cells stained with anti-CD4 (FITC) and anti-CD196 (PE). Representative flow plots. (i) vehicle-treated (ii) 10-HOME-treated (iii) histogram with isotype control for CD196 (iv) % of $CD4^+$ $CD196^+$. Data presented as mean ± SD, (n=9).

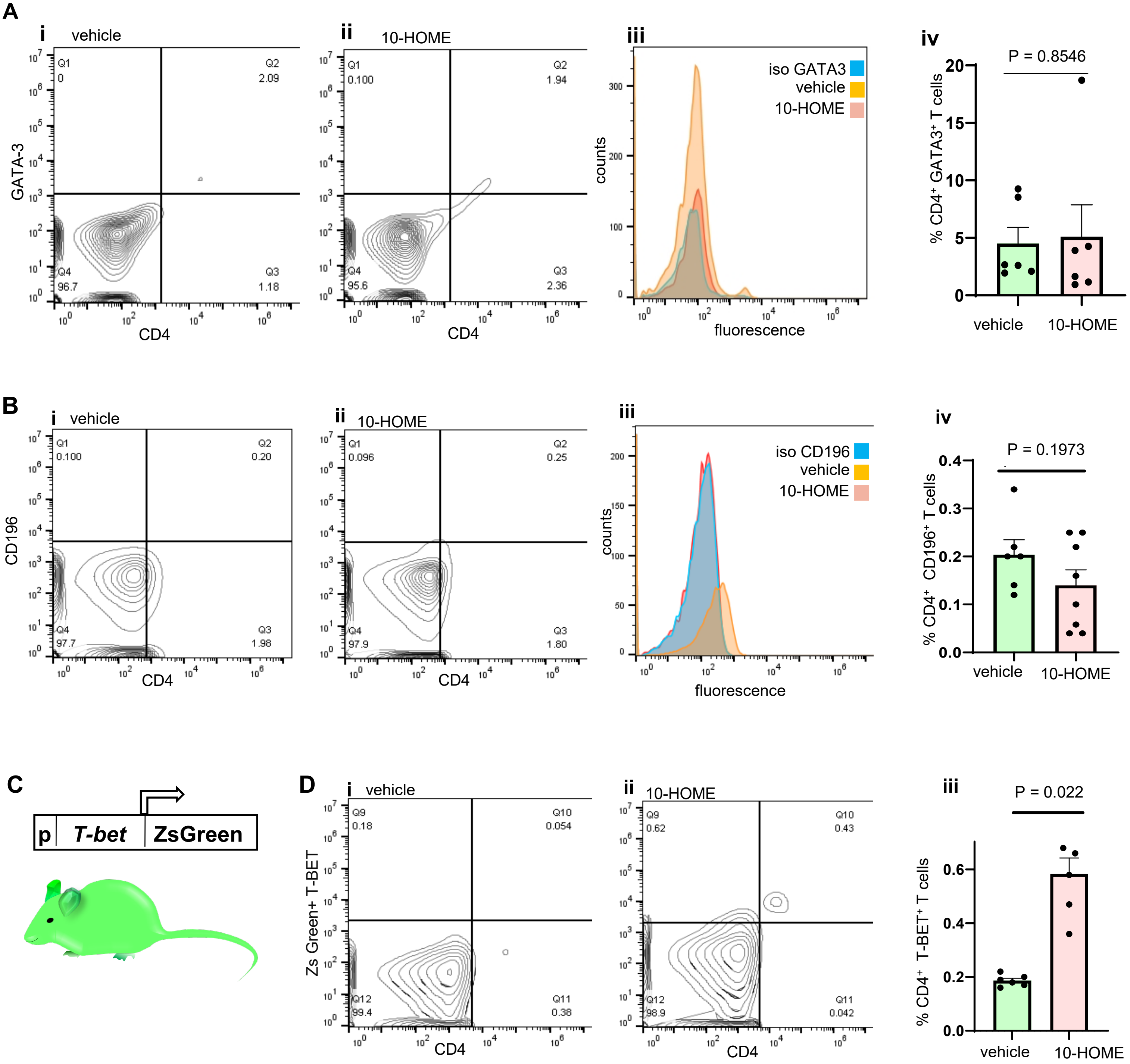

Supplementary Figure S19

Supplementary Fig S19

(**A**) Unaltered CD4^{+} GATA-3 subtype in the blood of mice injected with 10-HOME. Flow cytometry analyses of blood of mice stained with anti-CD4 (FITC) and anti-GATA-3 (PE). Representative flow plots. (i) Vehicle treated (ii) 10-HOME treated (iii) histograms of vehicle-treated and 10-HOME-treated animals with isotype control for GATA-3 (iv) % of CD4^{+} GATA-3^{+} T cells. Data presented as mean ± SEM, vehicle (n=6) and 10-HOME (n=6 mice).

(B) Unaltered CD4^{+} Th9/Th22 subtype in the blood of mice injected with 10-HOME. Flow cytometry analyses of blood of mice stained with anti-CD4 (FITC) and anti-CD196 (PE). Representative flow plots. (i) Vehicle-treated (ii) 10-HOME-treated (iii) histograms of vehicle-treated and 10-HOME-treated animals with isotype control for CD196 (iv) % of CD4^{+} CD196^{+} T cells. Data presented as mean ± SEM, vehicle (n=6) and 10-HOME (n=8) mice.

(C) Illustration of *Tbet*- ZsGreen reporter mouse to study Th1 cell activation *in vivo*.

(D) Elevated CD4^{+} TBET^{+} subtype in the blood of mice injected with 10-HOME. Flow cytometry analyses of blood of mice stained with anti-CD4 (APC) and T-BET (Zs-Green). Representative flow plots. (i) Vehicle-treated (ii) 10-HOME-treated (iii) % of CD4^{+} Zs-Green^{+} T cells. Data presented as mean ± SEM, vehicle (n=6) and 10-HOME (n=5) mice.

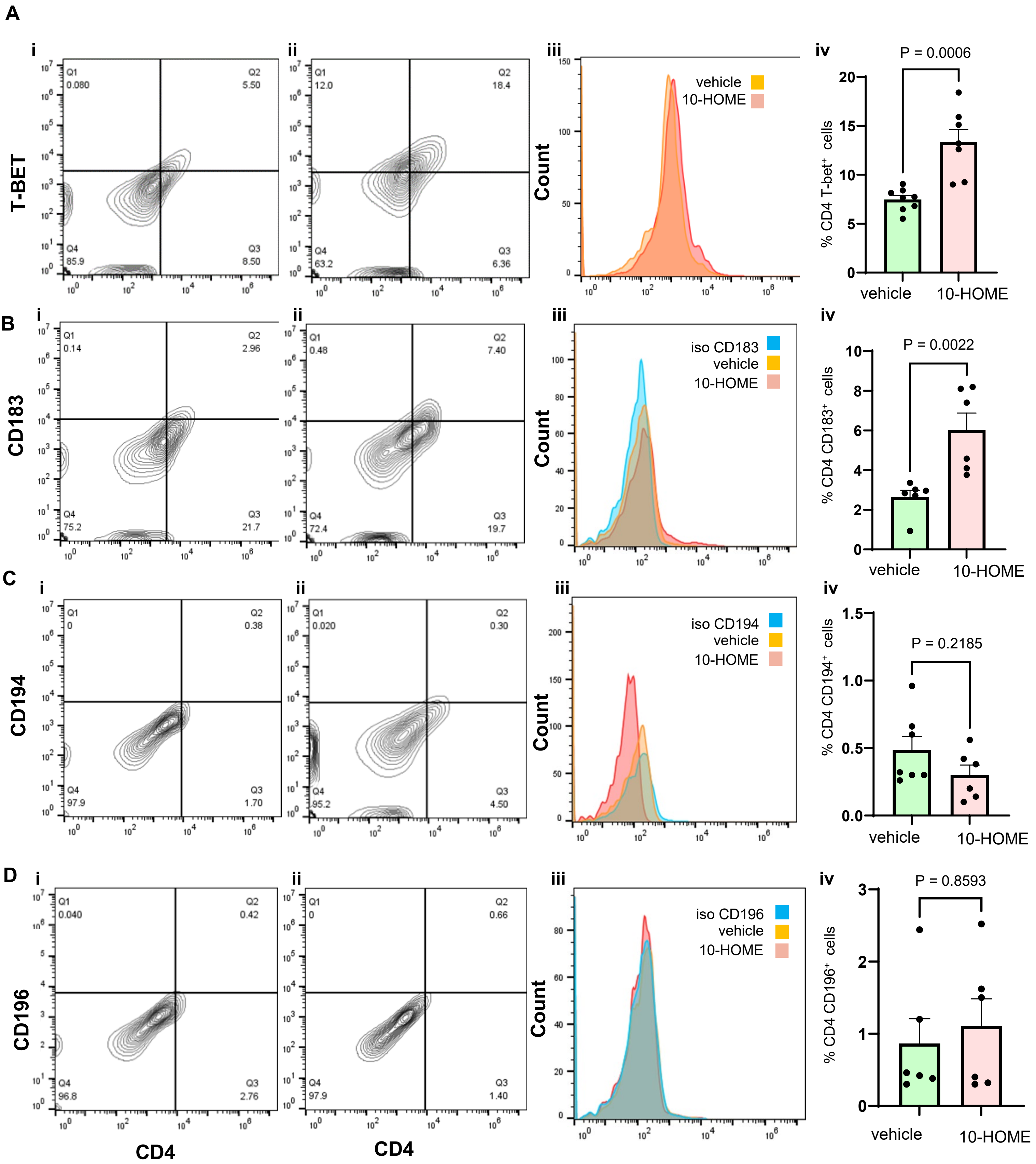

Supplementary Figure S20

Supplementary Figure S20

(A) Elevated $CD4^+$ $TBET^+$ subtype in the blood of mice injected with 0.5 mg/mL 10-HOME. Flow cytometry analyses of blood of mice stained with anti-CD4 (APC) and T-BET (Zs-Green). Representative flow plots. (i) Vehicle-treated (ii) 10-HOME-treated (iii) histograms of vehicle-treated and 10-HOME-treated animals (iv) % of $CD4^+$ $Zs\text{-}Green^+$ T cells. Data presented as mean ± SEM, vehicle (n=6) and 10-HOME (n=5) mice.

(B) Elevated $CD4^+$ 183^+ subtype in the blood of mice injected with 0.5 mg/mL 10-HOME. Flow cytometry analyses of blood of mice stained with anti-CD4 (FITC) and 183 (PE). Representative flow plots. (i) Vehicle-treated (ii) 10-HOME-treated (iii) histograms of vehicle-treated and 10-HOME-treated animals with isotype control for CD183 (iv) % of $CD4^+$ 183^+ T cells. Data presented as mean ± SEM, vehicle (n=6) and 10-HOME (n=6) mice.

(C) Elevated $CD4^+$ 194^+ subtype in the blood of mice injected with 0.5 mg/mL 10-HOME. Flow cytometry analyses of blood of mice stained with anti-CD4 (FITC) and 194 (PE). Representative flow plots. (i) Vehicle-treated (ii) 10-HOME-treated (iii) histograms of vehicle-treated and 10-HOME-treated animals with isotype control for CD194 (iv) % of $CD4^+$ 194^+ T cells. Data presented as mean ± SEM, vehicle (n=6) and 10-HOME (n=6) mice.

(D) Elevated $CD4^+$ 196^+ subtype in the blood of mice injected with 0.5 mg/mL 10-HOME. Flow cytometry analyses of blood of mice stained with anti-CD4 (FITC) and 196 (PE). Representative flow plots. (i) Vehicle-treated (ii) 10-HOME-treated (iii) histograms of vehicle-treated and 10-HOME-treated animals with isotype control for CD196 (iv) % of $CD4^+$ 196^+ T cells. Data presented as mean ± SEM, vehicle (n=6) and 10-HOME (n=6) mice.

A

Symbol	Entrez Gene Name	Expr Log Ratio	Expected
NRAS	NRAS proto-oncogene, GTPase	-0.238	Up
PIK3C3	phosphatidylinositol 3-kinase catalytic subunit type 3	0.179	Up
PIK3C2A	phosphatidylinositol-4-phosphate 3-kinase catalytic subunit type 2 alpha	0.241	Up
PIK3CA	phosphatidylinositol-4,5-bisphosphate 3-kinase catalytic subunit alpha	0.294	Up
PIK3R2	phosphoinositide-3-kinase regulatory subunit 2	-0.263	Up
PIK3R4	phosphoinositide-3-kinase regulatory subunit 4	0.105	Up
PIM1	Pim-1 proto-oncogene, serinethreonine kinase	-0.817	
RAP2B	RAP2B, member of RAS oncogene family	-0.325	Up
RASD2	RASD family member 2	-0.806	

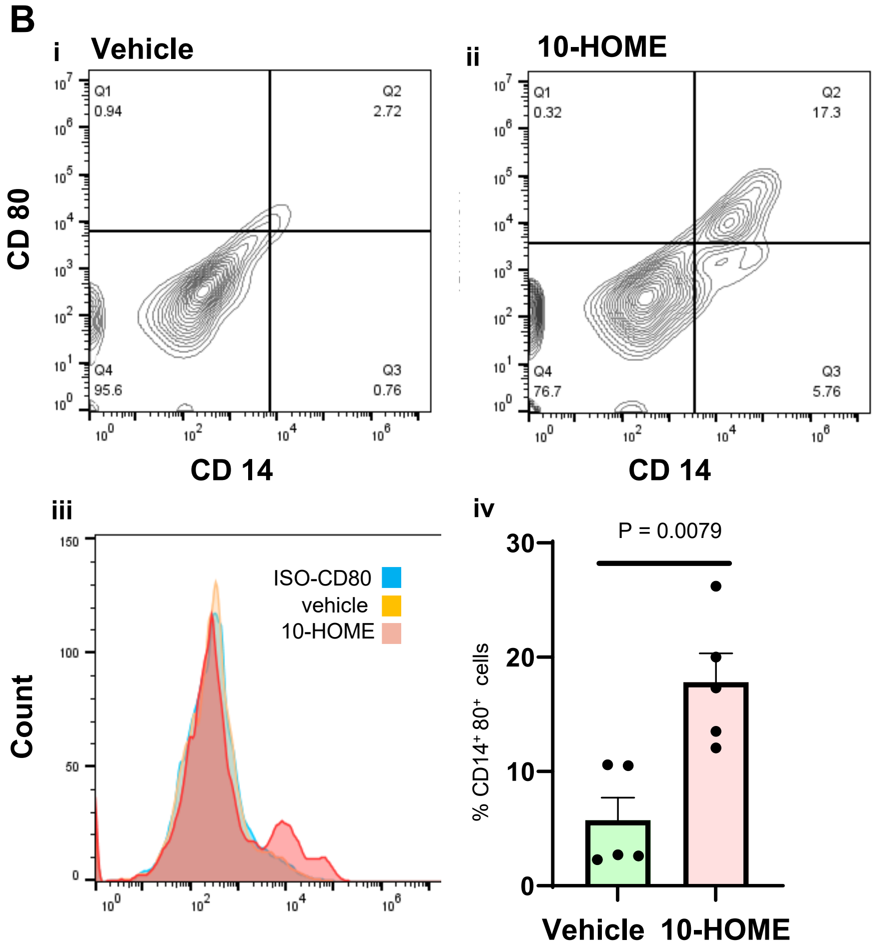

Supplementary Figure S21

(**A**) List of the de-regulated gene signature in human M1 cell population between BII, non-BII from RNA-Seq results.

(B) Increased polarization to M1 phenotype (CD80-human M1 marker) in the trans-well co-culture of human PBMC derived M0 macrophages incubated with 10-HOME treated naïve $CD4^{+}$ T cells. Flow cytometry analyses of treated cells stained with anti-CD80 (FITC) and anti-CD14 (PE). Representative flow plots. (i) vehicle-treated (ii) 10-HOME-treated (iii) histogram with isotype control for CD80 (iv) % of $CD14^{+}$ $CD80^{+}$. Data presented as mean ± SD, (n=5).

Supplementary Figure S21

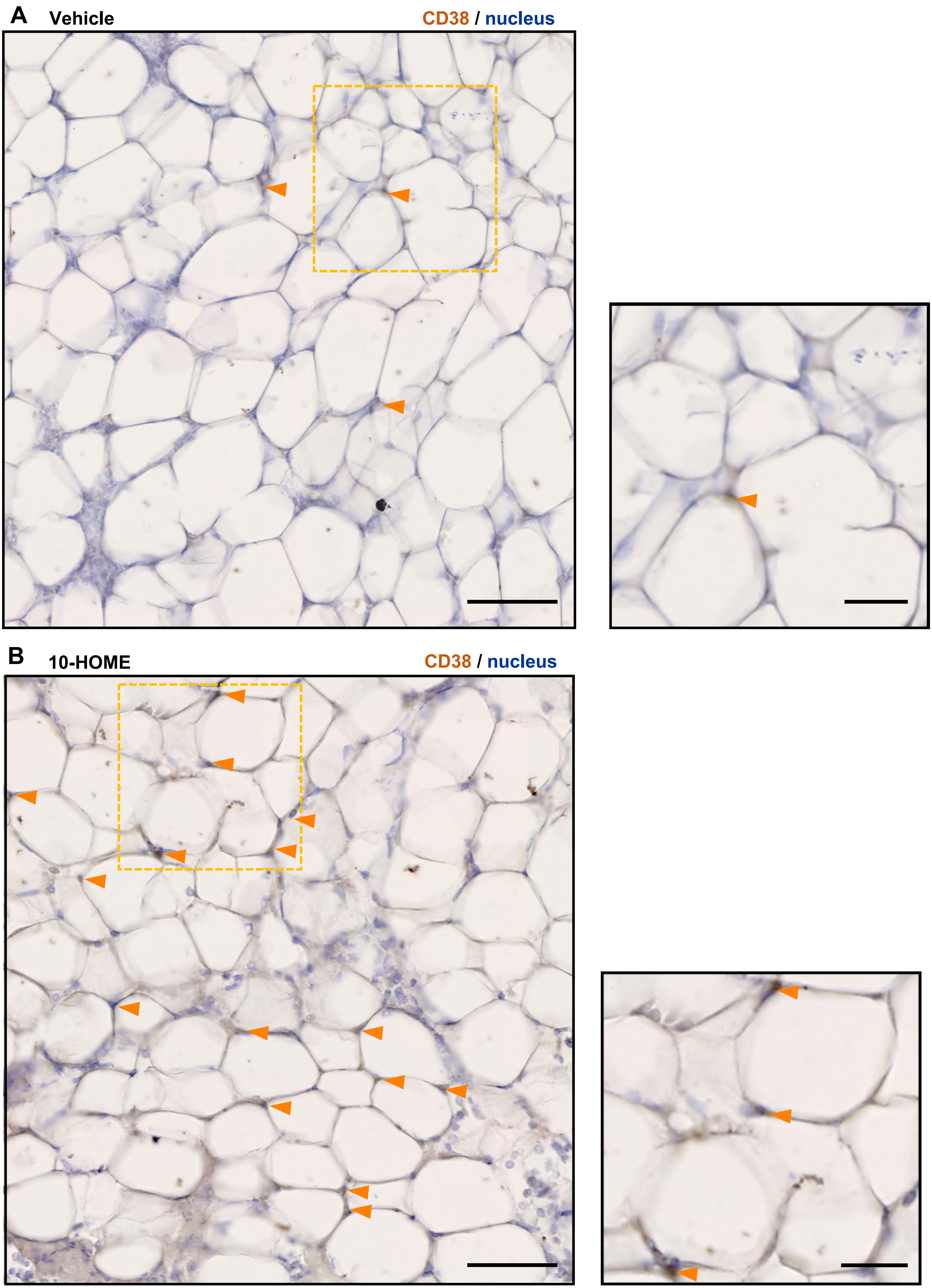

Supplementary Figure S22

Supplementary Figure S22

(**A-B**) Enlarged image exhibited in Figure 7B. Increased expression of CD38 (murine M1 macrophage marker) in 10-HOME-treated mice compared to vehicle. Murine mammary fat pads stained with anti-CD38 antibody. Staining of CD38 (DAB, brown), nucleus (hematoxylin, blue). Cells co-expressing brown (DAB) and blue (nucleus) were considered (shown in arrows). Scale bar (enlarged figure) = 100μm, scale bar (inset) = 20μm.

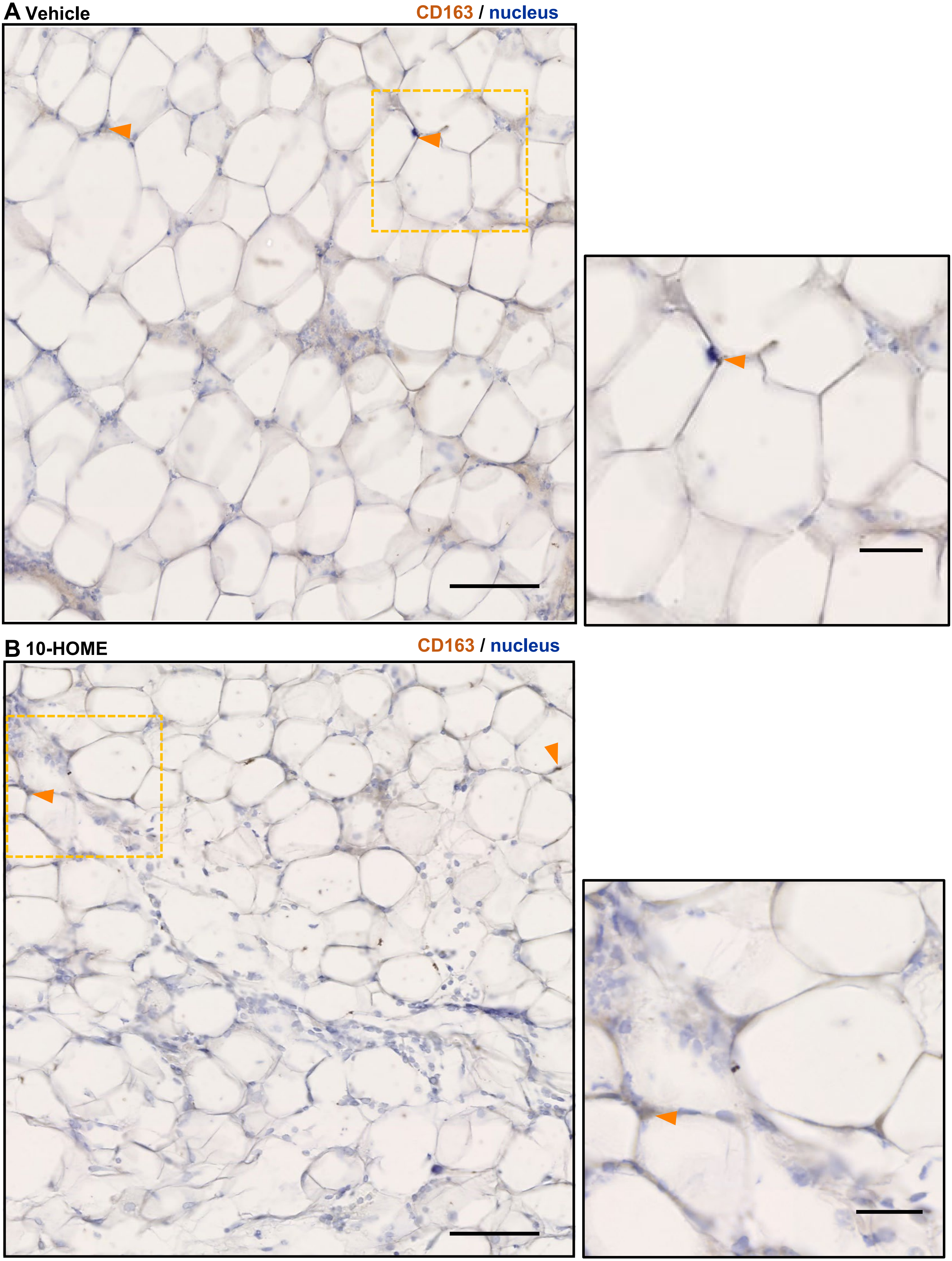

Supplementary Figure S23

Supplementary Figure S23

(**A-B**) Enlarged image exhibited in Figure 7C. No significant difference of CD163 (M2 macrophage marker) in 10-HOME-treated mice compared to vehicle. Staining of CD163 (DAB, brown), nucleus (hematoxylin, blue). Cells co-expressing brown and blue colors were considered (shown in arrows). Scale bar (enlarged figure) = 100μm, scale bar (inset) = 20μm.

Supplementary Tables

Supplementary Tables

Supplementary Table 1

Breast Implant Illness Questionnaire
Approved through IRB # 2003674175
Title: Molecular Mechanisms Associated with Breast Implant Complications

Name:
____
Date: ______________________

Date of Birth: ____/_______/_______ **Height:** _________ **Weight:**__________ R**ace/ Ethnicity:** ____________

Reason Implants Placed: Augmentation Reconstruction **If reconstruction, were you diagnosed with breast cancer?** Y / N

Date Implants Placed: ____/_______/_______ **Original Surgeon:** __________________

Implant Information

Manufacturer: ________________________________ **Type:** Saline/ Silicone Gel Implant or Expander

Texture: Smooth Textured **Infection**: Clinically infected / Clean

Did Individual have Mastitis: Yes / No

Placement: Above Muscle Below Muscle

Incision Placement: Transaxillary (underarm) Inframammary fold (breast crease) Periareolar (nipple) Other: _____________

Implant Removed as: Routine procedure / Due to self-reported complication of breast implant illness

Is individual diabetic? Yes / No **Individual received antibiotic irrigation:** Yes / No

Have you had any other breast implant surgeries not listed above (e.g. breast lift, revision augmentation, biopsy):
____
____

When did your symptoms begin?

____

____

What were your initial symptoms?____

____

When/how did you become aware of Breast Implant Illness (BII)?

____

<u>For office use only/Physician Notes:</u>

____

____

____

____

____

____

**Local Chest Area**					
Do you experience pain or a burning sensation around the implant and/or the upper or outer chest?	Not at all	A little bit	Some what	Quite a bit	Very much
Do you experience pain and swelling in the armpit areas?	Not at all	A little bit	Some what	Quite a bit	Very much
Do you experience discomfort from tissue tightness, implant weight or pressure?	Not at all	A little bit	Some what	Quite a bit	Very much
**General**					
I feel fatigued.	Not at all	A little bit	Some what	Quite a bit	Very Much
I have brain fog, such as difficulty concentrating or memory loss.	Not at all	A little bit	Some what	Quite a bit	Very much
I have unexplained weight gain or loss *(circle which)*? How much?	Not at all	A little bit	Some what	Quite a bit	Very much
I have difficulty losing or gaining weight?	Not at all	A little bit	Some what	Quite a bit	Very Much
I feel inflamed.	Not at all	A little bit	Some what	Quite a bit	Very Much
I suffer from poor sleep.	Not at all	A little bit	Some what	Quite a bit	Very Much
I have foul body odor.	Not at all	A little bit	Some what	Quite a bit	Very Much
I feel much older than my true age.	Not at all	A little bit	Some what	Quite a bit	Very Much
**Immune System**					
I have frequent sinus infection.	Never	Rarely	Somet imes	Often	Always
I have frequent urinary tract infections.	Never	Rarely	Somet imes	Often	Always
I have frequent yeast infections.	Never	Rarely	Somet imes	Often	Always
I have frequent viral infections.	Never	Rarely	Somet imes	Often	Always
I have swollen or tender lymph nodes (armpit, neck).	Never	Rarely	Somet imes	Often	Always
I have unexplained frequent fevers.	Never	Rarely	Somet imes	Often	Always
I have chills.	Never	Rarely	Somet imes	Often	Always
I have night sweats.	Never	Rarely	Somet imes	Often	Always
**Psychological**					
I feel depressed.	Never	Rarely	Somet imes	Often	Always

I feel anxious.	Never	Rarely	Sometimes	Often	Always
I feel hopeless.	Never	Rarely	Sometimes	Often	Always
I have panic attacks.	Never	Rarely	Sometimes	Often	Always
**Musculoskeletal**					
I have joint pain and /or swelling?<br><br>If true, circle all that apply: Neck, shoulders, elbows, hands, back, hips, knees, feet	Never	Rarely	Sometimes	Often	Always
I have muscle pain and weakness.	Never	Rarely	Sometimes	Often	Always
I have muscle twitching.	Never	Rarely	Sometimes	Often	Always
I have slow muscle recovery after exercise.	Never	Rarely	Sometimes	Often	Always
**Pain Intensity**					
How would you rate your pain on average? (0 = no pain, 10 = worst pain imaginable)<br><br>1 2 3 4 5 6 7 8 9 10					
**Skin**					
I have dry skin and hair.	Not at all	A little bit	Some what	Quite a bit	Very much
I have hair loss.	Not at all	A little bit	Some what	Quite a bit	Very much
I have skin rashes. Where?	Not at all	A little bit	Some what	Quite a bit	Very much
I have acne or acne-like eruptions.	Not at all	A little bit	Some what	Quite a bit	Very much
**Eyes**					
I have dry eye.	Not at all	A little bit	Some what	Quite a bit	Very much
I have vision changes or visual distortions.	Not at all	A little bit	Some what	Quite a bit	Very much
I have puffy eyes.	Not at all	A little bit	Some what	Quite a bit	Very much
I am sensitive to light.	Not at all	A little bit	Some what	Quite a bit	Very much

Respiratory					
I have a cough.	Not at all	A little bit	Some what	Quite a bit	Very much
I have chest congestion.	Not at all	A little bit	Some what	Quite a bit	Very much
I have shortness of breath.	Not at all	A little bit	Some what	Quite a bit	Very much
I have nasal discharge.	Not at all	A little bit	Some what	Quite a bit	Very much
**Heart**					
I feel palpitations.	Never	Rarely	Somet imes	Often	Always
I have chest pain.	Never	Rarely	Somet imes	Often	Always
**Gastrointestinal**					
I have food intolerance or allergies.	Never	Rarely	Somet imes	Often	Always
I have constipation.	Never	Rarely	Somet imes	Often	Always
I have diarrhea.	Never	Rarely	Somet imes	Often	Always
I have bloating.	Never	Rarely	Somet imes	Often	Always
I have abdominal pain.	Never	Rarely	Somet imes	Often	Always
I have reflux or gastritis.	Never	Rarely	Somet imes	Often	Always
I have dry mouth.	Never	Rarely	Somet imes	Often	Always
I have difficulty swallowing, a choking feeling, or a lump in my throat.	Never	Rarely	Somet imes	Often	Always
**Endocrine/Hormonal**					
I have temperature intolerance.	Never	Rarely	Somet imes	Often	Always
I have low libido.	Never	Rarely	Somet imes	Often	Always
I have heavy menstrual bleeding.	Never	Rarely	Somet imes	Often	Always
I have abnormal menstrual cycles.	Never	Rarely	Somet imes	Often	Always
I have symptoms of adrenal imbalance (slow healing, easy bruising).	Never	Rarely	Somet imes	Often	Always

Neurological					
I have metallic tastes.	Never	Rarely	Sometimes	Often	Always
I have dizziness.	Never	Rarely	Sometimes	Often	Always
I have ringing in my ears.	Never	Rarely	Sometimes	Often	Always
I experience numbness and tingling (e.g. arms, hands, fingers, legs, feet)	Never	Rarely	Sometimes	Often	Always
I have headaches (tension or typical).	Never	Rarely	Sometimes	Often	Always
I have migraines.	Never	Rarely	Sometimes	Often	Always
I have unusual facial or eye movements.	Never	Rarely	Sometimes	Often	Always

Do you feel your symptoms fall into one of these categories? If so, please check all that apply.

☐Allergic ☐Autoimmune/Immune Disruption ☐Hormone Disruption ☐Neurologic ☐Mechanical (physical weight or tension from the implant) ☐Psychological (anxiety, regret, dissatisfaction)

Do you have any of the following actual diagnoses (*check all that apply)*:

Endocrine: ☐Hypothyroid ☐Hyperthyroid ☐Hashimoto's ☐Grave's Disease ☐Infertility ☐Diabetes

Pulmonary: ☐Asthma

Autoimmune: ☐Rheumatoid arthritis ☐Lupus ☐Scleroderma ☐Dermatomyositis ☐Sjogren's Syndrome ☐Multiple sclerosis ☐Nonspecific connective tissue disease ☐Sarcoidosis ☐Raynaud's ☐Interstitial cystitis

Immune: ☐Allergies ☐Immune deficiency ☐Reactivation of viruses such as EBV or Varicella (shingles)

Musculoskeletal: ☐Degenerative arthritis ☐Fibromyalgia

Gastrointestinal: ☐Irritable bowel syndrome ☐Ulcerative colitis ☐Crohn's colitis ☐SIBO

Psychological: ☐Depression ☐Anxiety

Did you ever have mastitis? Yes No

Have you had a cholecystectomy (gallbladder removal)? Yes No *If yes, before or after implants?* Before After

Are you taking diabetic medications? Medication name(s):

____

____

____

Do you have a family history of autoimmune or connective tissue diseases? Yes No
If yes, please explain:

____

____

____

____

Have you had any testing related to the symptoms you are experiencing? Yes No
***If yes, please explain*:**

____

____

____

____

Were any of the test results reported as abnormal? Yes No
***If yes, please explain*:**

____

____

____

____

Name of physicians seen regarding symptoms:

Primary Care:

____

____

OB/GYN:

____

____

Functional Medicine:

____

____

Rheumatologist:

____

____

Neurologist:

____

____

Infectious Disease:

____

____

Other:

____

____

Any other history you would like to share: ______________________

Supplementary Table 2

S.No	**Group**	**Size N**	**Median age** (in years)	**Mean duration of implant placed** (in years)	**Type of implant (smooth or textured)**	**Race/Ethnicity**
1	BII	86	48.2	12.8	Smooth- 67<br>Textured- 19	Caucasian -84<br>African American-1<br>Asian -1
2	non-BII	55	53.3	13.2	Smooth- 35<br>Textured- 20	Caucasian -53<br>African American -1<br>Asian- 1
3	normal	37	42.1	not applicable	not applicable	Caucasian -33<br>African American-4

Supplementary Table 3

S.No	Sample group	Species identified NGS	Species identified pathology
1	BII	*Acinetobacter sps.*	No growth
2	BII	*Pseudomonas, Cutibacterium acnes*	No growth
3	BII	*Staphylococcus epidermidis,*	No growth
4	BII	*Acinetobacter sps. Pseudomonas sps*	No growth
5	BII	*Acinetobacter sps.*	No growth
6	BII	*Pseudomonas sps*	No growth
7	BII	*Acinetobacter sps. Cutibacterium acnes*	*Cutibacterium species*

8	BII	*Cutibacterium acnes, Chryseobacterium indologenes*	*Cutibacterium species*
9	non BII	*Cutibacterium acnes*	No growth
10	non BII	*Acinetobacter lwoffii*	No growth
11	BII	*Acinetobacter sps. Pseudomonas sps*	No growth
12	BII	*Staphylococcus epidermidis, Cutibacterium acnes, Acinetobacter sps.*	no growth
13	BII	*Staphylococcus aureus*	coagulase negative *Staphylococcus*
14	non BII	*Cutibacterium granulosum, Corynebacterium tuberculostearicum, Cutibacterium avidum, Staphylococcus hominis, Staphylococcus epidermidis, Cutibacterium acnes*	*Serratia marcescens and Corynebacterium species*

Supplementary Table 4

S.No	**Gene Name**	**Forward Primer**	**Reverse Primer**
1	hTBET	CTC ACA AAC AAC AAG GGG GC	TCA CGG CAA TGA ACT GGG TT
2	hCD36	ACT GAG GAC TGC AGT GTA GGA	AGT GGT TTC TAC AAG CTC TGG TT
3	hGAPDH	TGA CGC TGG GGC TGG CAT TG	GCT CTT GCT GGG GCT GGT GG
4	mTBGR	AAG GGC GAC GTG AGC ATG T	CAC GGA CTT GGC CTT GTA CAC

Footnote h- human, m-mouse

Key Resources Table

REAGENT or RESOURCE	SOURCE	IDENTIFIER
**Antibodies**		
Anti-T-bet / Tbx21 antibody [4B10] (ab91109)	Abcam	Cat# ab91109
Anti-GATA3 antibody (ab106625)	Abcam	Cat# ab106625
CD4 Antibody (GK1.5)	Novus Biologicals	Cat# NBP2-25191
CD4 Antibody (polyclonal)	Novus Biologicals	Cat# NBP1-19371
CD 36 Antibody (MF3)	Abcam	Cat # ab80080
Anti-CD38 antibody [EPR21079] (ab216343)	Abcam	Cat# ab216343
Anti-CD163 antibody [EPR19518] (ab182422)	Abcam	Cat# ab182422
Receptor antibody (ab64693) MRC-1 CD206	Abcam	Cat# ab64693
T-bet/TBX21 Antibody (39D) (monoclonal)	Novus Biologicals	Cat# NBP1-43299
Goat Anti-Rabbit Invitrogen IgG H&L (Alexa Fluor® 488)	Thermo Fisher Scientific	Cat# A-11008
Goat Anti-Mouse IgG H&L (Alexa Fluor® 488)	Thermo Fisher Scientific	Cat# A-11001
Goat anti-Rabbit IgG (H+L) Cross-Adsorbed Secondary Antibody, Alexa Fluor 568	Thermo Fisher Scientific	Catalog # A-11011

Goat Anti-Rat IgG H&L (Alexa Fluor® 568)	Abcam	Catalog # ab175476
PE anti-mouse CD183 (CXCR3) Antibody - Clone CXCR3-173	Biolegend	Cat# 126506
PE Armenian Hamster IgG Isotype Ctrl Antibody - Clone HTK888	Biolegend	Cat# 400907
PE anti-mouse CD196 (CCR6) Antibody - Clone 29-2L17	Biolegend	Cat# 129804
APC anti-mouse CD194 (CCR4) Antibody -- Clone 2G12	Biolegend	Cat# 131211
FITC anti-mouse CD4 Antibody- Clone GK1.5	Biolegend	Cat# 100406
APC anti-mouse CD36 Antibody - Clone HM36	Biolegend	Cat# 102612
PE anti-human CD183 (CXCR3) Antibody- Clone G025H7	Biolegend	Cat# 353705
Alexa Fluor® 700 anti-human CD196 (CCR6) Antibody- Clone G034E3	Biolegend	Cat# 353433
APC Mouse IgG1, κ Isotype Ctrl Antibody	Biolegend	Cat# 400119
PE Mouse IgG1, κ Isotype Ctrl (FC) Antibody	Biolegend	Cat# 400113
FITC Rat IgG2b, κ Isotype Ctrl Antibody	Biolegend	Cat# 400605
Alexa Fluor® 700 Mouse IgG2b, κ Isotype Ctrl Antibody	Biolegend	Cat# 400334
PE anti-human CD36 Antibody - Clone 5-271	Biolegend	Cat# 336205

PE Mouse IgG2a, κ Isotype Ctrl (FC) Antibody	Biolegend	Cat# 400213
Alexa Fluor® 700 Mouse IgG1, κ Isotype Ctrl Antibody	Biolegend	Cat# 400143
PE Rat IgG2a, κ Isotype Ctrl Antibody	Biolegend	Cat# 400507
PE Rat IgG2b, κ Isotype Ctrl Antibody	Biolegend	Cat# 400607
Mouse IgG2a K Isotype Control FITC	ebioscience	Cat# 11-4724-81
FITC Mouse IgG1, κ Isotype Ctrl (FC) Antibody	Biolegend	Cat# 400109
FITC anti-human CD4 Antibody -- Clone- OKT4	Biolegend	Cat# 317408
FITC Mouse IgG2b, κ Isotype Ctrl Antibody	Biolegend	Cat# 400309
APC Armenian Hamster IgG Isotype Ctrl Antibody	Biolegend	Cat# 400911
PE anti-T-bet Antibody -- Clone 4B10 (also reacts with mouse)	Biolegend	Cat# 644809
PE anti-human CD36 Antibody - Clone 5-271	Biolegend	Cat# 336206
PE anti-human CD194 (CCR4) Antibody - Clone L291H4	Biolegend	Cat# 359412

PE Mouse IgG1, κ Isotype Ctrl (ICFC) Antibody - Clone MOPC-21	Biolegend	Cat# 400139
PE anti-human CD196 (CCR6) Antibody- Clone G034E3	Biolegend	Cat# 353410
PE Mouse IgG2b, κ Isotype Ctrl Antibody- Clone MPC-11	Biolegend	Cat# 400313
APC anti-human CD36 Antibody - Clone 5-271	Biolegend	Cat# 336208
PE anti-GATA3 Antibody- clone 16E10A23	Biolegend	Cat# 653804
APC anti-human CD194 (CCR4) Antibody - Clone L291H4	Biolegend	Cat# 359407
FITC anti-human CD80 Antibody - Clone W17149D	Biolegend	Cat# 375405
FITC anti-human CD86 Antibody - Clone BU63	Biolegend	Cat# 374203
APC anti-human CD206 (MMR) Antibody - Clone 15-2	Biolegend	Cat# 321109
APC anti-human CD163 Antibody - Clone GHI/61	Biolegend	Cat# 333609
FITC anti-human CD64 Antibody - Clone 10.1	Biolegend	Cat# 305005
PE anti-human CD14 Antibody - Clone M5E2	Biolegend	Cat# 301805
ABC Elite Kite Rabbit IgG	Vectastain	Cat# PK-6101
APC anti-mouse CD4 Antibody - Clone GK1.5	Biolegend	Cat# 100411
Chicken anti-Mouse IgG (H+L) Secondary Antibody	Invitrogen	Cat# A15975
Anti-Human CD14(M5E2)	Standard BioTools	Cat# 3151009B
Anti-Human CD25 (2A3)-149Sm	Standard BioTools	Cat# 3149010B
Anti-Human CD16 (3G8)-148Nd	Standard BioTools	Cat# 3148004B
Anti-Human CD19 (HIB19)-142Nd	Standard BioTools	Cat# 3142001B
Anti-Human CD161 (HP-3G10)-164Dy	Standard BioTools	Cat# 3164009B

Anti-Human CD36 (5-271)-155Gd	Standard BioTools	Cat# 3155012B
Anti-Human CD4 (RPA-T4)-145Nd	Standard BioTools	Cat#3145001B
Anti-Human CD45RA (HI100)-143Nd	Standard BioTools	Cat#3143006B
Anti-Human CD183/ CXCR3 (G025H7)-156Gd	Standard BioTools	Cat#3156004B
Anti-Human CD194/ CCR4 (L291H4)-158Gd	Standard BioTools	Cat#3158032A
Anti-Human CD196/ CCR6 (G034E3)-176Yb	Standard BioTools	Cat#3176022A
Anti-Human TCRgd (11F2)-152Sm	Standard BioTools	Cat#3152008B
Anti-Human CD49b (P1E6-C5)-161Dy	Standard BioTools	Cat#3161012B
Anti-Human/Mouse Tbet (4B10)-160Gd	Standard BioTools	Cat#3160010B
Anti-Human/Mouse Gata3 (TWAJ)-167Er	Standard BioTools	Cat#3167007A
Anti-Human CD223/ LAG-3 (11C3C65)-165Ho	Standard BioTools	Cat#3165037B
Anti-Human CD278/ ICOS (C398.4A) -169Tm	Standard BioTools	Cat#3169030B
Anti-Human CD152/ CTLA-4 (14D3)-170Er	Standard BioTools	Cat#3170005B
Anti-Human CD3 (UCHT1)-141Pr	Standard BioTools	Cat#3141019B

Anti-Human CD279/ PD-1 (EH12.2H7)- 175Lu	Standard BioTools	Cat#3175008B
**Dyes**		
DAPI	Sigma Aldrich	Cat# D9542
Wheat Germ Agglutinin, Alexa Fluor™ 488 Conjugate	Invitrogen	Cat# W11261
3,3' Diaminobenzidine (DAB)	Sigma Aldrich	Cat# D5905
Gill III hematoxylin	Leica Biosystems	Cat#3801542
**Bacterial Strains**		
Staphylococcus Epidermidis	ATCC	Cat# 35984
Cutibacterium acnes	ATCC	Cat# 6919
Pseudomonas fluorescence	ATCC	Cat# 13525
Acinetobacter sps	ATCC	Cat# 14290
Sphingomonas sps	ATCC	Cat# BAA-1889
Enterobacter cloacae	ATCC	Cat# 13047
Cornyebacterium tuberculostearicum	ATCC	Cat#35692
**Chemicals, Lipids, and Recombinant Proteins**		
Recombinant IL-2	Provided kindly by Dr. Alan L Epstein Professor of Pathology. Keck School of Medicine, University of Southern California	

Normal Goat Serum Blocking Solution	Vector Labs, Inc	Cat# S-1000
eBioscience™ IC Fixation Buffer	Thermo Fisher Scientific	Cat# 00-8222-49
VECTASHIELD® Hardset™ Antifade Mounting Medium	Vector Labs, Inc	Cat# H-1500-10
Gibco™ DPBS	Thermo Fisher Scientific	Cat# 14190250
Gibco™ DMEM, low glucose, pyruvate	Thermo Fisher Scientific	Cat# 11885092
Gibco™ Fetal Bovine Serum	Thermo Fisher Scientific	Cat# 16000044
Gibco™ Penicillin-Streptomycin (10,000 U/mL)	Thermo Fisher Scientific	Cat# 15140122
Gibco™ Trypsin-EDTA (0.5%), no phenol red	Thermo Fisher Scientific	Cat# 15400054
RPMI 1640	Thermo Fisher Scientific	Cat# 21875034
True-Nuclear™ Transcription Factor Buffer Set	BioLegend	Cat# 424401
M9 Minimal Salts	Thermo Fisher Scientific	Cat# A1374401
Hydrogen Peroxide, 30% (Certified ACS)	Thermo Scientific	Cat#H325-30GAL
RBC lysis buffer	BioLegend	Cat# 420302
**Critical Commercial Assays**		
Human IFN-gamma DuoSet ELISA	R&D systems	Cat# DY285B-05
Human IL-10 DuoSet ELISA	R&D systems	Cat# DY217B-05

Quant-iT™ Protein Assay Kit	Molecular probes	Cat# Q33210
SYBR™ Green PCR Master Mix	Fisher Scientific	Cat# 43-091-55
Invitrogen™ SuperScript™ III First-Strand Synthesis System	Thermo Fisher Scientific	Cat# 18080051
SuperScript™ VILO™ cDNA Synthesis Kit	Thermo Fisher Scientific	Cat# 11754050
Applied Biosystems™ TaqMan™ Universal PCR Master Mix, No AmpErase™ UNG	Thermo Fisher Scientific	Cat# 4324018
GenElute Gel Extraction Kit	Sigma	Cat# NA1111-1KT
Human Albumin ELISA Kit	Abcam	Cat# ab227933
Human Serum Albumin DuoSet ELISA	R&D systems	Cat#DY1455
**Experimental model: Mice**		
C57BL/6J	Jackson	Cat#000664
Tbet-ZsGreen	NIH/NIAID	Mice line # 8419
**Experimental model: Cell lines**		
TLBR1 cell line	Provided kindly by Dr. Dr. Alan L Epstein Professor of Pathology. Keck School of Medicine, University of Southern California.	
MAC2A, MAC2B	Provided kindly by Dr. Marshall Kadin, MD. Professor of Pathology	

	Department of Pathology, University of Virginia, Charlottesville, Virginia	
**Biological samples**		
Human Breast Implant Periprosthetic tissue	Indiana University Health, Indianapolis<br>Meridian Plastic Surgeons. Indianapolis	
**Oligonucleotides**		
See Table 4	IDT	
**Software and Algorithms**		
ImageJ version 1.8.0_112	NIH<br>https://imagej.nih.gov/ij/download.html	
Zen Blue 2.3		
Zen Black	Zeiss	
GraphPad Prism 8.4.2		
GraphPad software	https://www.graphpad.com/scientific-software/prism/	
FlowJo_v10.7.1	https://www.flowjo.com/solutions/flowjo/downloads	
Cytobank software	https://premium.cytobank.org/cytobank	

Supplementary Methods

Supplementary Methods

NGS sequencing for 16S rRNA. The peri-prosthetic breast tissue (breast parenchyma in contact with the capsule and implant) was used for 16S rRNA based NGS analyses. It is to be noted that breast soft tissue in contact with capsule and implant is the site of host-biofilm interaction. As the biofilms are located in pockets and not homogenously distributed. Hence, the periprosthetic tissue in association with capsule and implant was collected from multiple locations and homogenously grounded. Capsules as being comprised of collagen are hard and difficult to homogenize and hence were not used for 16S rRNA analyses. Microbial DNA in each sample were sequenced by MicrogenDx Inc using the Illumina MiSeq sequencer. Forward and reverse primers were used to detect and amplify the target sequence, for 16S gene in bacteria. The samples are differentiated from each other when run on the MiSeq sequencer by a "tag," a unique identifying sequence attached to the forward and reverse primers implemented when the targeted sequence is amplified using PCR. Following PCR, purification of the pooled DNA was done by removing small fragments using both Agencourt Ampure beads and Qiagen Minelute kit. The DNA was quantified and prepared for sequencing. Finally, the DNA library was run on the MiSeq sequencer. The sequencing reads were analyzed for quality and length during the data analysis. The data analysis pipeline consisted of two major stages, the denoising and chimera detection stage and the microbial diversity analysis stage. During the denoising and chimera detection stage, denoising was performed using various techniques to remove short sequences, singleton sequences, and noisy reads. With the low-quality reads removed, chimera detection was performed to aid in the removal of chimeric sequences. The high-quality sequencing reads of the variable region of 16S rRNA were compared to curated database of MicrogenDx. The database is comprised of 18500 unique bacteria.

Scanning Electron Microscope Imaging. The samples were collected in glutaraldehyde fixation buffer, dehydrated with graded ethanol, and treated with hexamethyldisilazane (HMDS, Ted Pella

Inc.) and left overnight for drying(1, 2). Before scanning, samples were mounted and coated with gold. Samples were imaged with FEI™ NOVA nanoSEM scanning electron microscope (FEI™, Hillsboro, OR) equipped with a field-emission gun electron source.

Wheat-germ agglutinin (WGA) staining. Paraffin embedded capsules surrounding the implant were sectioned on the slide. Wheat Germ Agglutinin, Alexa Fluor™ 488 Conjugate (Invitrogen) stock solution (1mg/ml) was diluted in PBS. The sections were stained with Wheat Germ Agglutinin (dilution 1:200) for 10 mins (3). The slides were then mounted and imaged on a Zeiss LSM 880 microscope equipped with the AIRYscan detector.

Synthesis, and Validation of 10-HOME. For the synthesis of 10-HOME, a convergent Horner-Wadsworth-Emmons approach was employed. Indiana University has filed a provisional patent application (Application # 63/107,626) on behalf of REM, IK and MS relating to the methods and synthesis of 10-HOME and its deuterated 17,17,18,18,18-d_5 analog to be used as analytical standards. The synthesized 10-HOME internal standard was purified by silica-gel flash column chromatography as the methyl ester and analyzed by thin-layer chromatography (TLC). Fractions containing 10-HOME methyl ester (yellow spots were detected through permanganate staining) were collected and validated by gas chromatography-mass spectrometry (GC-MS). The standard was derivatized with *N,O*-bis(trimethylsilyl)trifluoroacetamide – 2% chlorotrimethylsilane (1h, 65°C) prior to analysis. GC-MS parameters: Agilent 7890/5975C system using a VF-23ms column (30m, 250 μm film, 0. 25 mm diameter) and an oven program starting at 60°C, ramp 7°C/min to 150°C, hold at 150°C for 7 min, ramp 10°C/min to 220°C, hold 1 min, ramp 50°C/min to 250°C, and hold 2 min; He flow 1.9 mL min; retention time 22 min. The parent ion and MS fragmentation was consistent with methyl 10-trimethylsilyl-8-octadecenoate. The Me ester was additionally characterized by proton and 13C NMR spectroscopy. Purified 10-HOME methyl ester was then hydrolyzed to provide 10-HOME free acid. The synthetic 10-HOME free acid standard was validated using proton nuclear magnetic resonance (NMR) spectroscopy. Data was collected in

CDCl3 that had been deacidified by passage through a column of neutral alumina. Data was collected on a Bruker Avance II 500-MHz NMR spectrometer using a standard proton NMR pulse sequence. Proton-decoupled 13C NMR data further supported the identity and purity of the standard. All data was solvent referenced to d 7.26 (1H) and d 77.0 ppm (13C).

Immunohistochemistry and immunocytochemistry. Paraffin embedded breast tissue blocks were sectioned, deparaffinized and immunostained(2, 4, 5). Immunohistochemical staining of the sections were performed using standard procedures using the following primary antibodies: α-CD4 antibody (Abcam# ab133616; dilution 1:200), α-CD36 antibody (Abcam # ab80080, clone MF3, dilution: 1:200), α- T-bet antibody (Abcam # ab91109, clone 4B10, dilution: 1:200). CD38 antibody (Abcam # ab216343, dilution 1:200), CD163 antibody (Abcam # ab182422, dilution 1:200). For the IHC, the slides were incubated with horseradish peroxidase (HRP)–conjugated rabbit IgG for 1 hour at 37°C, followed by staining with 3, 3 -diaminobenzidine (DAB) and hematoxylin. Cells co-expressing brown (DAB) and blue (nucleus) colors were considered for analyses. In the adipose tissue, the adipose cells filled with lipid occupy the majority of tissue. Other cells are present in borders of adipose cells. To enable fluorescence detection, sections were incubated with appropriate Alexa Fluor® 488 (green, Molecular probes), or Alexa Fluor® 564 (red, Molecular probes) conjugated with secondary antibodies. The sections were counterstained with DAPI (Sigma). For immunocytochemistry, cells were fixed with IC fixation buffer (eBioscience), blocked with 10 percent normal goat serum (Vector Laboratories), incubated with primary and secondary antibodies and counterstained with DAPI. Mosaic images were collected using a Zeiss Axiovert 200 M, inverted fluorescence microscopy or confocal microscopy (LSM880). Image analysis was performed using Zen (Zeiss) software. Fluorescence Intensity was calculated by integrated density using the ZEN™ software provided by Zeiss®. Total fluorescence was accounted by subtracting out background signal,

which is useful for comparing the fluorescence intensity between cells or regions. Area, Integrated Density and Mean Grey Value were subsequently evaluated.

Lipid extraction and 10-HOME quantification using LCMS. LC-MS/MS targeted analysis from capsule and breast adipose tissue was performed. Samples were weighed and transferred to 2 ml vials with 1.4 mm ceramic beads and 1 ml of water with 0.1% formic acid. The standard solution was prepared by aliquoting 1µl of each stock solution into a new tube drying the original solvent and solubilizing in 1 ml of 100% ethanol to obtain a final concentration of 1 ng/ml each. Samples were homogenized using Precellys 24 tissue homogenizer (Bertin Technologies, Rockville, MD). The total volume of the homogenate was extracted with ethyl acetate in a 1:1 volume ratio. Samples were vortexed for 1 minute and centrifuged at 14,000 rpm for 10 minutes. The organic phase was collected and transferred to a new vial to be evaporated and stored at -80°C until analysis. The dried lipid extracts were reconstituted with 50 µl of methanol/water at 1:1 volume ratio and submitted for targeted quantification by liquid chromatography tandem MS (LC/MS/MS). The LC column used was an Acquity UPLC BEH C18 1.7µm particle size - 2.1x100 mm (Waters, Milford, MA). The binary pump flow rate was set at 0.3mL/min in an Agilent UPLC (G7120A) using water and 0.1% formic acid as mobile phase A and acetonitrile and 0.1% formic acid as mobile phase B. The LC column was pre-equilibrated with 80% A for 1 min. The binary pump was set in a linear gradient to 100% B in 8 min and held for 2.50 min. It was then returned to 80% A and re-equilibrated for 4 min. Ten µL of the reconstituted sample was delivered to the column through a multisampler (G7167B) into a QQQ6470A triple quadrupole mass spectrometer (Agilent Technologies, San Jose, CA) equipped with ESI Jet Stream ion source. In the mass spectrometer the capillary voltage was 3500 V on the negative ion mode, the gas temperature was 325ºC, gas flow was set at 8l/min, the sheath gas heater at 250ºC and the sheath gas flow at 7 l/min. The fragmentation voltage was 100V and the cell accelerator voltage was 4 V. The MRMs (parent-fragment) for the acquisition included were m/z 297.5->155.4 for 10-HOME and for the internal

standard it was m/z 302.4->155.4. Concentrations in pg/mg of tissue were obtained by normalizing by the dried weight of the sample homogenized and by the concentration of the deuterated internal standard. To quantify 10-HOME, calibration curves were done with 5 serial dilutions of the stock solution starting at 100 ng as the highest amount. Data processing was carried out by using Mass Hunter (B.06.00) using software Quant and Qual.

Flow cytometry analyses. The fluorescence and light-scattering properties (forward scatter and side scatter) of the cells were determined by using BD Accuri C6. Signals from cells labeled with conjugated fluorophores were detected. The following antibodies were used for different flow cytometry analysis. PE anti-human CD183 (CXCR3) (clone G025H7, Biolegend # 353705, 2 μg/ml), PE anti-human CD194 (CCR4) (clone L291H4, Biolegend # 359412, 0.5 μg/ml), PE anti-human CD196 (CCR6) (clone G034E3, Biolegend # 353410, 0.5 μg/ml), FITC anti-human CD4 (clone A161A1, Biolegend # 357406 , 0.5 μg/ml), APC anti-human CD194 (CCR4) (clone L291H4, Biolegend # 359407, 0.5 μg/ml), Alexa Fluor® 700 anti-human CD196 (CCR6) (clone G034E3, Biolegend # 353433, 0.5 μg/ml), PE anti-human CD36 (clone 5-271, Biolegend # 336205, 1 μg/ml), FITC anti-mouse CD4 (clone GK1.5, Biolegend # 100406, 0.5 μg/ml), APC anti-mouse CD36 (clone HM36, Biolegend # 102612, 0.5 μg/ml), PE anti-mouse CD183 CD36 (clone CXCR3-173, Biolegend # 126506, 0.5 μg/ml), PE anti-mouse CD196 (clone 29-2L17, Biolegend # 129804, 0.5 μg/ml), APC anti-mouse CD194 (clone 2G12, Biolegend # 131211, 0.5 μg/ml), PE anti-mouse FOXP3 (clone MF14, Biolegend # 126403, 0.5 μg/ml), PE anti-T-bet Antibody (clone 4B10, Biolegend # 644809, 0.5 μg/ml), FITC anti-human CD86 Antibody (Clone BU63, Biolegend #374203, 0.5 μg/ml), APC anti-human CD163 Antibody (Clone GHI/61, Biolegend#333609, 0.5 μg/ml), PE anti-human CD14 Antibody (Clone M5E2, Biolegend#301805). For intracellular markers, TBET and GATA3 permeabilization was performed through True-nuclear transcription factor buffer set (Biolegend # 424401). Auto compensation was performed using samples stained with single flurophores. For macrophage related flow cytometry, cells were treated with Fc

receptor blocking solution (Biolegend) to block the Fc receptor displayed on the surface of antigen presenting cells (like macrophages). Gates were set manually. BD Diva (BD Biosciences), and FlowJo softwares were used for analyses(5). Logarithmic scale was used to measure cell fluorescence. Appropriate IgG control fluorescence compensation was applied to avoid false positive signals.

Primary T-cell isolation. Naïve $CD4^+$ T cells were isolated from peripheral blood mononuclear cells (PBMC) of healthy individuals obtained from blood bank Versiti. Briefly, PBMCs were isolated by Ficoll-Paque PLUS density gradient sedimentation. Naïve $CD4^+$ T cells were then enriched using immunomagnetic, column-free, negative selection (Naïve CD4 T cell isolation kit, Miltenyi Biotec)(6). Unwanted cells (CD8, CD14, CD15, CD16, CD19, CD25, CD34, CD36, CD45RO, CD56, CD123, TCRγ/δ, HLA-DR, and CD235a (Glycophorin A)) were removed using antibody complexes recognizing non-naïve CD4 T cells and dextran-coated magnetic particles.

Primary $CD4^+$ T cell culture and 10-HOME treatment. Primary $CD4^+$ T cells were cultured under standard conditions at 37°C in a humidified incubator with 5% CO_2 in RPMI-1640 growth medium supplemented with 10% FBS, 100 IU/ml penicillin, 0.1 mg/ml streptomycin, 10 mmol/l L-glutamine supplemented with IL2 for 48h(7). Following that, $CD4^+$ T cells were treated with oxylipin 10-HOME (100 μM) or vehicle control for 48 h.

Primary $CD4^+$ T cell culture and *S. epidermidis* supernatant treatment. Primary CD4+ T cells were treated with 10 μL of 0.22 micron filtered supernatant obtained from *Staphylococcus epidermidis* (Winslow and Winslow) Evans (ATCC 35984) cultured in M9 medium containing Oleic acid at the desired concentration of 1%(v/v) or vehicle control for 48 h.

Macrophages - T cell Trans well assay. Trans-well co-culture system using 0.4 μm cell inserts were used. The insert pore size didn't allow any cell migration from the top chamber to the bottom one or *vice versa*. PBMC (from healthy individuals obtained from blood bank Versiti) derived

monocyte were isolated using positive selection with CD14 magnetic columns (Miltenyi) and grown in 24 well plate. The monocytes were polarized to M0 macrophages using hMCSF and polymyxin B treatment for 5 days. PBMC derived naïve T cells were grown separately as a suspension culture and treated with oxylipin 10-HOME (100 µM) or vehicle control for 48h as described above. Following which, T cells were isolated and seeded on the top chamber of the insert in the following ratio (1:2) T-cells: macrophage. The vehicle treated culture of $CD4^+$ T-cells were simultaneously incubated under the same condition with ratios of 1:2 (T-cells: macrophage). Cells were grown in this co-culture system for 48 h(8). Afterwards, the bottom layer of macrophages were harvested and flowcytometry was performed.

RNA-Seq. RNA extraction**:** Homogenization of the tissue was done with BeadBug 6 homogenizer (Benchmark Scientific) in a cold room. Frozen tissue cores were transferred into 2ml prefilled tubes containing 3mm zirconium beads (Benchmark Scientific, cat# D1032-30), 350ul RLT Lysis Buffer (from the kit) and 2-Mercaptoethanol per kit instructions. Homogenization conditions: 4000 rpm for 45 seconds was repeated 2 times with 90 seconds rest time between repeats. Extraction process was done per kit instructions. DNA was eluted with 80 µl elution buffer and RNA with 30 µl RNase- free water. Library preparation: The bulk RNAseq libraries were generated using the TruSeq Stranded mRNA kit protocol. In brief, first the integrity of the RNA was checked with Agilent Tapestation. The mRNA was purified from 1µg of total RNA for each sample, after cDNA synthesis and adapter ligation, the library was amplified with 12 rounds of PCR. Bioinformatic analyses: 70-cycle Illumina NextSeq 500 paired-end reads were trimmed using fastp (version 0.20.1) with parameters "-l 17 --detect_adapter_for_pe -g -p"(9). The resulting reads were mapped against GRCh38 using HISAT2 (version 2.1.0) with parameters "--rna-strandness F"(10). HISAT uses Bowtie2, which is based on the Burrows-Wheeler transform algorithm, for sequence alignment and allows for mapping across exon junctions(11). Read counts for each gene were created using feature counts from the Subread package (version 1.6.4) with the parameters "-O -

M --primary –largest overlap -s 2 -B" and Gencode v36 as the annotation(12, 13). Differential expression analysis was performed using the DESeq2 package (version 1.30.0) in R/Bioconductor (R version 4.0.3)(14). The lists of genes differentially expressed were filtered by an adjusted p-value < 0.05. Two working files were generated to perform both analyses using the two datasets (BII *vs* non BII, BII *vs* normal). These contain one column including all gene ID annotations identified by the bioinformatics tools. This column was generated according to the annotations provided in the annotation file: original gene IDs (ENSEMBLE, GENEBANK) and mapped with human.

Tbet ZsGreen mice lineage studies: The mice were immune intact and fluoresced green upon activation of the Th1 responsive Tbet promoter(15-17). ZsGreen is the brightest available green fluorescent protein which is 4X brighter than eGFP. It is used for promoter-reporter studies. The animals were genotyped to verify the presence of transgene (**Supplementary Table 4, row # 4**). Intradermal injection of 10-HOME in the abdominal mammary fat pad (6.5 mg/kg body weight) every alternate day for ten days. The animals were euthanized. Blood was collected in heparin vials, RBC lysis was performed (Biolegend # 420302) stained with APC anti-mouse CD4 (clone GK1.5, Biolegend # 100411, 0.5 µg/ml) and assessed for CD4+ ZsGreen+ (Tbet) cells.

Murine Treadmill Endurance test.

Conditioning of mice to run on treadmill: C57BL/6 female mice were pre-conditioned with the treadmill over 5 days as described in(18),. For this, the animals were placed on the stationary belt to become accustomed to the apparatus. On subsequent days, the treadmill was placed on the lowest speed setting, and the mouse was allowed to walk or run slowly for 5–15 min for 5 days. Longer durations and/or higher speeds were avoided so as not to induce training adaptations. An aversive stimulus was introduced by placing an electric shock grid at the back of the treadmill

where the shock is not sufficient to harm the animal. Animals which stopped, touched the shock grid and were thus stimulated to run again. Mice unwilling to run after several training sessions were excluded from the study.

Endurance test: On completion of training, animals were injected with 10-HOME or vehicle in mammary pad 10-HOME (6.5 mg/kg body weight), every alternate day for ten days. Post- three days of final injection, endurance test was performed. The mice were made to run at 80 m/min for a period of 10 minutes. Fatigue has quantified on two parameters (a) number of times stopped during run and (b) number of times aversive stimulation (contact with shock grid) happened.

Quantitative RT PCR. Breast tissue was pulverized using tissue pulverizer (6770 Freezer/Mill) and total RNA was extracted using miRVana (Thermo Fisher Scientific). cDNA was made using SuperScript™ III First-Strand Synthesis System (Invitrogen) or SuperScript™ VILO™ cDNA Synthesis Kit (Invitrogen). Quantitative or real-time PCR (Sybr Green) approach was used for mRNA quantification (5, 19-23). Primer sequences used in this study are provided in (**Supplementary Table 4**).

Mass cytometric (CyToF) immunoassay. Human PBMCs cells were stained for mass cytometry, as described by Bodenmiller et al. (24) Each sample was resuspended to 2- 3 10^6 cells per 200 µl of Maxpar PBS in FACS tubes and incubated for 1–2 min at room temperature (RT) with 4000:1 parts Cisplatin-198Pt (Standard BioTools). The reaction was blocked with 1:5 parts CM, followed immediately by fixation. Every wash with Maxpar PBS was performed at 800*g, RT. We normalized cell volume (100 ml) before adding surface Ab mixture. After permeabilization (15 min on ice, we washed the cells twice with Maxpar PBS and normalized cell

volume again before adding intracellular Ab mixture. Only 20-min RT incubations of Cell-ID Intercalator-Ir (Standard BioTools) were performed (25).

CyToF setup and sample acquisition: A Helios CyTOF system by Standard BioTools (San Francisco, CA) was used to acquire all samples. A Helios WB Injector was installed. The sample line, capillary and nebulizer were assembled and checked for clogs. The system was started up and plasma ignited. DI Millipore water was run through the system for at least 15 minutes to prime the plasma. A full tuning procedure using CyTOF Tuning Solution (Standard Bio Tools #201072) was performed. A Bead Sensitivity Test was then performed using EQ Four Element Calibration Beads (Standard Bio Tools #201078) to verify tuning and check oxidation levels. Finally, Maxpar Cell Acquisition Solution (Standard Bio Tools #201240) was run through the system for 15 minutes. Received samples were transferred to a 15mL Falcon tube and 2mL of Maxpar Cell Staining Buffer (Standard Bio Tools #201068) was added to each. They were centrifuged for 5 minutes at 800g. The excess liquid was decanted and another 2mL of CSB was added and centrifuged again. After decanting, 2mL of Maxpar Cell Acquisition Solution (Standard Bio Tools # 201240) was added, centrifuged, and decanted. CAS was added and the previous steps repeated for a total of four washes. After decanting, each sample was diluted with a solution of Beads and CAS (1:9), placed on the sample stage and acquisition started.

CyToF Data analyses: ViSNE allows the visualization of single cell data based on the t-Distributed Stochastic Neighbor Embedding (t-SNE) algorithm. Each file was gated to exclude calibration beads, cell aggregates, dead cells as described(26). Each cell is represented as a single dot in viSNE bi-axial scatter plot. To classify a given T cell type, we used a combination of markers(27). The generated viSNE maps helped in the phenotypic profiling of each Th cell subset with reference to all other cells. viSNE program from CYTOBANK using the default settings for number of iterations, perplexity, and theta, an unbiased modelindependent was applied to ∼8 × 10^5 $CD3^+$ $CD4^+$ T cells from each population (from each file, 60,000–80,000 events were analyzed) for T

cell markers(25, 28). viSNE map is colored from blue to red indicating the increase in expression. The 9 markers were used to define Th cell subet were CD183, CD194, CD196, T-BET, GATA-3. Additionally, to distinguish Th subset cell origin from tumorigenic, LAG3 and PD-1, CTLA-4, CD161 were used. Heat maps were generated using the default settings in CYTOBANK and used to calculate the percentage of Th cell subset population. FlowSOM minimal spanning tree (MST) clustering was done to identify T -cell subsets. Total T cell CYTOF data from the three cohorts were clustered into 8 nodes with subsequent automated meta-clustering into 25 T cells subsets (meta-clusters) using clustering channels and the FlowSOM algorithm. CD3, CD4, CD183, CD194, CD196, T-BET, GATA-3, IL17, CCR4, CCR6, FOXP 3, CD127, CD25, CTLA4, FOXP3 markers were used to define Th cell subset.

Bacterial cultures for oxylipin analysis:

Staphylococcus epidermidis (Winslow and Winslow) Evans (ATCC 35984) *Pseudomonas fluorescens* (ATCC 135925), *Acinetobacter sp* (ATCC 49139), *Sphingomonas sp* (ATCC 31461), *Enterobacter cloacae* (ATCC 13047), *Cutibacterium acnes* (ATCC 6919), *Corynebacterium tuberculostearicum* (ATCC 35692) were cultured in their respective culture media to an OD600=0.6. Bacterial suspensions were then centrifuged at 8000g for 15 min. The pellet was resuspended in M9 medium containing Oleic acid at the desired concentration of 1%(v/v). Bacteria were then cultured in M9 medium at 30 °C overnight, till they reached OD600=1.0. The supernatant of bacterial culture grown in M9 medium supplemented with 1% oleic acid was used to identify 10-HOME oxylipin derived from the diol synthase activity. The cultures were then centrifuged at 8000g for 15 min, the supernatant was recovered, and evaluated for the 10-HOME oxylipin by HPLC/MS analysis.

Bacterial 10-HOME oxylipin analysis:

After thawing on wet ice, aliquots of the bacterial supernatants (10 µl) were diluted with 990 µl PBS and vortexed. From this, 5 µl was further diluted with 45 µl PBS, 400 µl internal standard solution (125 pg/ml 10-HOME-d_5 in PBS/methanol 1:1, v/v), 50 µl ethanol and 1.45 ml water. Bacterial 10-HOME analyses were carried out through Cayman chemical. For this, solid phase extraction was performed using Strata-X 33 µm, 30 mg/well 96-well plates (Phenomenex; Torrance, CA) and a Pressure+96 nitrogen-driven positive-pressure manifold (Biotage, Charlotte, NC). Plates were pre-conditioned using 2 ml methanol, followed by 2 ml water. Samples were then loaded onto the plates and washed with 2 ml water/methanol 9:1 (v/v). The sorbent was then dried under nitrogen flow prior to extracts being eluted with 1 ml methanol into a glass-lined 96-well plate. Solvent was then evaporated using a SpeedVac Concentrator (Thermo Fisher Scientific, Waltham, MA). The dried lipid extracts were reconstituted with 50 µl water/acetonitrile 3:2 (v/v) containing 0.2% acetic acid and injected for analysis into the liquid chromatography/tandem mass spectrometry (LC/MS/MS) system (Sciex; Framingham, MA). Chromatographic separation was performed on an ACQUITY UPLC BEH Shield RP18 Column, 130Å, 1.7 µm, 2.1 x 100 mm, fitted with an ACQUITY UPLC BEH Shield RP18 VanGuard cartridge, 130Å, 1.7 µm, 2.1 x 5 mm (Waters, Milford, MA). The autosampler on the ExionLC AD system was used to deliver 10 µl of each reconstituted extract to the UPLC column. The binary pump flow rate was set at 0.5 ml/min using water/acetonitrile 3:2 (v/v) containing 0.2% acetic acid as mobile phase A, and acetonitrile/isopropanol 1:1 (v/v) as mobile phase B. The column was held at 40 °C, and equilibrated with 99.9% mobile phase A prior to injection. The binary pump was set for a linear gradient to 55% B in 3.5 min, then to 99% B over 0.5 min, and held for 0.5 min. It was then returned to 99.9% A over 0.05 min and re-equilibrated for 1.45 min. The UPLC system was coupled to a 6500+ triple-quadrupole mass spectrometer equipped with an electrospray ion source. The ion spray voltage was -4500 V, the gas temperature was 500 ºC, the entrance potential was -10 V, and the collision cell exit potential was -11 V. Collision, curtain, and ion source

gas pressures were set at 8, 20, and 30 psi, respectively. Signals were monitored in the MRM mode, using a declustering potential of -60 V, a collision energy of -30 V, and the *m/z* transitions 297.2 -> 155.1 for 10-HOME and 302.2 -> 155.1 for 10-HOME-d_5, with dwell times of 10 ms. Peak areas (ion intensity *vs* elution time) were integrated using commercial software (MultiQuant, Sciex). Analyte response areas were normalized for each sample using the isotopically labelled internal standard peak area. These area ratios were then interpolated into a calibration curve made using authentic standards serially diluted from a 1 μg/ml stock solution and extracted alongside samples.

Dose response curve for *S. epidermidis*:

Staphylococcus epidermidis was cultured in LB medium to an OD600=0.6. Bacterial suspensions were then centrifuged at 8000g for 15 min. The pellet was resuspended in M9 medium containing Oleic acid at the desired concentration of 0.2, 0.4, and 1%(v/v). Bacteria were then cultured in Oleic acid containing M9 medium at 30 °C, for 4 and 12 h. The supernatant of bacterial culture grown in M9 medium supplemented with 1% oleic acid was used to identify 10-HOME oxylipin derived from the diol synthase activity. The cultures were then centrifuged at 8000g for 15 min, the supernatant was recovered, and evaluated for the 10-HOME oxylipin by LC/MS/MS analysis as described in the previous section.

Ingenuity Pathways Analysis. Pathway analysis was performed using Ingenuity Pathway Analysis™ (IPA; Qiagen, Inc., USA) software(29-31). The normalized mRNA counts (gene expression datasets) were exported from TAC software and uploaded into IPA. A cutoff filter of student t-test $p < 0.05$ was used to maximize the number of genes in the analysis (recommended by IPA to enhance the analysis power and accuracy). The IPA Core Analysis function followed by the Comparison Analysis function was used to compare the effect of the differentially expressed

genes (DEG) across BII and non BII samples. The Canonical Pathway function was used to enrich The T cell subset, and Macrophage by the DE genes between BII and non BII samples. The sign and magnitude of the Z-scores are indicative of the predicted strength and direction. The T helper subset, and Macrophage pathways were enriched and (ranked by Z-score) are shown here based on the canonical pathways activated/inactivated in BII specimens vs non BII specimens BY DEGs generated in the core analysis of Ingenuity pathway analysis tool. Orange color pathways are activated ($Z > 2$) and blue color pathways are inactivated ($Z < -2$). Height of the bar graphs indicates -log (p-value) and line graph showing the ratio of list genes found in T-helper cell activation pathway, and macrophage over the total number of genes in that pathway.

Quantification and statistical analysis

The distribution of the increased abundance of Th subtypes were evaluated for normality using Shapiro-Wilk test and Q-Q plot. Descriptive statistics by groups (BII, non-BII, and normal) were calculated using mean (Standard deviation) for normally distributed data and median (inter-quartile range) for those deviating from normality. Two-sample test of proportions with two tailed z-tests were used to analyze the hypothesis that the proportion of different type of bacteria in the BII group was significantly different that the proportion of the biofilm infection in the non-BII and normal group. Non-parametric bivariate analyses were performed using Kruskal-Wallis test followed by Dunn's test for pairwise comparisons with Benjamini-Hochberg multiple testing adjusted p-values to minimize the false discovery rate. In the model building steps, we examined the correlation between mean duration of implant and age and found age was a significantly correlated with the duration and thus, to avoid the issue of multicollinearity, we excluded duration in the model. Adjusting for the confounding effect of age in multivariable model, we used non-parametric regression with bootstrapped standard error obtained from 500 repetitions to compare the estimates of Th1 across the groups. We also examined 10-HOME which was found to be normally distributed and hence comparison between non BII and BII was done using student's t-

test and age-adjusted regression with bootstrapped standard errors. To examine the estimates of % bacterial abundance and % NGS Staphylococcus using 10-HOME as the predictor, we performed bivariate and age-adjusted bootstrapped ordinary least squares (OLS) and non-parametric regressions as appropriate. Ranks of the bacterial abundance was presented using spider-plot and bubble plot by groups.

In vivo studies (murine) involving drug intervention (10-HOME) were blinded. For the murine models, to compare the estimates of T Cells between 10-HOME and vehicle (Control) group, we performed bivariate analysis using Wilcoxon-Rank Sum test and two tailed t-tests as appropriate. In addition, we also compared the IFN-Ɣ and IL-10 obtained from ELISA analysis across BII, non-BII and normal participants using bivariate Kruskal-Wallis analyses and age-adjusted bootstrapped non-parametric regressions after confirming the distributional assumption of these variables. All hypotheses were tested at 0.05 level of significance using Stata/MP 16.1. Visually data are presented as mean ± SEM (*in vivo*) or ± SD (*in vitro*) as reported in figure legends.

References (Supplementary Methods)

1. Barki KG, Das A, Dixith S, Ghatak PD, Mathew-Steiner S, Schwab E, et al. Electric Field Based Dressing Disrupts Mixed-Species Bacterial Biofilm Infection and Restores Functional Wound Healing. *Ann Surg.* 2019;269(4):756-66.
2. Roy S, Elgharably H, Sinha M, Ganesh K, Chaney S, Mann E, et al. Mixed-species biofilm compromises wound healing by disrupting epidermal barrier function. *J Pathol.* 2014;233(4):331-43.
3. Deng B, Ghatak S, Sarkar S, Singh K, Das Ghatak P, Mathew-Steiner SS, et al. Novel Bacterial Diversity and Fragmented eDNA Identified in Hyperbiofilm-Forming Pseudomonas aeruginosa Rugose Small Colony Variant. *iScience.* 2020;23(2):100827.

4. Roy S, Santra S, Das A, Dixith S, Sinha M, Ghatak S, et al. Staphylococcus aureus Biofilm Infection Compromises Wound Healing by Causing Deficiencies in Granulation Tissue Collagen. *Ann Surg.* 2020;271(6):1174-85.

5. Sinha M, Sen CK, Singh K, Das A, Ghatak S, Rhea B, et al. Direct conversion of injury-site myeloid cells to fibroblast-like cells of granulation tissue. *Nat Commun.* 2018;9(1):936.

6. Shen W, Falahati R, Stark R, Leitenberg D, and Ladisch S. Modulation of CD4 Th cell differentiation by ganglioside GD1a in vitro. *J Immunol.* 2005;175(8):4927-34.

7. Ganusov VV, Milutinovic D, and De Boer RJ. IL-2 regulates expansion of CD4+ T cell populations by affecting cell death: insights from modeling CFSE data. *J Immunol.* 2007;179(2):950-7.

8. Chatterjee S, Das S, Chakraborty P, Manna A, Chatterjee M, and Choudhuri SK. Myeloid derived suppressor cells (MDSCs) can induce the generation of Th17 response from naive CD4+ T cells. *Immunobiology.* 2013;218(5):718-24.

9. Chen S, Zhou Y, Chen Y, and Gu J. fastp: an ultra-fast all-in-one FASTQ preprocessor. *Bioinformatics.* 2018;34(17):i884-i90.

10. Kim D, Langmead B, and Salzberg SL. HISAT: a fast spliced aligner with low memory requirements. *Nat Methods.* 2015;12(4):357-60.

11. Langmead B, and Salzberg SL. Fast gapped-read alignment with Bowtie 2. *Nat Methods.* 2012;9(4):357-9.

12. Liao Y, Smyth GK, and Shi W. featureCounts: an efficient general purpose program for assigning sequence reads to genomic features. *Bioinformatics.* 2014;30(7):923-30.

13. Harrow J, Frankish A, Gonzalez JM, Tapanari E, Diekhans M, Kokocinski F, et al. GENCODE: the reference human genome annotation for The ENCODE Project. *Genome Res.* 2012;22(9):1760-74.

14. Love MI, Huber W, and Anders S. Moderated estimation of fold change and dispersion for RNA-seq data with DESeq2. *Genome Biol.* 2014;15(12):550.

15. Kawabe T, Jankovic D, Kawabe S, Huang Y, Lee PH, Yamane H, et al. Memory-phenotype CD4(+) T cells spontaneously generated under steady-state conditions exert innate TH1-like effector function. *Sci Immunol.* 2017;2(12).

16. Hu G, Tang Q, Sharma S, Yu F, Escobar TM, Muljo SA, et al. Expression and regulation of intergenic long noncoding RNAs during T cell development and differentiation. *Nat Immunol.* 2013;14(11):1190-8.

17. Zhu J, Jankovic D, Oler AJ, Wei G, Sharma S, Hu G, et al. The transcription factor T-bet is induced by multiple pathways and prevents an endogenous Th2 cell program during Th1 cell responses. *Immunity.* 2012;37(4):660-73.

18. Poole DC, Copp SW, Colburn TD, Craig JC, Allen DL, Sturek M, et al. Guidelines for animal exercise and training protocols for cardiovascular studies. *Am J Physiol Heart Circ Physiol.* 2020;318(5):H1100-H38.

19. Das A, Ganesh K, Khanna S, Sen CK, and Roy S. Engulfment of apoptotic cells by macrophages: a role of microRNA-21 in the resolution of wound inflammation. *J Immunol.* 2014;192(3):1120-9.

20. Das A, Ghatak S, Sinha M, Chaffee S, Ahmed NS, Parinandi NL, et al. Correction of MFG-E8 Resolves Inflammation and Promotes Cutaneous Wound Healing in Diabetes. *J Immunol.* 2016;196(12):5089-100.

21. Ganesh K, Das A, Dickerson R, Khanna S, Parinandi NL, Gordillo GM, et al. Prostaglandin E(2) induces oncostatin M expression in human chronic wound macrophages through Axl receptor tyrosine kinase pathway. *J Immunol.* 2012;189(5):2563-73.

22. Khanna S, Biswas S, Shang Y, Collard E, Azad A, Kauh C, et al. Macrophage dysfunction impairs resolution of inflammation in the wounds of diabetic mice. *PLoS One.* 2010;5(3):e9539.

23. Das A, Datta S, Roche E, Chaffee S, Jose E, Shi L, et al. Novel mechanisms of Collagenase Santyl Ointment (CSO) in wound macrophage polarization and resolution of wound inflammation. *Sci Rep.* 2018;8(1):1696.

24. Bodenmiller B, Zunder ER, Finck R, Chen TJ, Savig ES, Bruggner RV, et al. Multiplexed mass cytometry profiling of cellular states perturbed by small-molecule regulators. *Nat Biotechnol.* 2012;30(9):858-67.

25. Kunicki MA, Amaya Hernandez LC, Davis KL, Bacchetta R, and Roncarolo MG. Identity and Diversity of Human Peripheral Th and T Regulatory Cells Defined by Single-Cell Mass Cytometry. *J Immunol.* 2018;200(1):336-46.

26. Amir el AD, Davis KL, Tadmor MD, Simonds EF, Levine JH, Bendall SC, et al. viSNE enables visualization of high dimensional single-cell data and reveals phenotypic heterogeneity of leukemia. *Nat Biotechnol.* 2013;31(6):545-52.

27. Sen N, Mukherjee G, and Arvin AM. Single cell mass cytometry reveals remodeling of human T cell phenotypes by varicella zoster virus. *Methods.* 2015;90:85-94.

28. Greenplate AR, McClanahan DD, Oberholtzer BK, Doxie DB, Roe CE, Diggins KE, et al. Computational Immune Monitoring Reveals Abnormal Double-Negative T Cells Present across Human Tumor Types. *Cancer Immunol Res.* 2019;7(1):86-99.

29. Rustagi Y, Abouhashem AS, Verma P, Verma SS, Hernandez E, Liu S, et al. Endothelial Phospholipase Cgamma2 Improves Outcomes of Diabetic Ischemic Limb Rescue Following VEGF Therapy. *Diabetes.* 2022;71(5):1149-65.

30. Singh K, Sinha M, Pal D, Tabasum S, Gnyawali SC, Khona D, et al. Cutaneous Epithelial to Mesenchymal Transition Activator ZEB1 Regulates Wound Angiogenesis and Closure in a Glycemic Status-Dependent Manner. *Diabetes.* 2019;68(11):2175-90.

31. Singh K, Pal D, Sinha M, Ghatak S, Gnyawali SC, Khanna S, et al. Epigenetic Modification of MicroRNA-200b Contributes to Diabetic Vasculopathy. *Mol Ther.* 2017;25(12):2689-704.
